# Supplementary material for: Mechano-regulation of GLP-1 production by Piezo1 in intestinal L cells
Source: eLife. 2024 Nov 7;13:RP97854. doi: 10.7554/eLife.97854 (PMC11542922; doi:10.7554/eLife.97854)
Supplement: Figure 1—source data 1. [file elife-97854-fig1-data1.zip › Figure1-source data 1.pdf]

Figure 1B

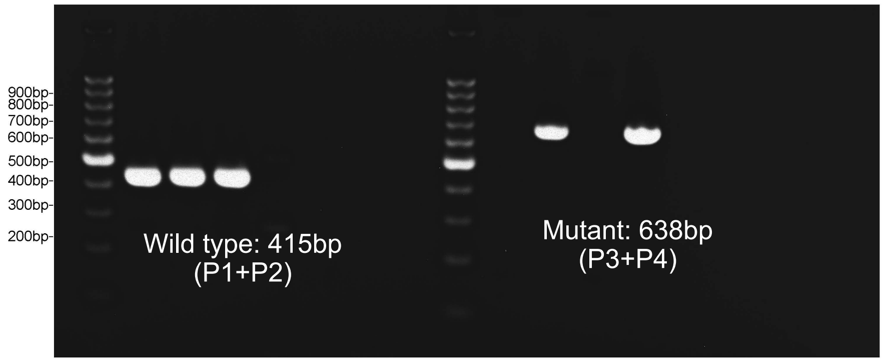
900bp-
800bp-
700bp-
600bp-
500bp-
400bp-
300bp-
200bp-
Wild type: 415bp
(P1+P2)
Mutant: 638bp
(P3+P4)

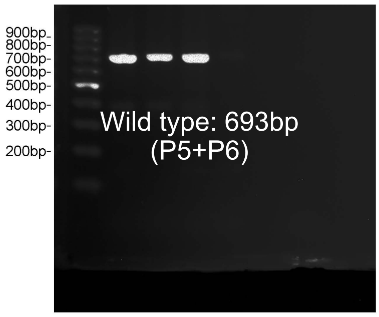
900bp-
800bp-
700bp-
600bp-
500bp-
400bp-
300bp-
200bp-
Wild type: 693bp
(P5+P6)

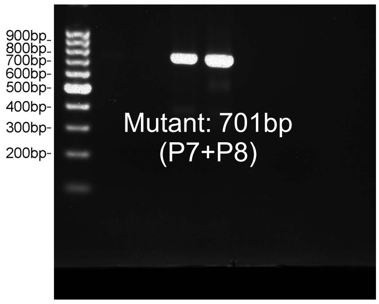
900bp-
800bp-
700bp-
600bp-
500bp-
400bp-
300bp-
200bp-
Mutant: 701bp
(P7+P8)

Figure 1C

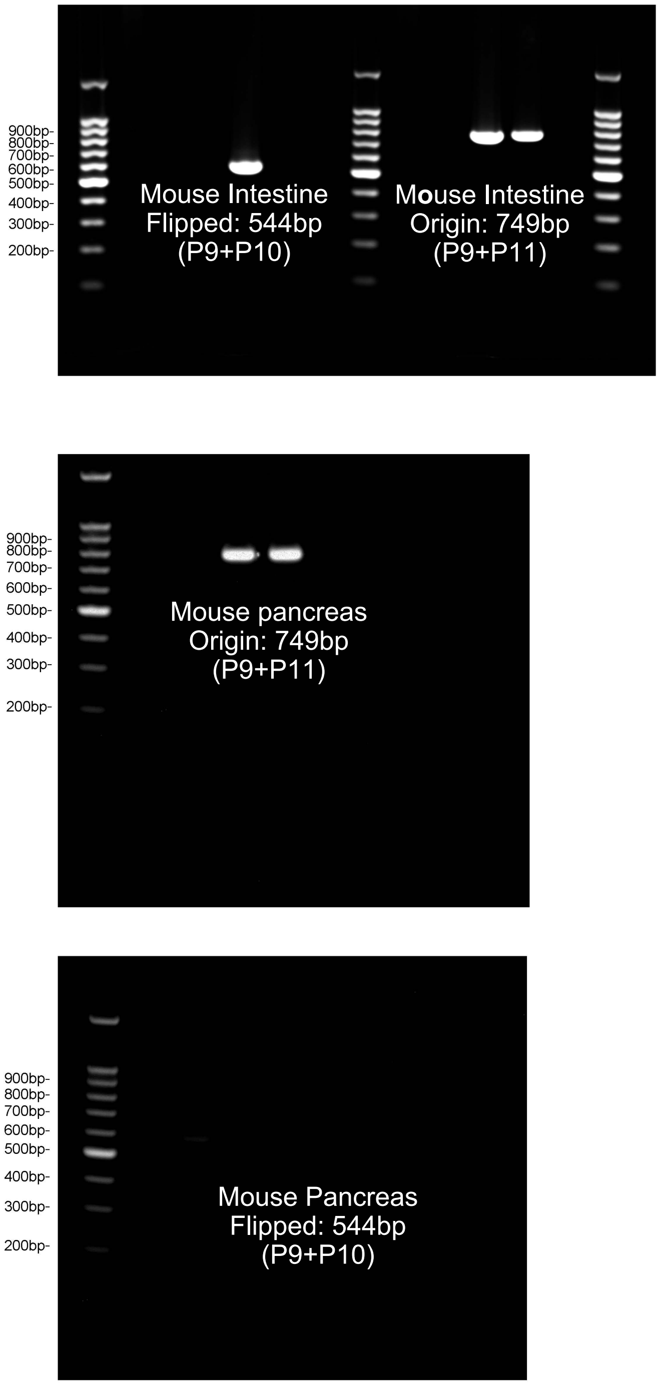
900bp-
800bp-
700bp-
600bp-
500bp-
400bp-
300bp-
200bp-
Mouse Intestine
Flipped: 544bp
(P9+P10)
Mouse Intestine
Origin: 749bp
(P9+P11)
900bp-
800bp-
700bp-
600bp-
500bp-
400bp-
300bp-
200bp-
Mouse pancreas
Origin: 749bp
(P9+P11)
900bp-
800bp-
700bp-
600bp-
500bp-
400bp-
300bp-
200bp-
Mouse Pancreas
Flipped: 544bp
(P9+P10)

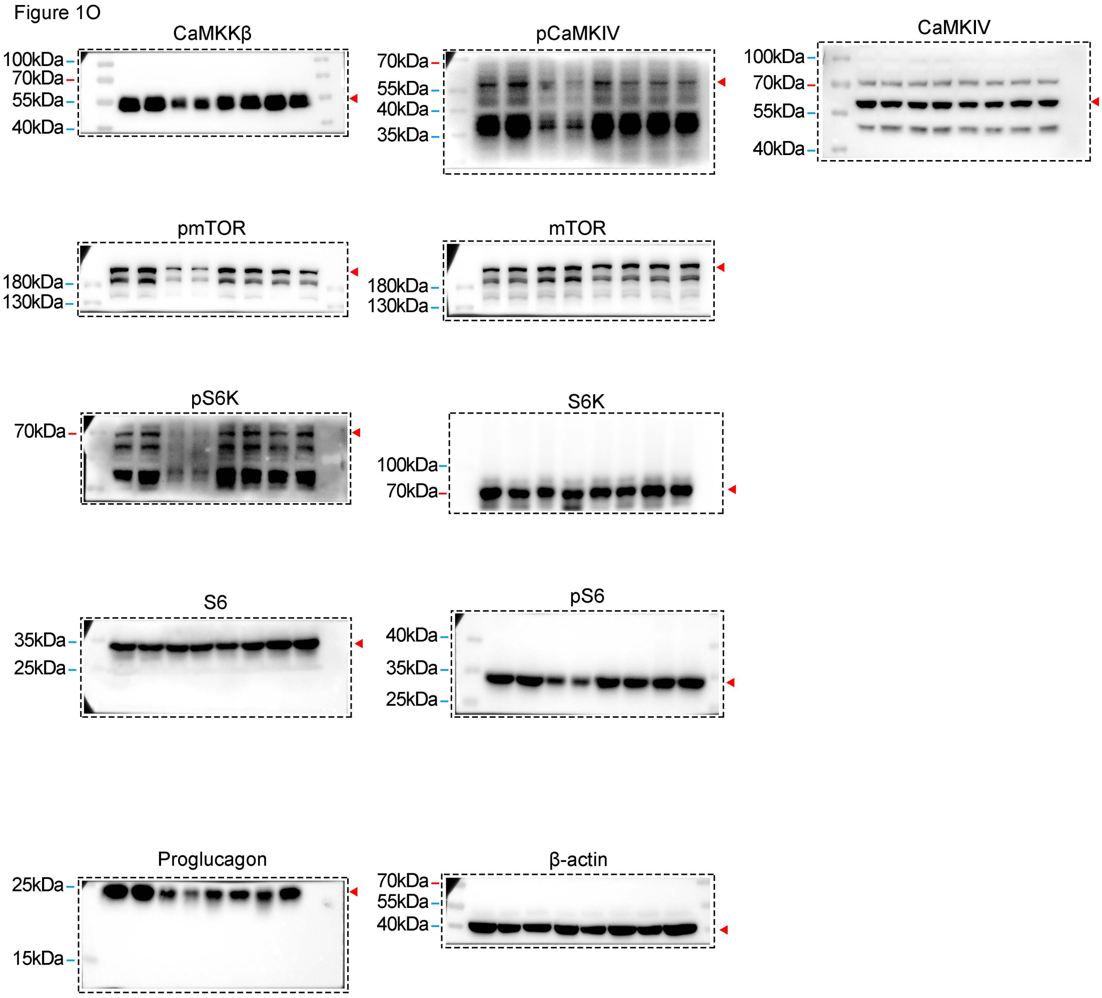

Figure 1, Source Data 1. Original membranes corresponding to Figure 1, panel B, C and O. Among them, the WB strips of figrue 1O are *Piezo1*$^{loxp/loxp}$ in lane 1, 2, *Piezo1* IntL-CKO in lane 3, 4, *Vil1*^{Flp} in lane 5, 6, and *Gcg*^{cre} in lane 7,8.
