# Supplementary figures and images for: Mechano-regulation of GLP-1 production by Piezo1 in intestinal L cells (part 1 of 2)
Source: eLife. 2024 Nov 7;13:RP97854. doi: 10.7554/eLife.97854 (PMC11542922; doi:10.7554/eLife.97854)

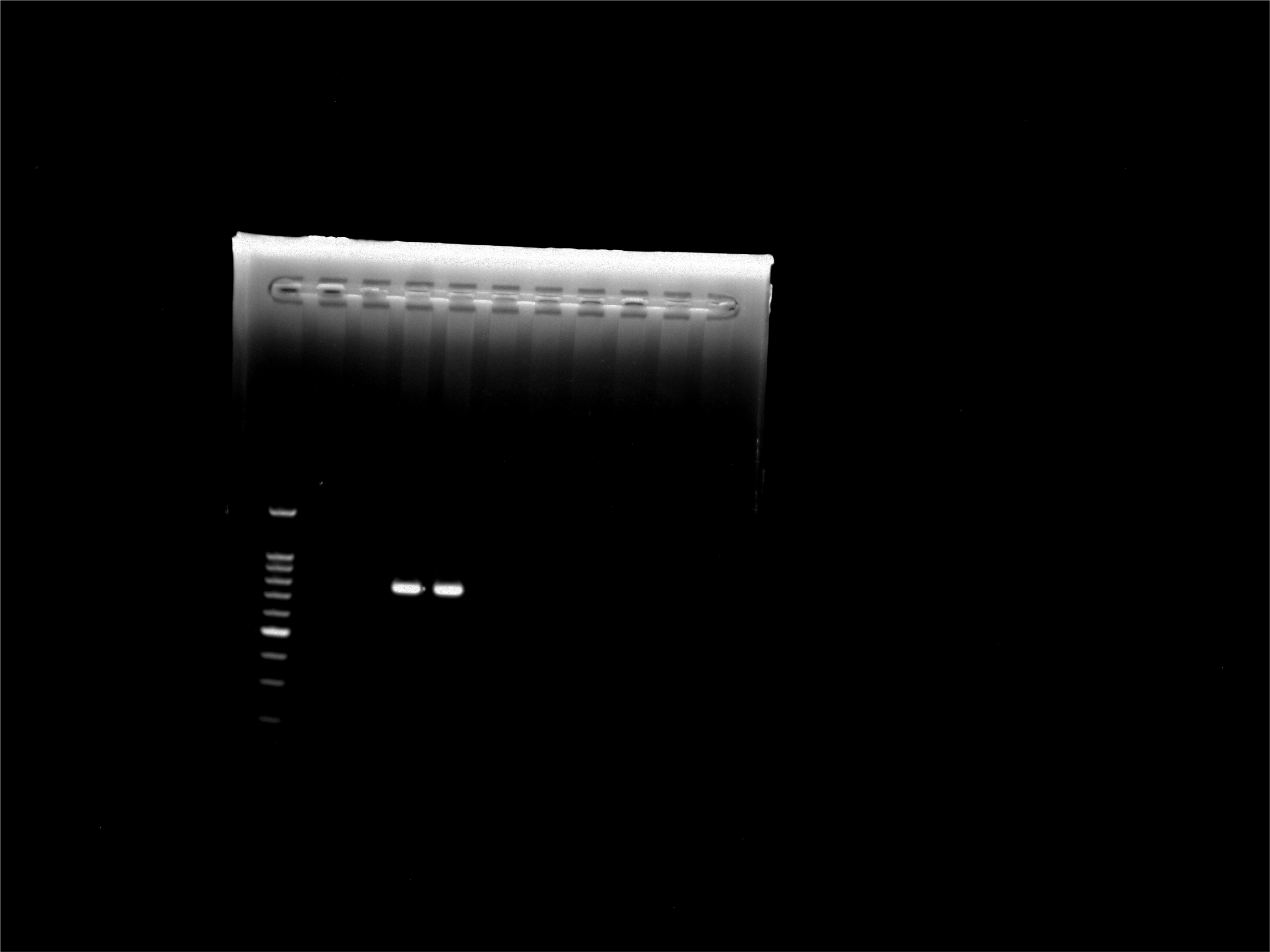

Supplement: Figure 1—source data 2. [file elife-97854-fig1-data2.zip › 2022-10-13_18-48-02 (E) (1).tif]

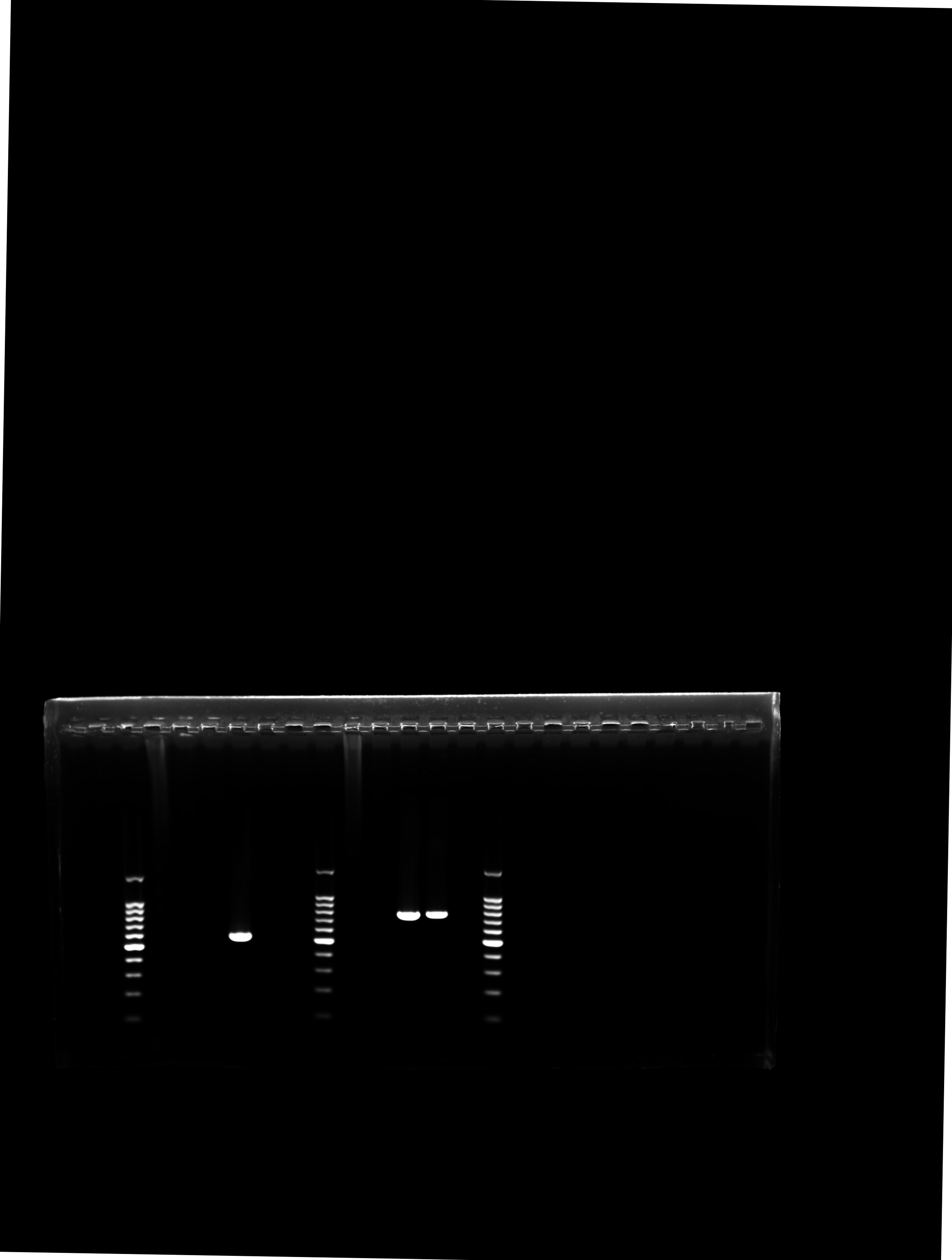

Supplement: Figure 1—source data 2. [file elife-97854-fig1-data2.zip › 20221028.1.tif]

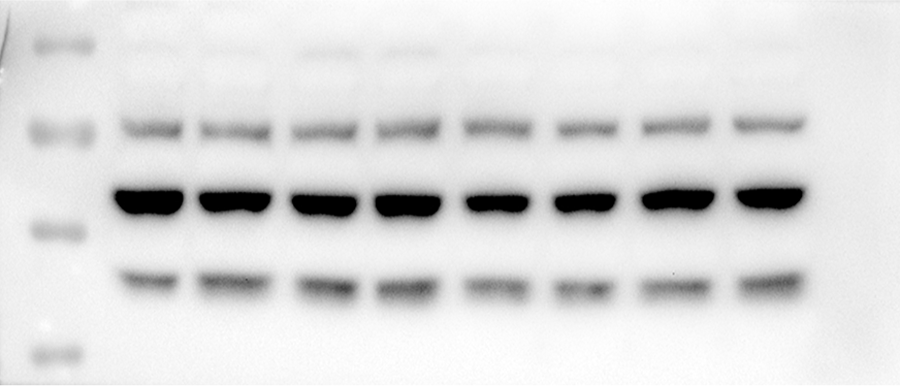

Supplement: Figure 1—source data 2. [file elife-97854-fig1-data2.zip › CaMKIV.tif]

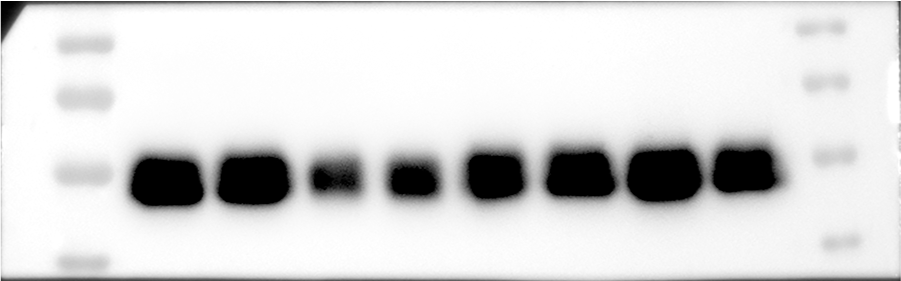

Supplement: Figure 1—source data 2. [file elife-97854-fig1-data2.zip › CaMKK╬▓.tif]

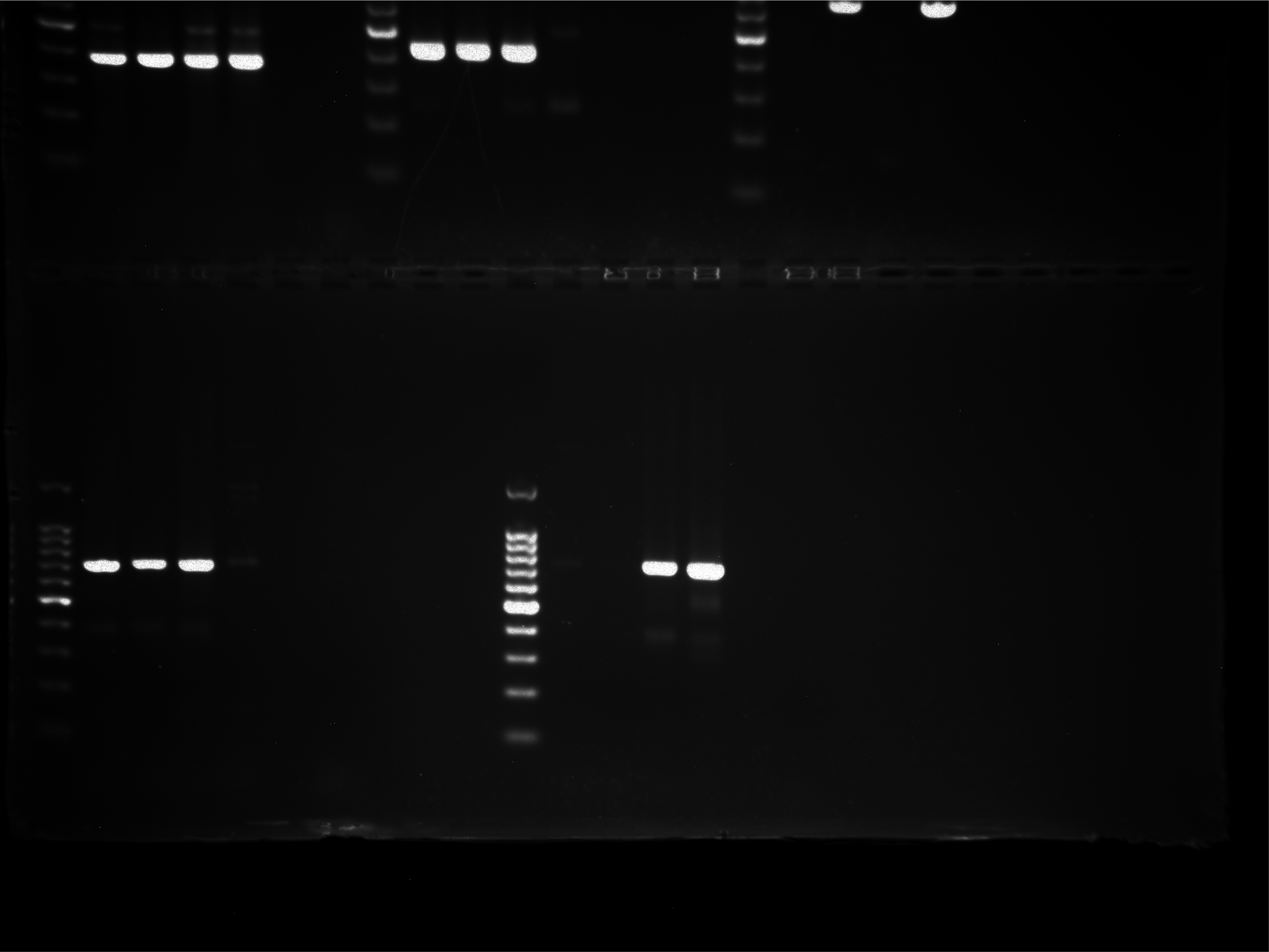

Supplement: Figure 1—source data 2. [file elife-97854-fig1-data2.zip › GCG-FRTσƒ║σ¢áΘë┤σ«Ü1.tif]

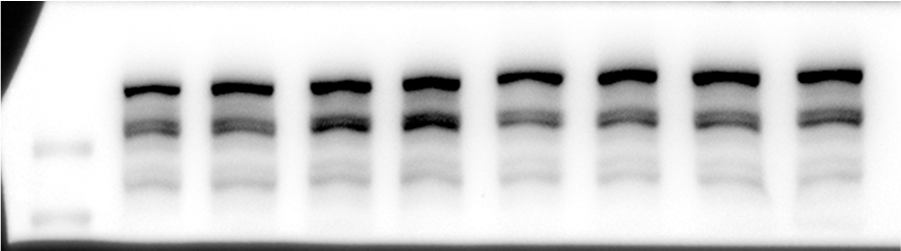

Supplement: Figure 1—source data 2. [file elife-97854-fig1-data2.zip › mTOR.tif]

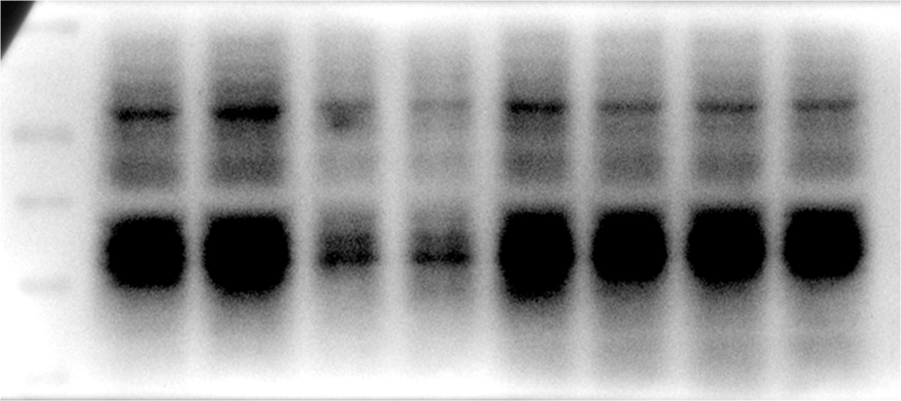

Supplement: Figure 1—source data 2. [file elife-97854-fig1-data2.zip › pCaMKIV.tif]

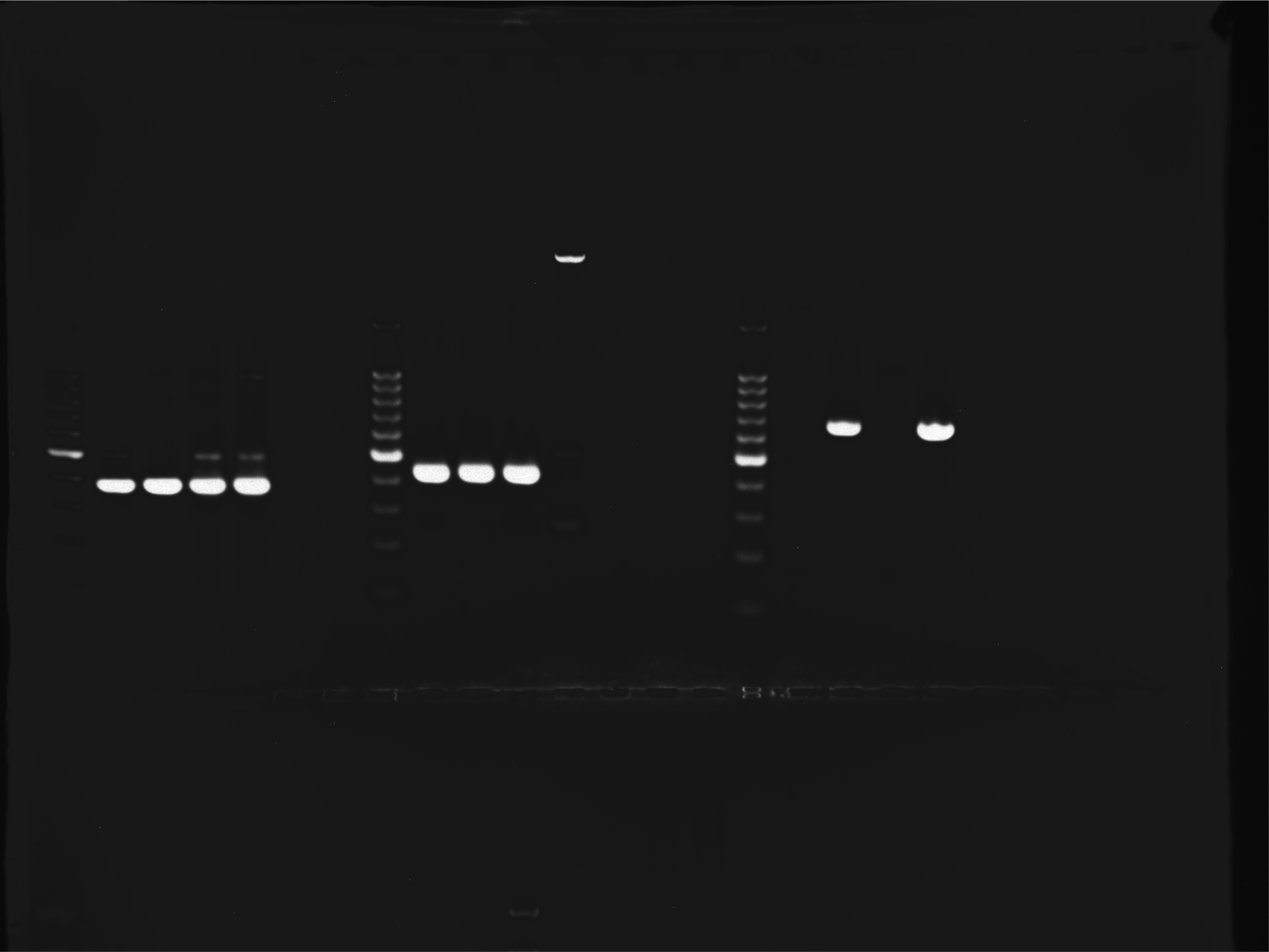

Supplement: Figure 1—source data 2. [file elife-97854-fig1-data2.zip › PL, vil-flpσƒ║σ¢áΘë┤σ«Ü1.tif]

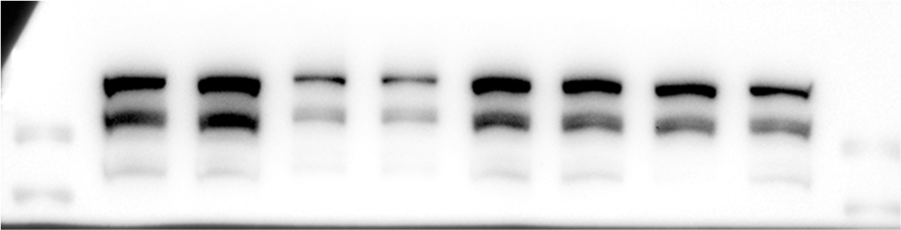

Supplement: Figure 1—source data 2. [file elife-97854-fig1-data2.zip › pmTOR.tif]

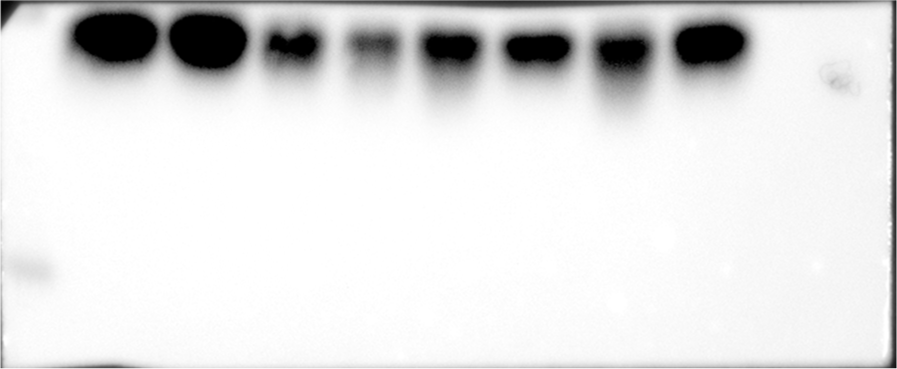

Supplement: Figure 1—source data 2. [file elife-97854-fig1-data2.zip › Proglucagen.tif]

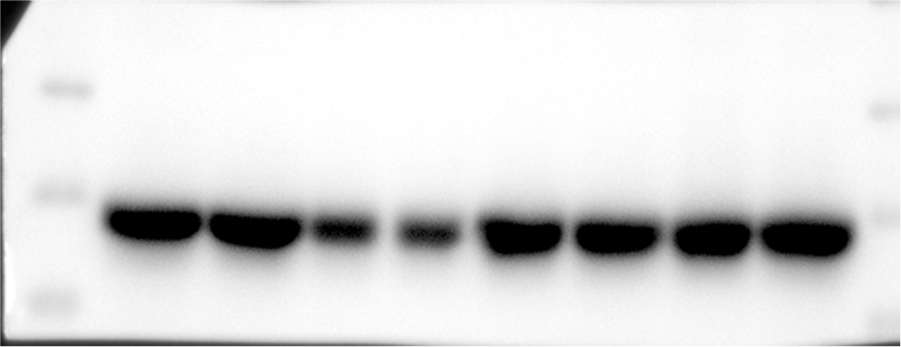

Supplement: Figure 1—source data 2. [file elife-97854-fig1-data2.zip › pS6.tif]

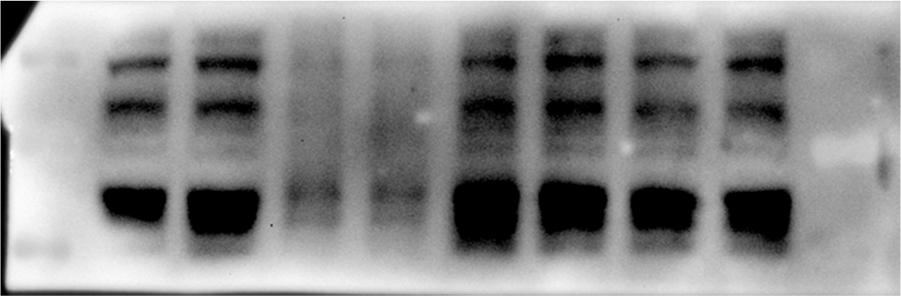

Supplement: Figure 1—source data 2. [file elife-97854-fig1-data2.zip › pS6K.tif]

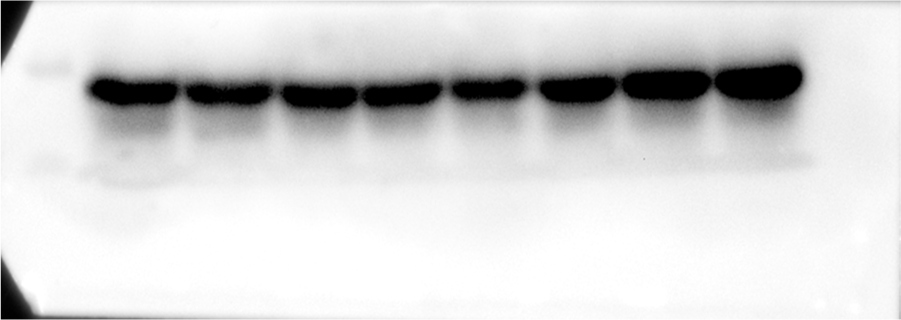

Supplement: Figure 1—source data 2. [file elife-97854-fig1-data2.zip › S6.tif]

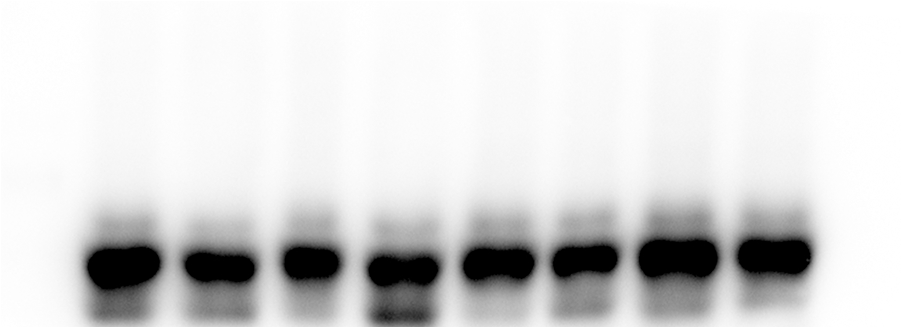

Supplement: Figure 1—source data 2. [file elife-97854-fig1-data2.zip › S6K.tif]

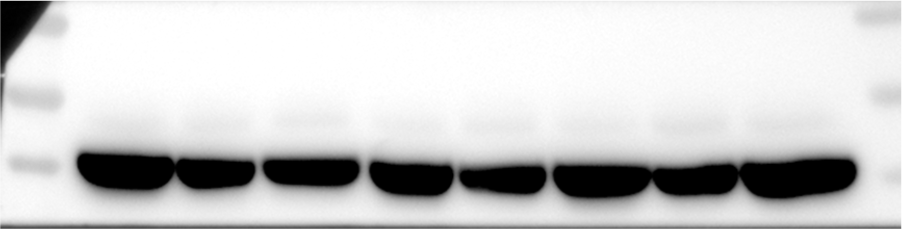

Supplement: Figure 1—source data 2. [file elife-97854-fig1-data2.zip › ╬▓-actin.tif]

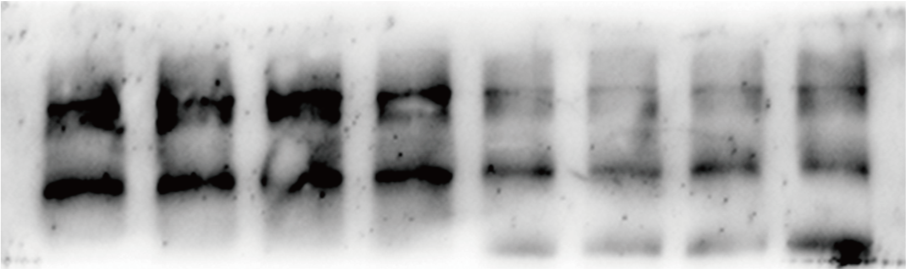

Supplement: Figure 1—figure supplement 1—source data 2. [file elife-97854-fig1-figsupp1-data2.zip › Piezo1-1E.tif]

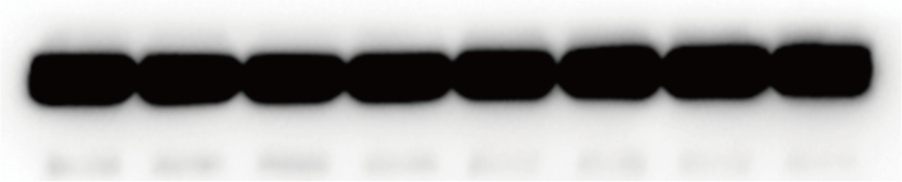

Supplement: Figure 1—figure supplement 1—source data 2. [file elife-97854-fig1-figsupp1-data2.zip › ╬▓-actin-1E.tif]

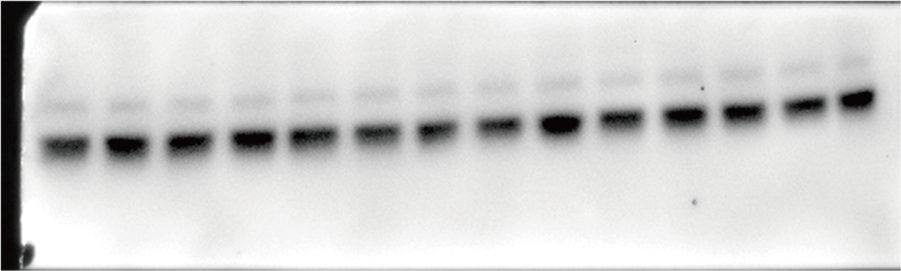

Supplement: Figure 1—figure supplement 3—source data 2. [file elife-97854-fig1-figsupp3-data2.zip › Proglucagon-3D.tif]

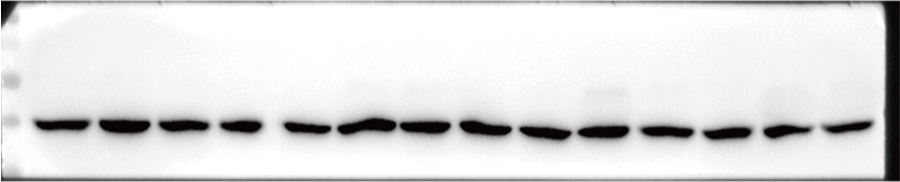

Supplement: Figure 1—figure supplement 3—source data 2. [file elife-97854-fig1-figsupp3-data2.zip › ╬▓-actin-3D.tif]

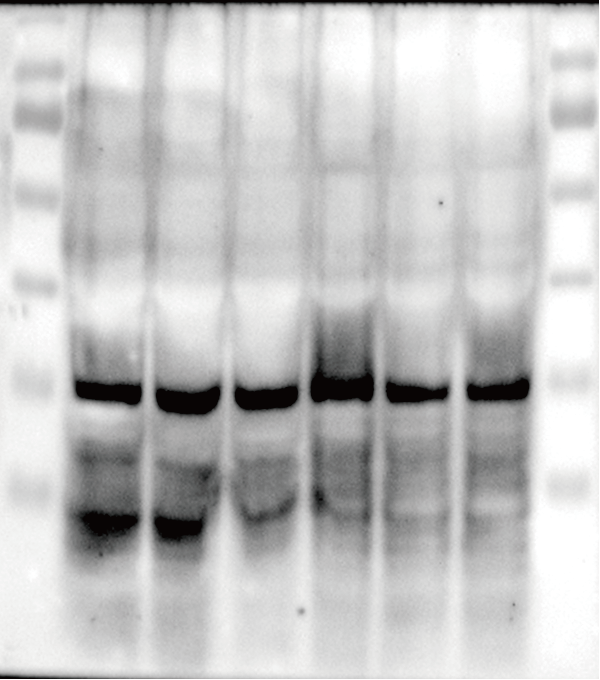

Supplement: Figure 1—figure supplement 8—source data 2. [file elife-97854-fig1-figsupp8-data2.zip › Figure 1-figure supplement 8-source data 2/Occludin-8C.tif]

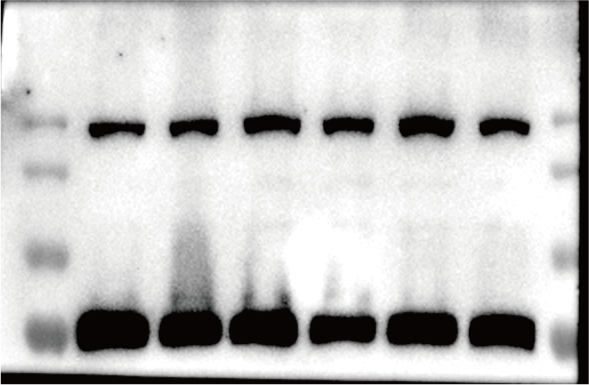

Supplement: Figure 1—figure supplement 8—source data 2. [file elife-97854-fig1-figsupp8-data2.zip › Figure 1-figure supplement 8-source data 2/ZO-1-8C.tif]

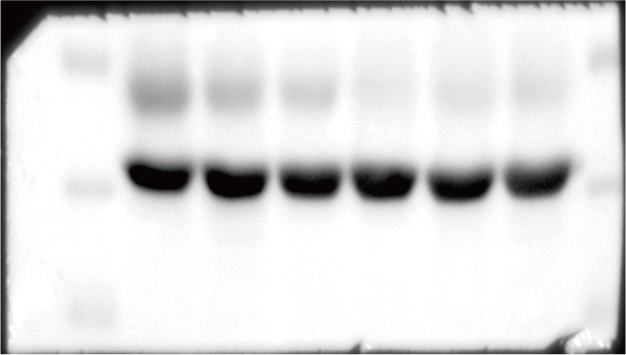

Supplement: Figure 1—figure supplement 8—source data 2. [file elife-97854-fig1-figsupp8-data2.zip › Figure 1-figure supplement 8-source data 2/β-actin-8C.tif]

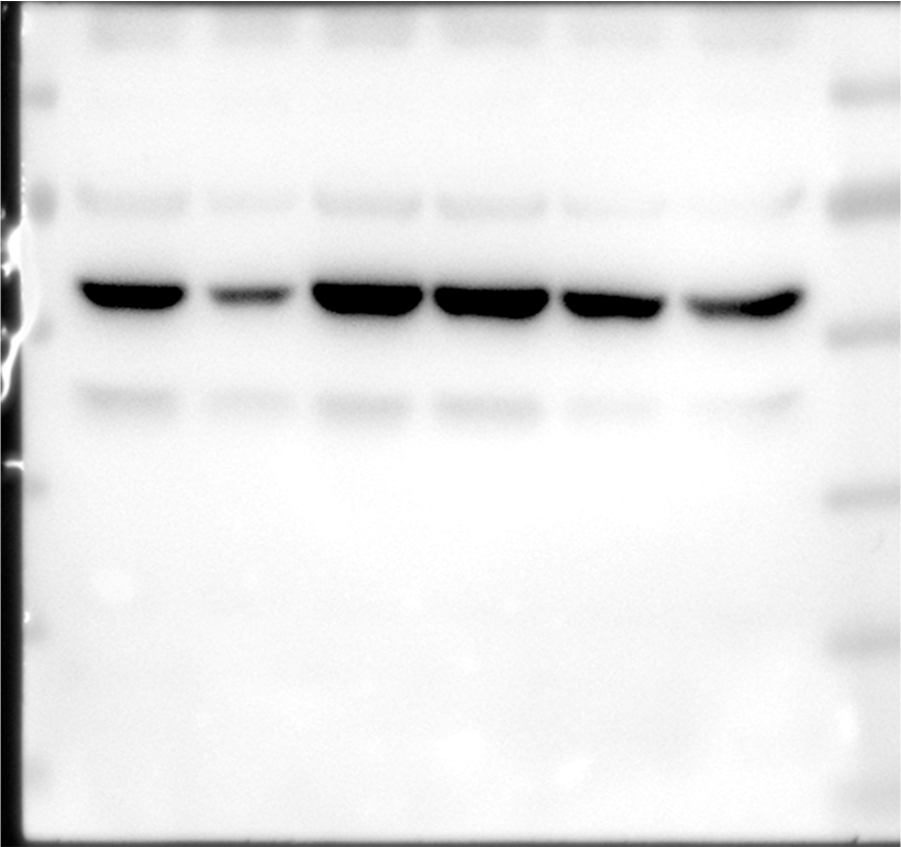

Supplement: Figure 2—source data 2. [file elife-97854-fig2-data2.zip › CaMKIV.tif]

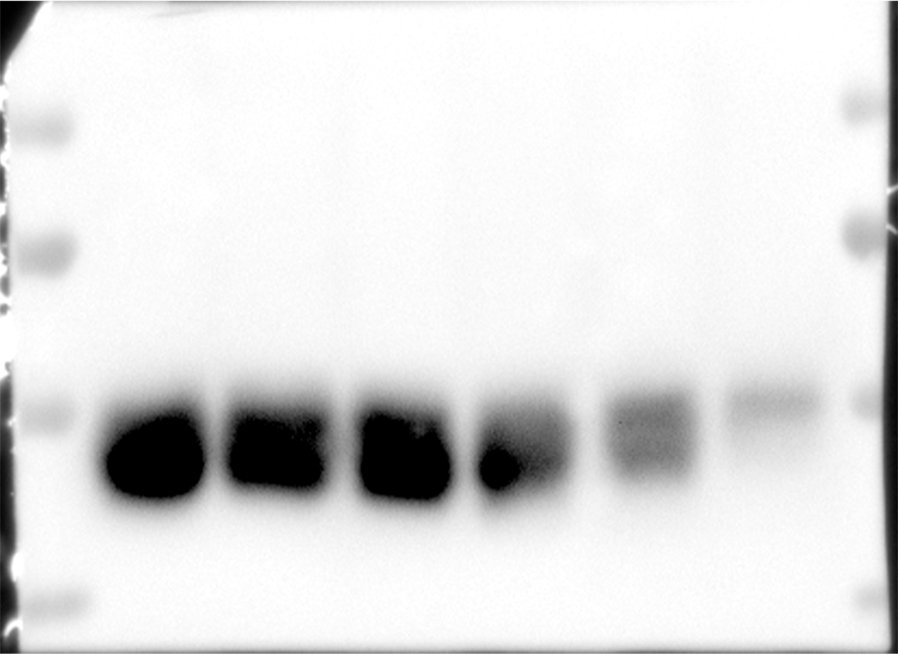

Supplement: Figure 2—source data 2. [file elife-97854-fig2-data2.zip › CaMKK╬▓.tif]

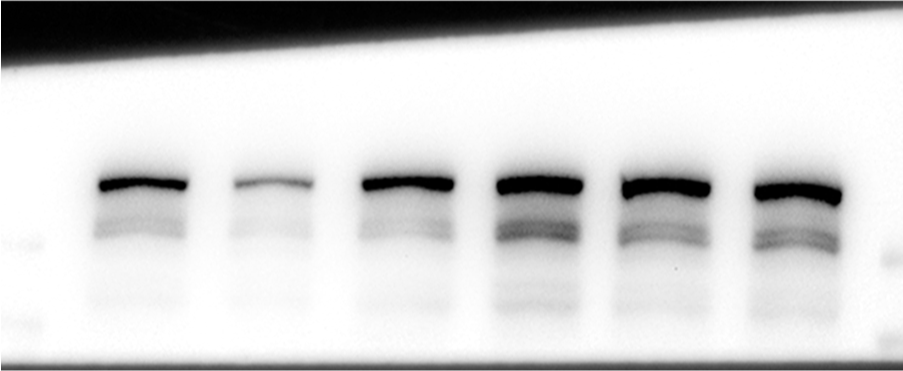

Supplement: Figure 2—source data 2. [file elife-97854-fig2-data2.zip › mTOR.tif]

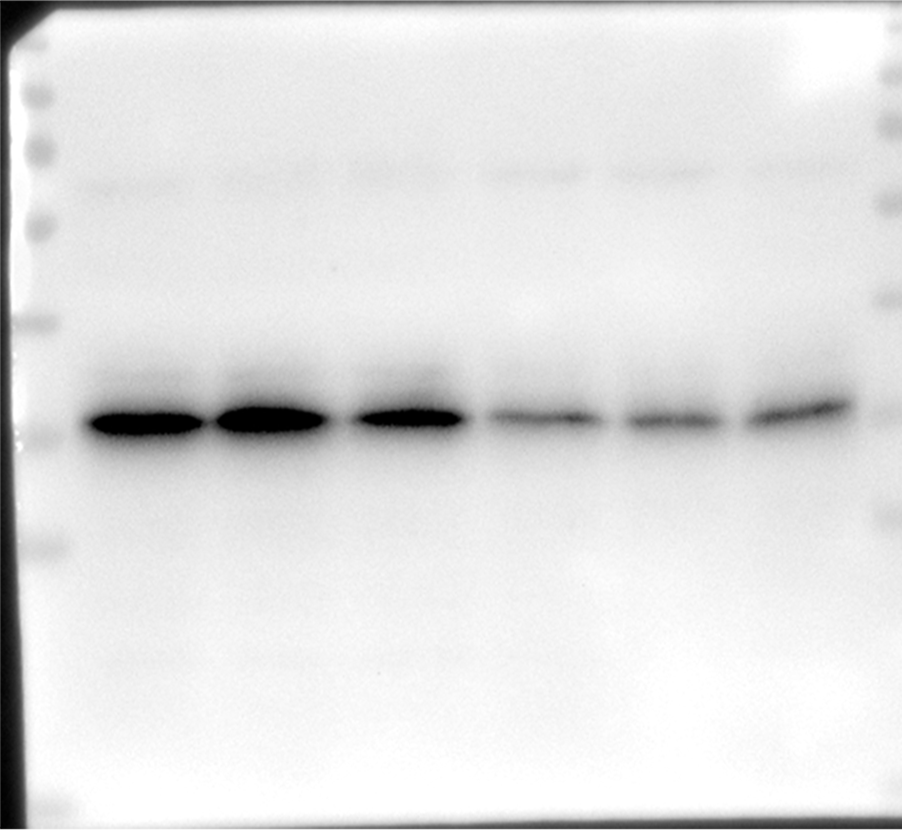

Supplement: Figure 2—source data 2. [file elife-97854-fig2-data2.zip › pCaMKIV.tif]

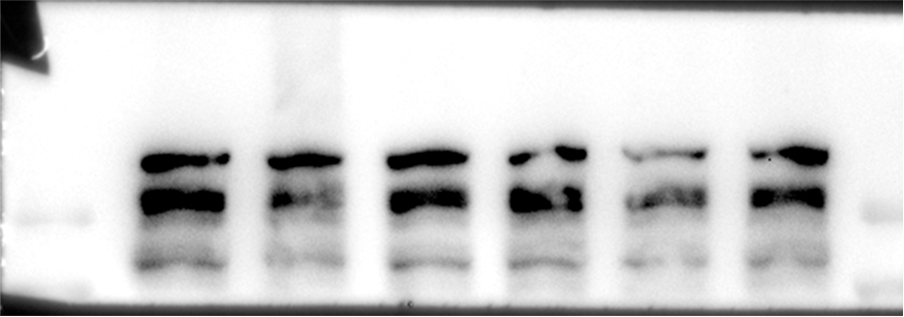

Supplement: Figure 2—source data 2. [file elife-97854-fig2-data2.zip › pmTOR.tif]

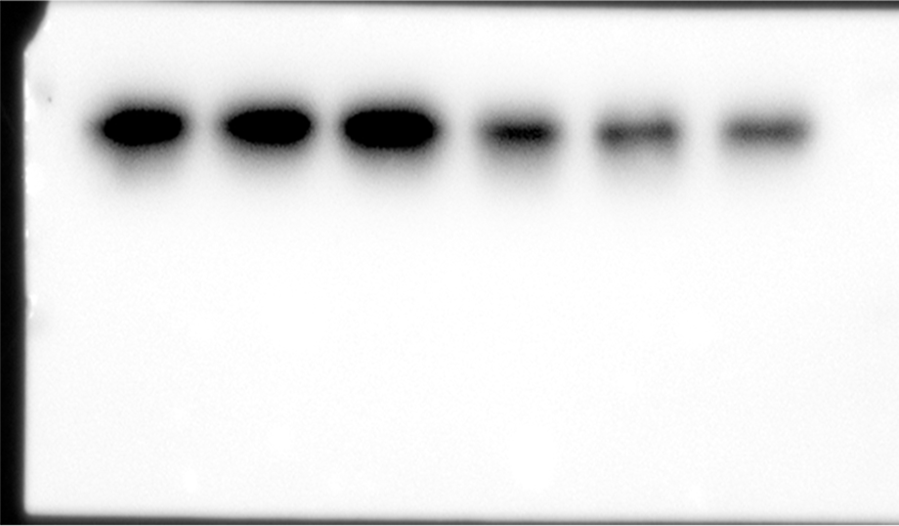

Supplement: Figure 2—source data 2. [file elife-97854-fig2-data2.zip › Proglucagen.tif]

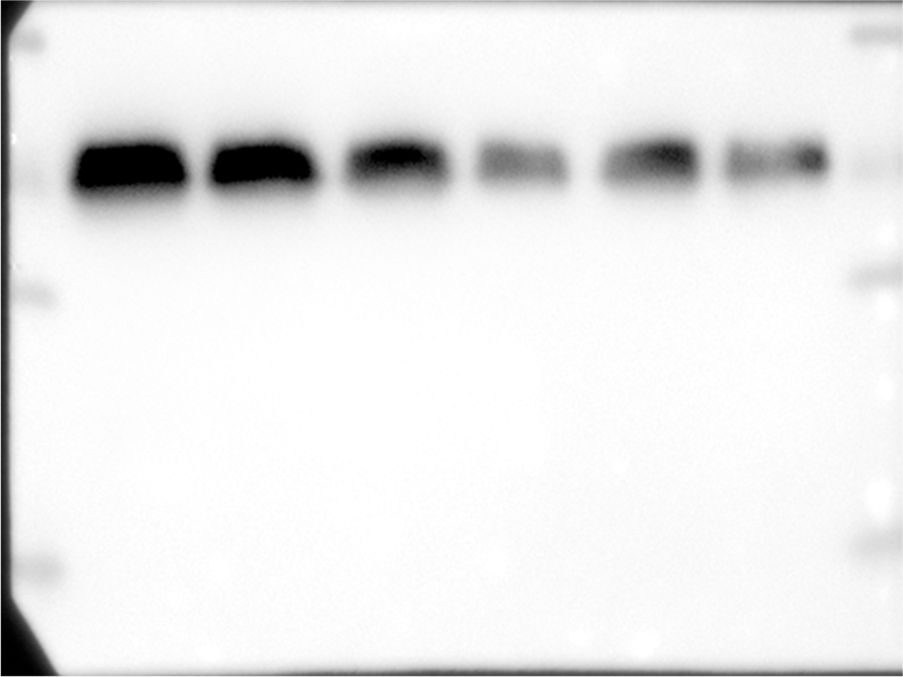

Supplement: Figure 2—source data 2. [file elife-97854-fig2-data2.zip › pS6.tif]

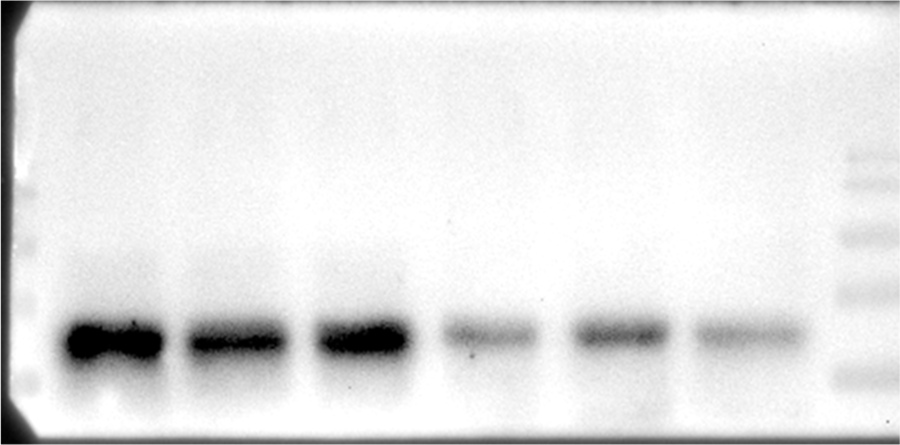

Supplement: Figure 2—source data 2. [file elife-97854-fig2-data2.zip › pS6K.tif]

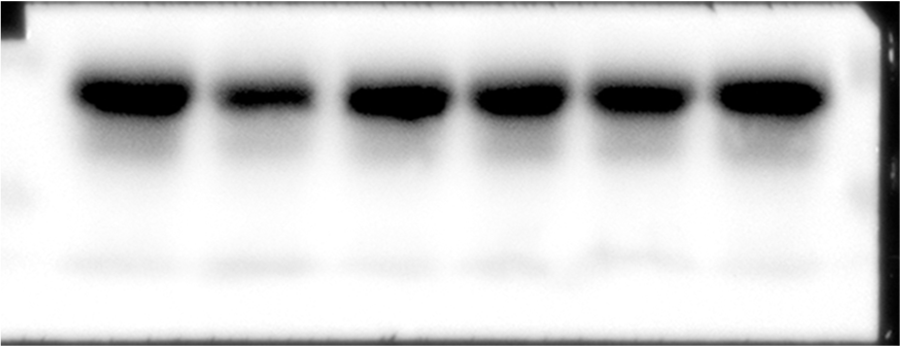

Supplement: Figure 2—source data 2. [file elife-97854-fig2-data2.zip › S6.tif]

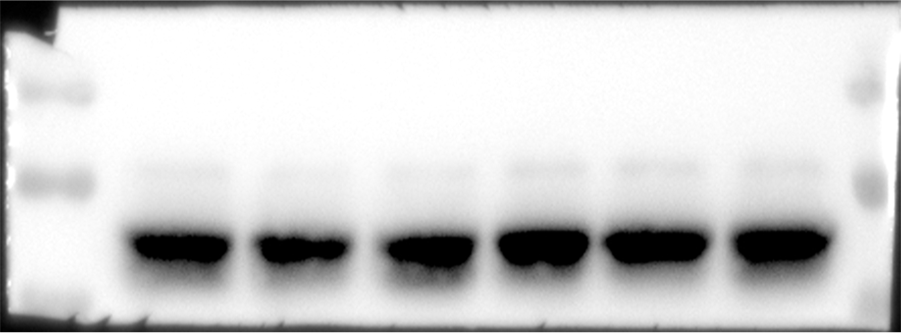

Supplement: Figure 2—source data 2. [file elife-97854-fig2-data2.zip › S6K.tif]

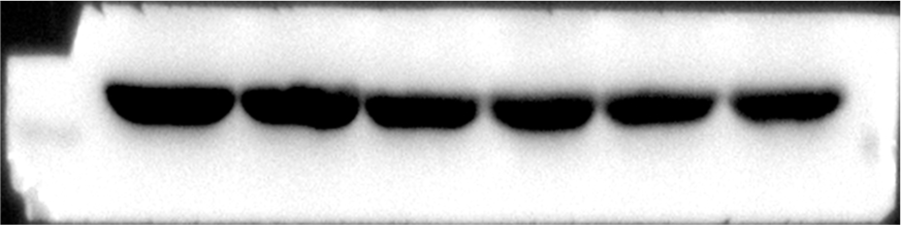

Supplement: Figure 2—source data 2. [file elife-97854-fig2-data2.zip › ╬▓-actin.tif]

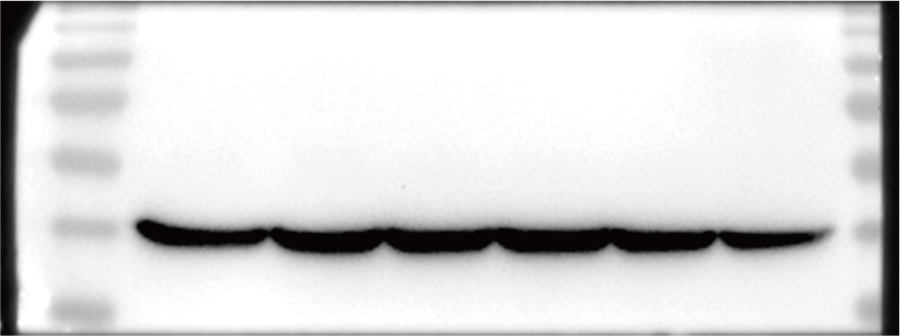

Supplement: Figure 2—figure supplement 1—source data 2. [file elife-97854-fig2-figsupp1-data2.zip › ╬▓-actin.tif]

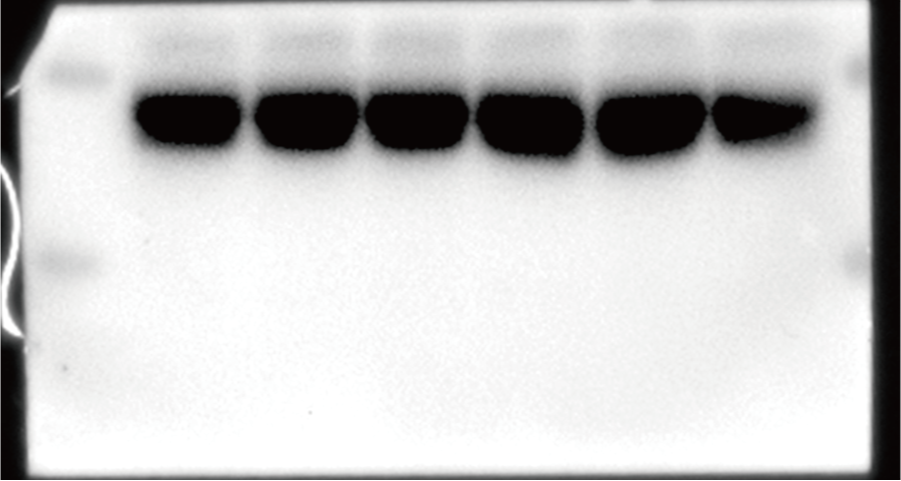

Supplement: Figure 2—figure supplement 1—source data 2. [file elife-97854-fig2-figsupp1-data2.zip › Proglucagon.tif]

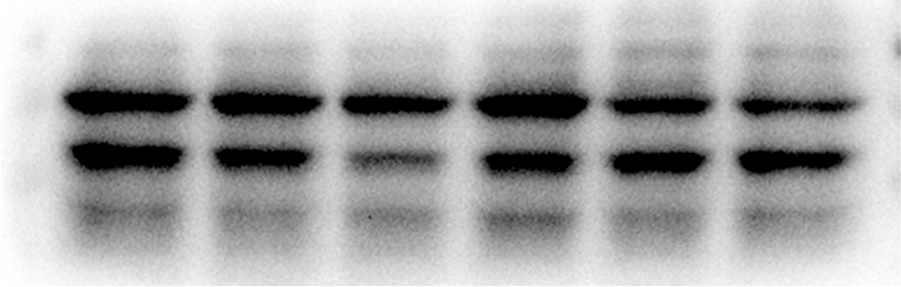

Supplement: Figure 3—source data 2. [file elife-97854-fig3-data2.zip › CaMKIV-3E.tif]

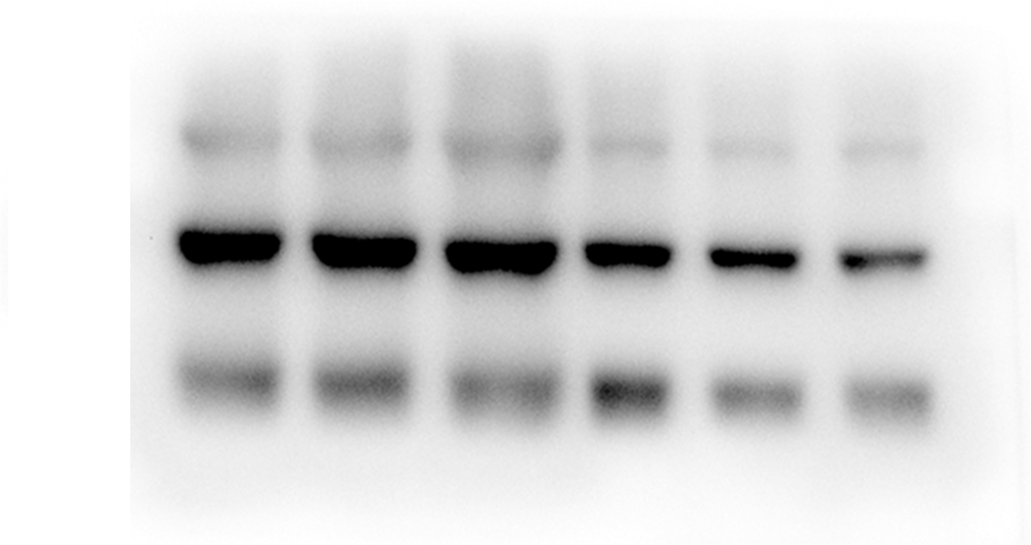

Supplement: Figure 3—source data 2. [file elife-97854-fig3-data2.zip › CaMKIV-3R.tif]

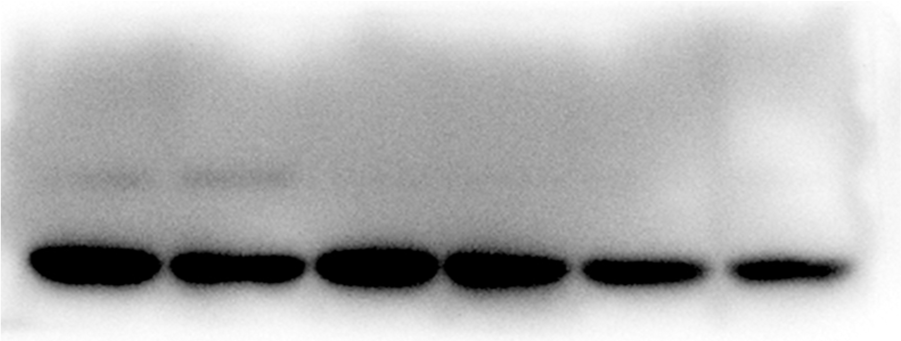

Supplement: Figure 3—source data 2. [file elife-97854-fig3-data2.zip › CaMKK╬▓-3E.tif]

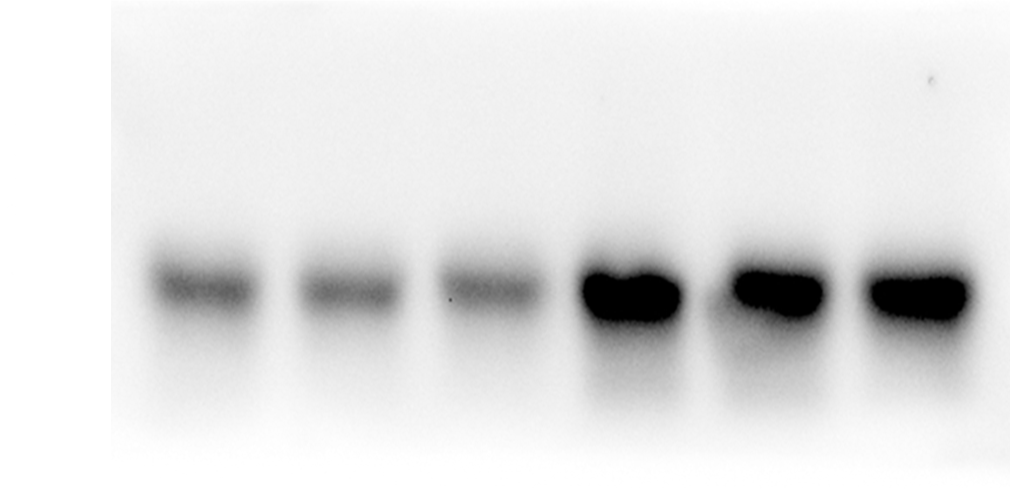

Supplement: Figure 3—source data 2. [file elife-97854-fig3-data2.zip › CaMKK╬▓-3R.tif]

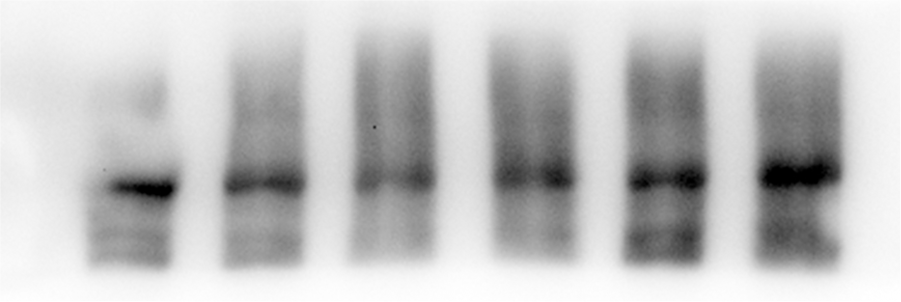

Supplement: Figure 3—source data 2. [file elife-97854-fig3-data2.zip › mTOR-3E.tif]

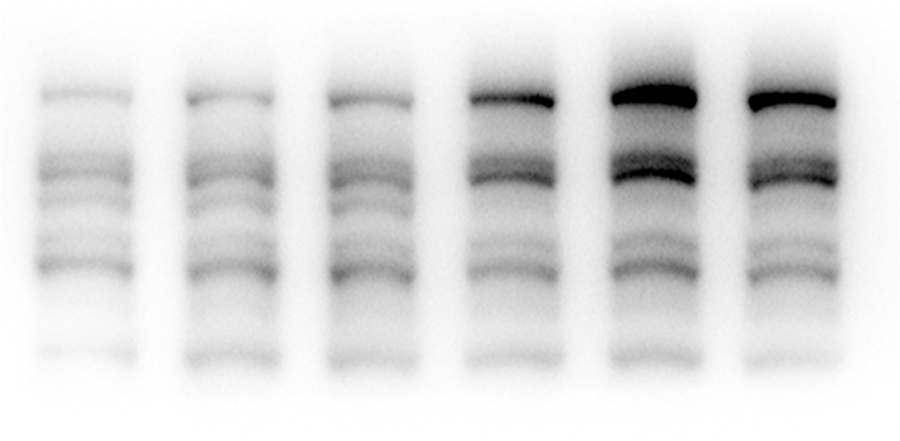

Supplement: Figure 3—source data 2. [file elife-97854-fig3-data2.zip › mTOR-3R.tif]

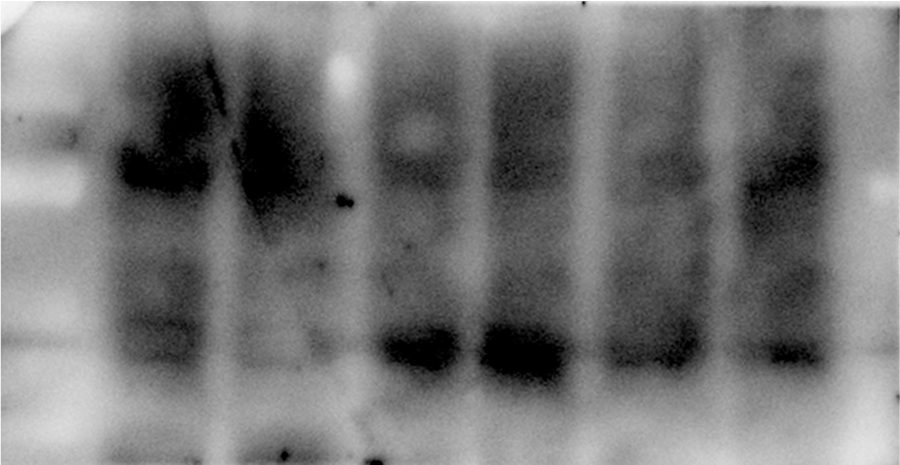

Supplement: Figure 3—source data 2. [file elife-97854-fig3-data2.zip › pCaMKIV-3E.tif]

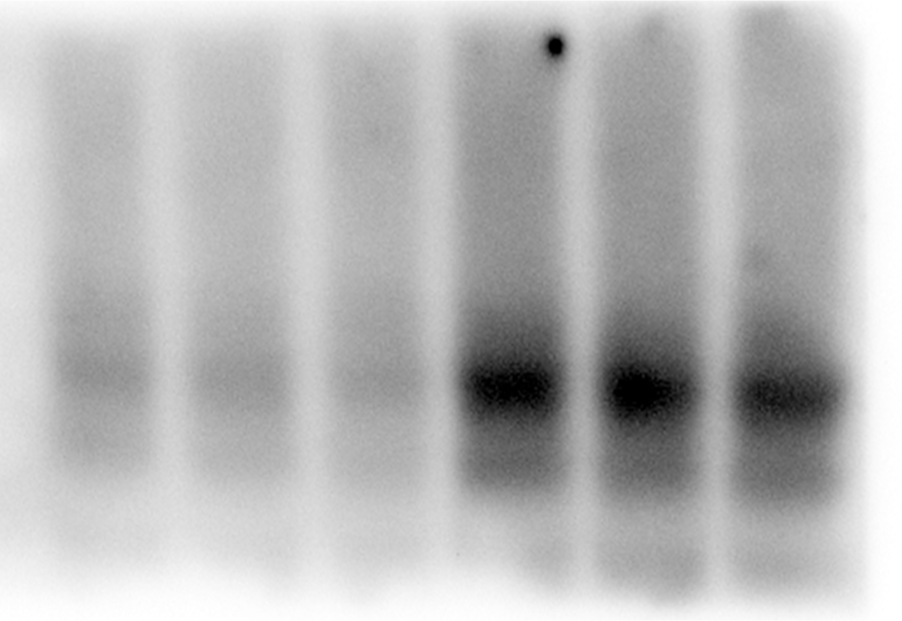

Supplement: Figure 3—source data 2. [file elife-97854-fig3-data2.zip › pCaMKIV-3R.tif]

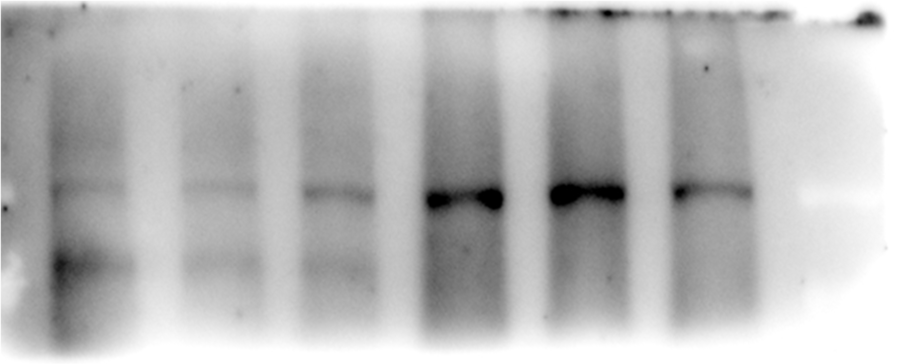

Supplement: Figure 3—source data 2. [file elife-97854-fig3-data2.zip › Piezo1-3R.tif]

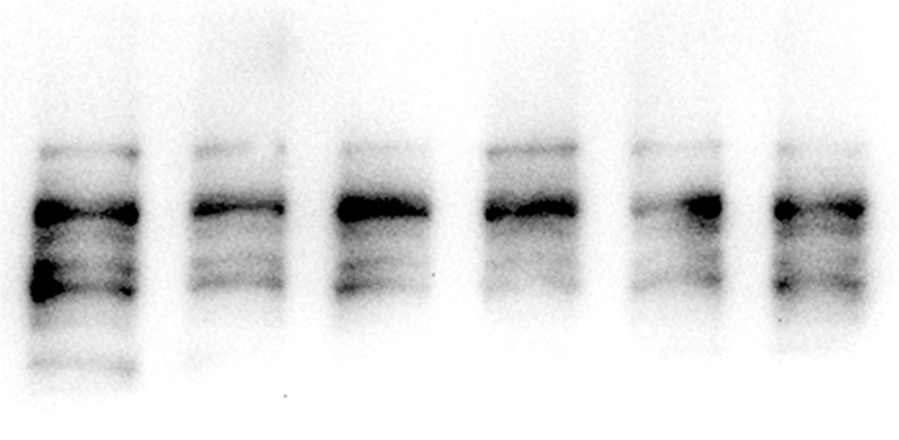

Supplement: Figure 3—source data 2. [file elife-97854-fig3-data2.zip › pmTOR-3E.tif]

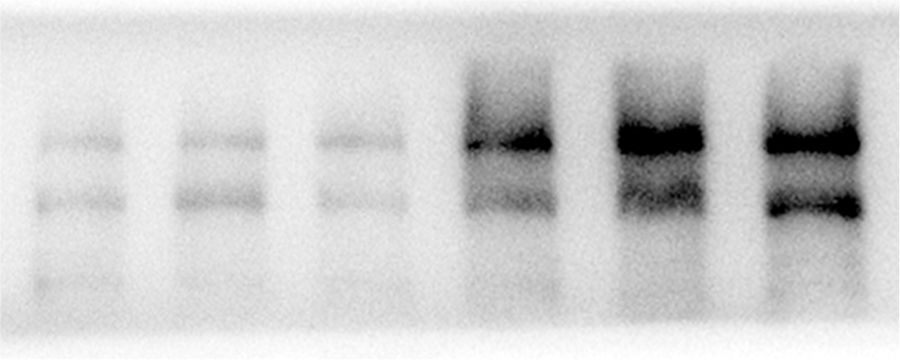

Supplement: Figure 3—source data 2. [file elife-97854-fig3-data2.zip › pmTOR-3R.tif]

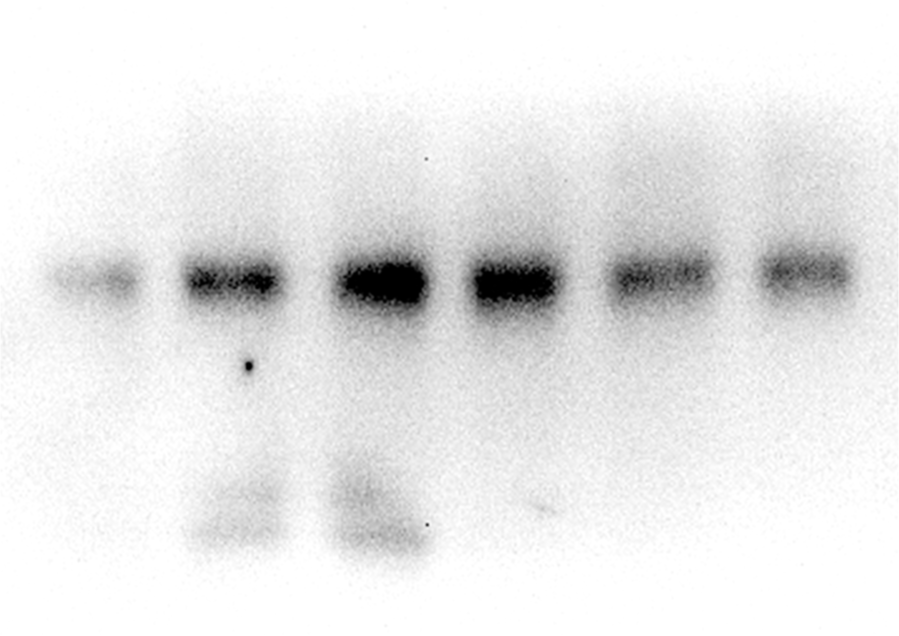

Supplement: Figure 3—source data 2. [file elife-97854-fig3-data2.zip › Proglucagen-3E.tif]

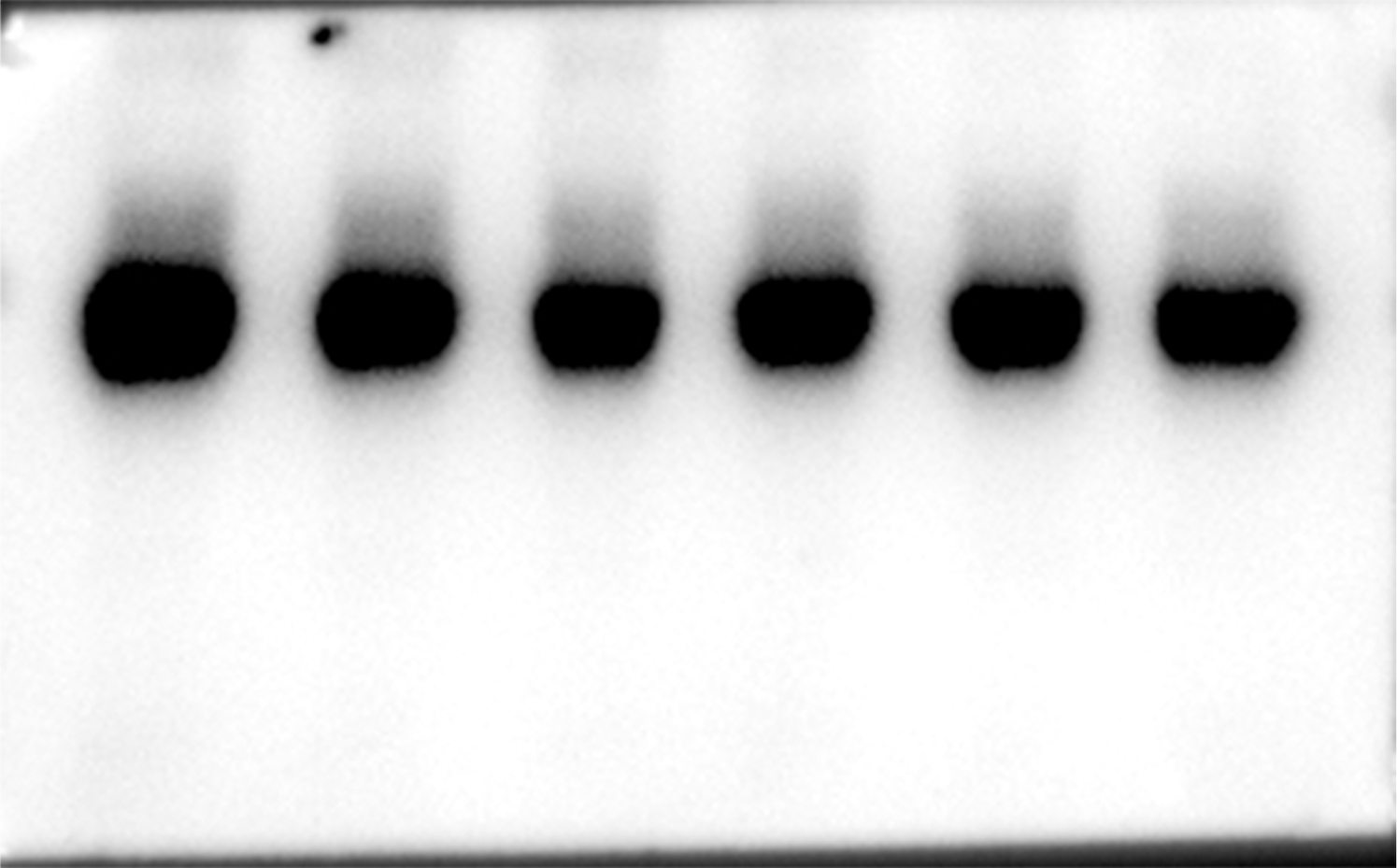

Supplement: Figure 3—source data 2. [file elife-97854-fig3-data2.zip › Proglucagen-3J.tif]

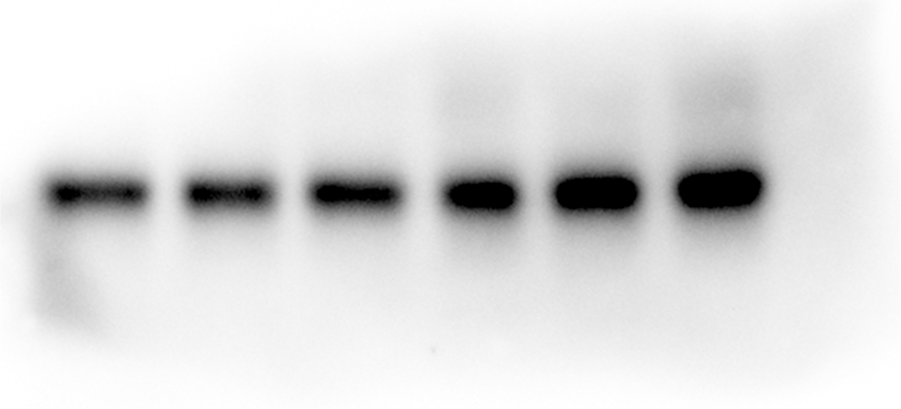

Supplement: Figure 3—source data 2. [file elife-97854-fig3-data2.zip › Proglucagen-3R.tif]

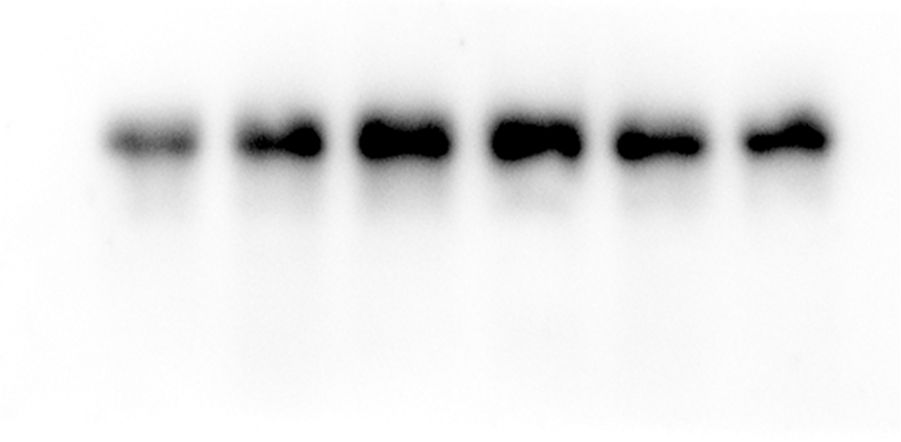

Supplement: Figure 3—source data 2. [file elife-97854-fig3-data2.zip › pS6-3E.tif]

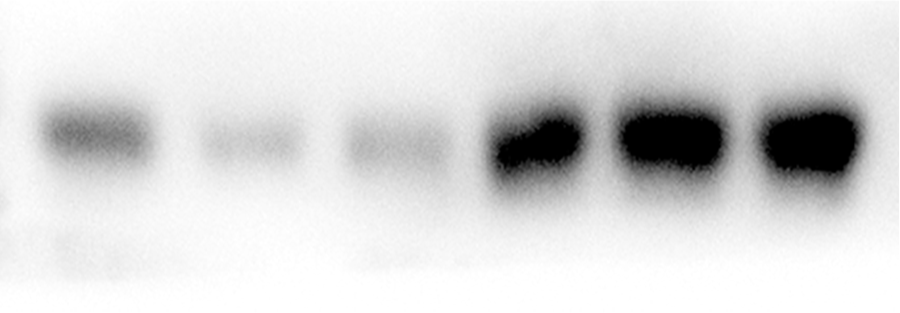

Supplement: Figure 3—source data 2. [file elife-97854-fig3-data2.zip › pS6-3R.tif]

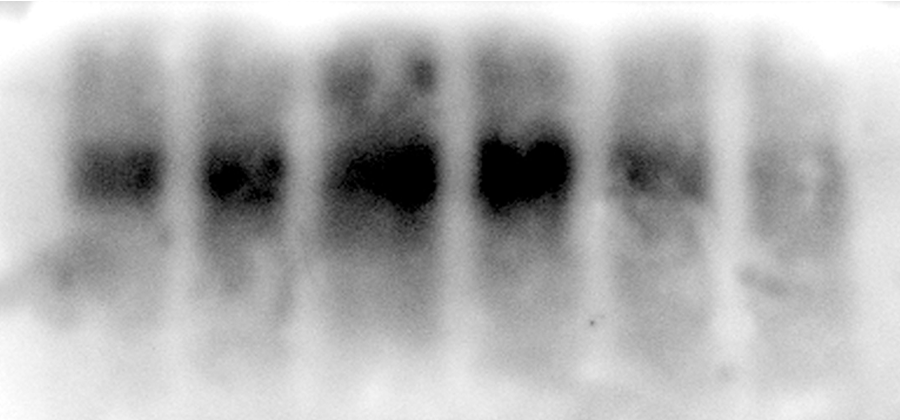

Supplement: Figure 3—source data 2. [file elife-97854-fig3-data2.zip › pS6K-3E.tif]

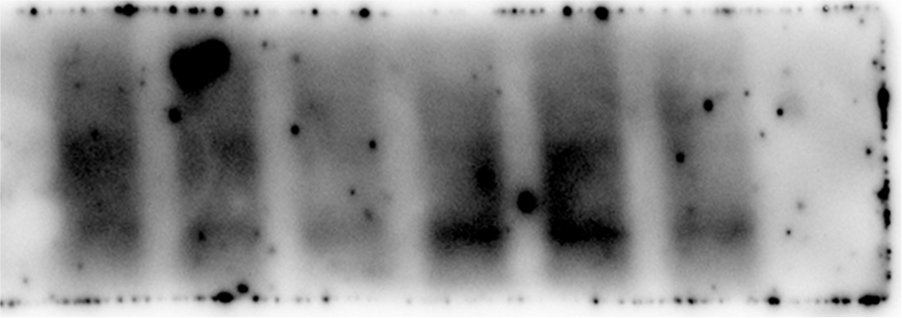

Supplement: Figure 3—source data 2. [file elife-97854-fig3-data2.zip › pS6K-3R.tif]

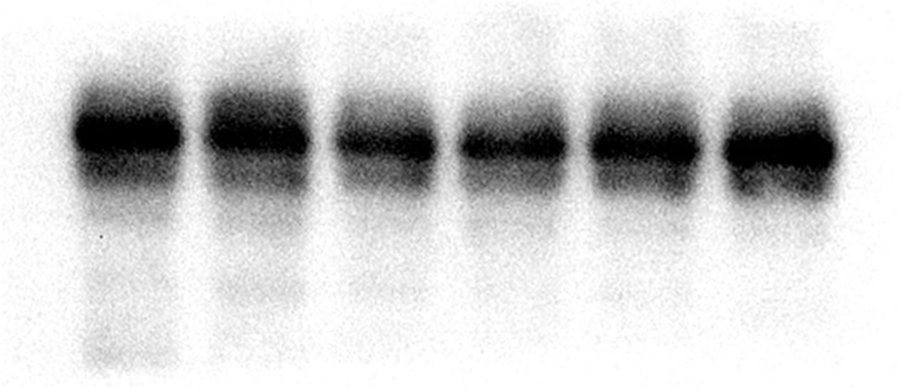

Supplement: Figure 3—source data 2. [file elife-97854-fig3-data2.zip › S6-3E.tif]

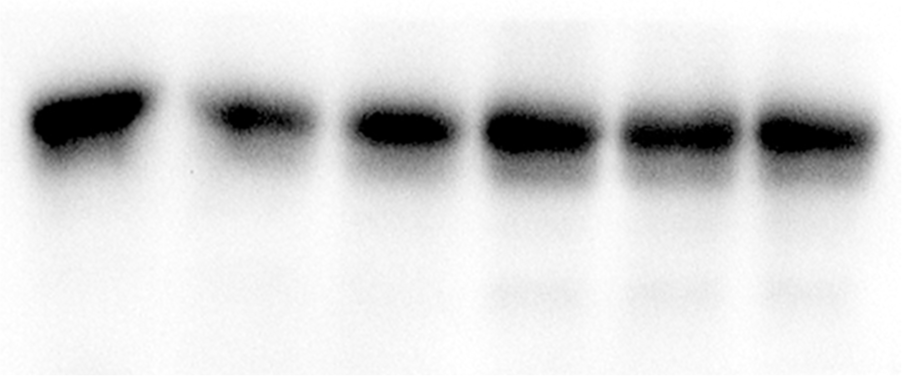

Supplement: Figure 3—source data 2. [file elife-97854-fig3-data2.zip › S6-3R.tif]

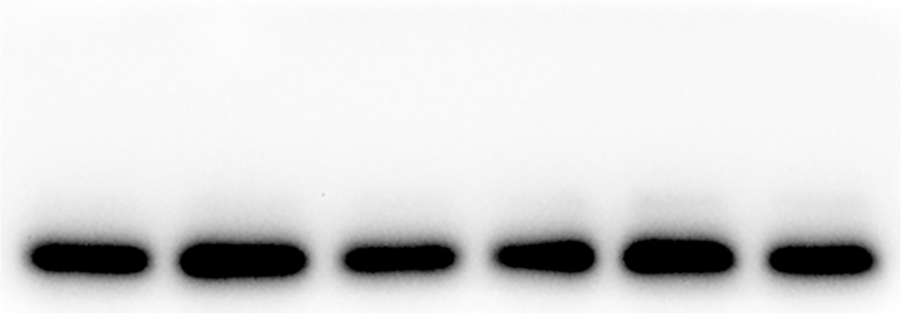

Supplement: Figure 3—source data 2. [file elife-97854-fig3-data2.zip › S6K-3E.tif]

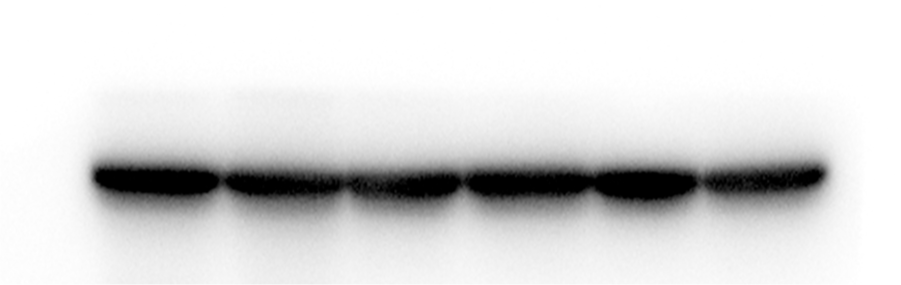

Supplement: Figure 3—source data 2. [file elife-97854-fig3-data2.zip › ╬▓-actin-3E.tif]

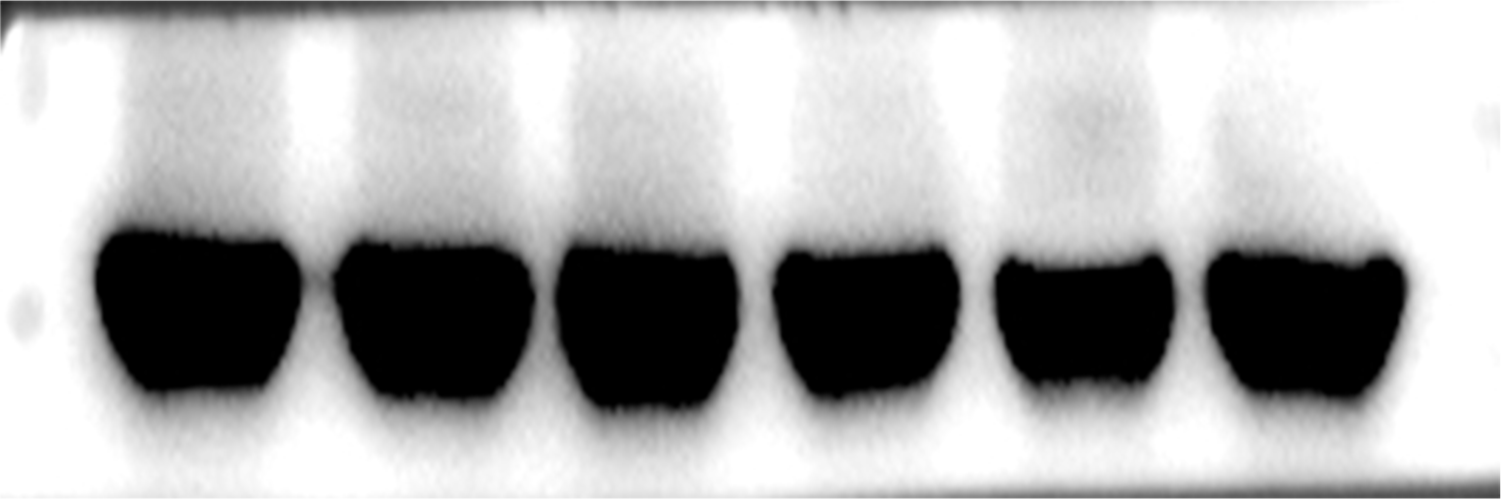

Supplement: Figure 3—source data 2. [file elife-97854-fig3-data2.zip › ╬▓-actin-3J.tif]

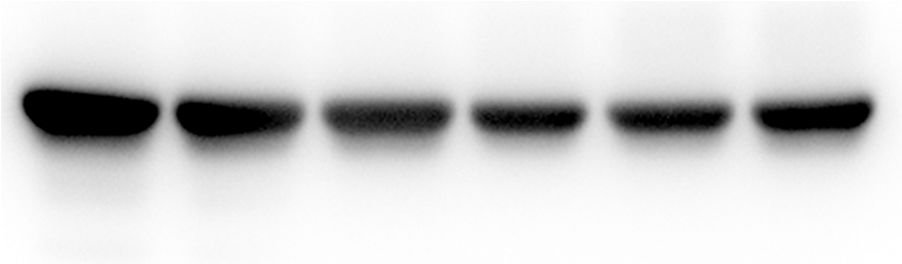

Supplement: Figure 3—source data 2. [file elife-97854-fig3-data2.zip › ╬▓-actin-3R.tif]

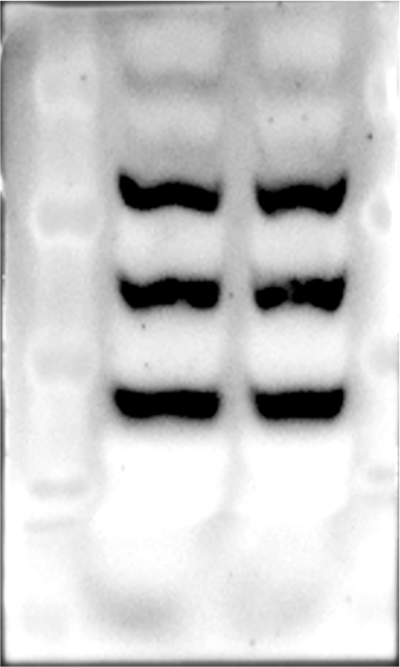

Supplement: Figure 4—source data 2. [file elife-97854-fig4-data2.zip › CaMKIV-4F.tif]

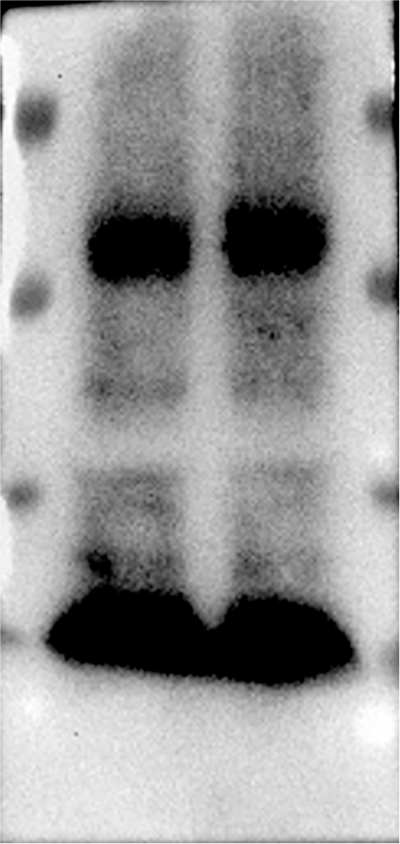

Supplement: Figure 4—source data 2. [file elife-97854-fig4-data2.zip › CaMKIV-4J.tif]

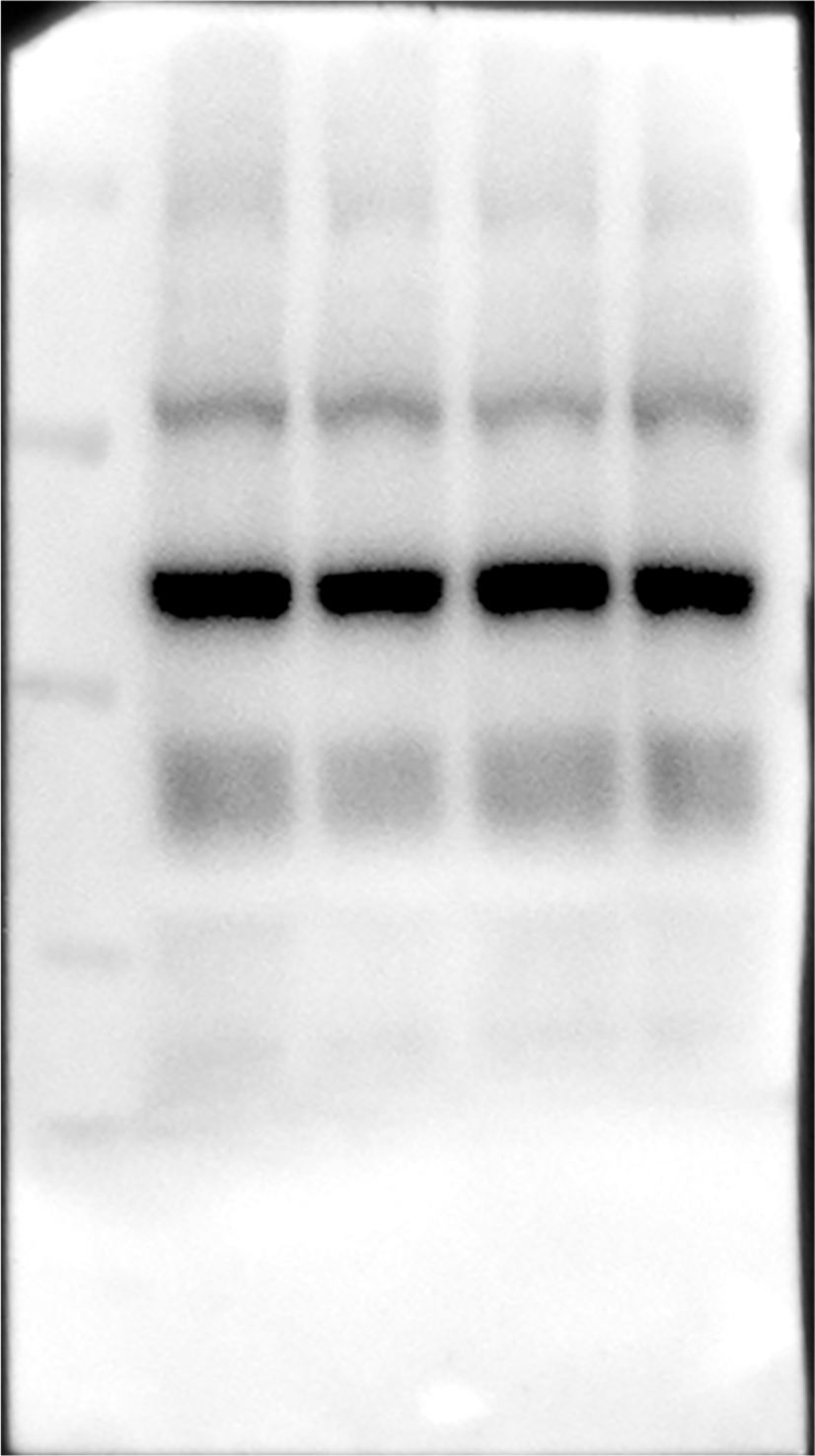

Supplement: Figure 4—source data 2. [file elife-97854-fig4-data2.zip › CaMKIV-4N.tif]

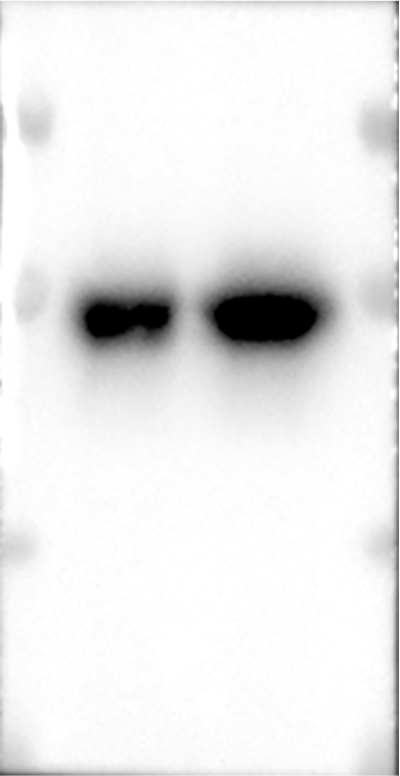

Supplement: Figure 4—source data 2. [file elife-97854-fig4-data2.zip › CaMKK╬▓-4F.tif]

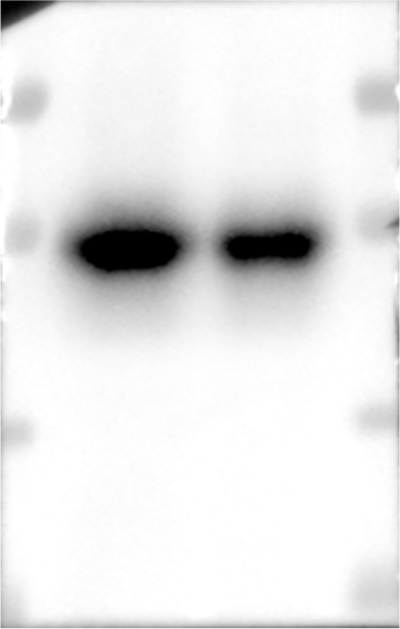

Supplement: Figure 4—source data 2. [file elife-97854-fig4-data2.zip › CaMKK╬▓-4J.tif]

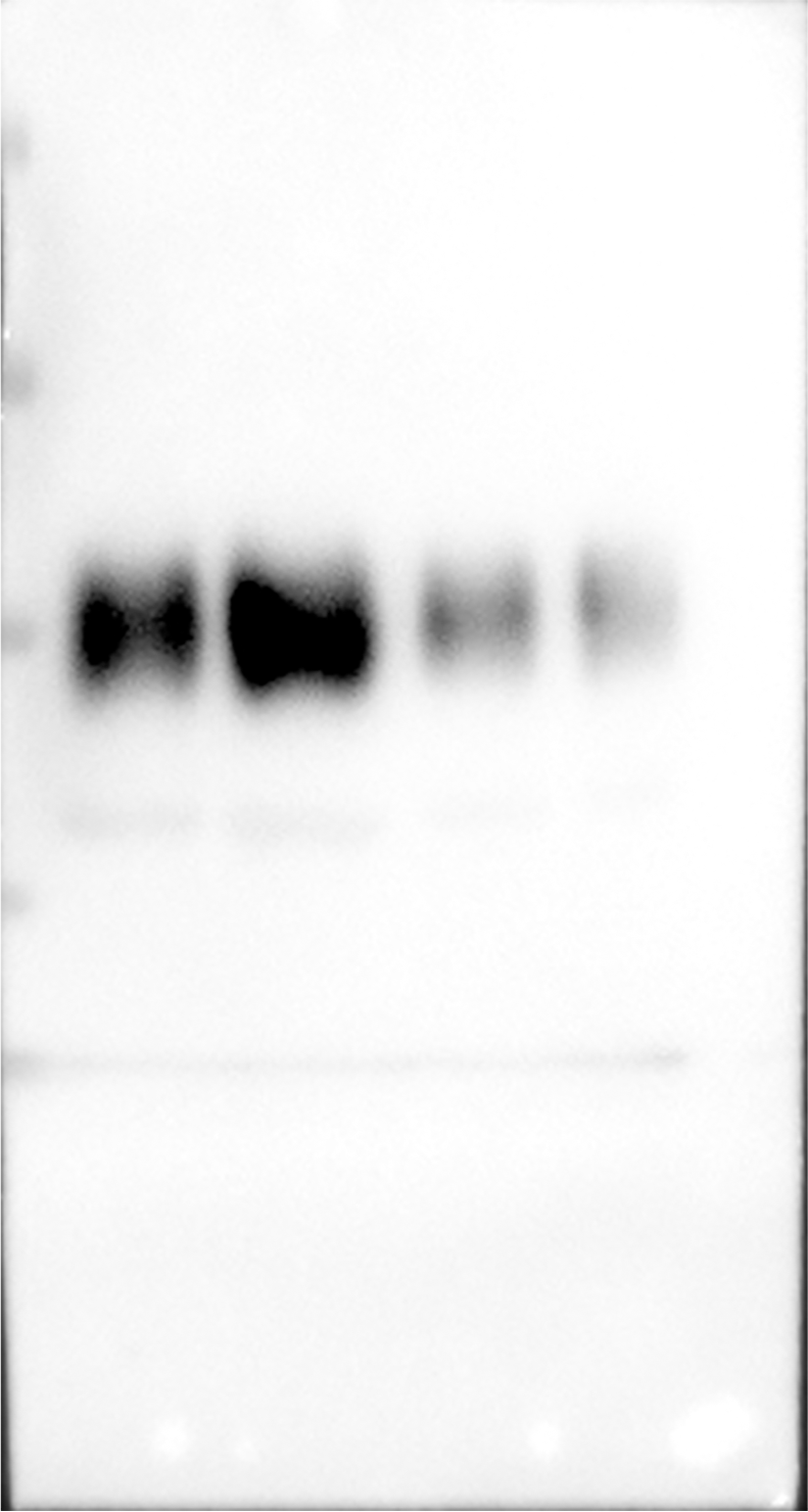

Supplement: Figure 4—source data 2. [file elife-97854-fig4-data2.zip › CaMKK╬▓-4N.tif]

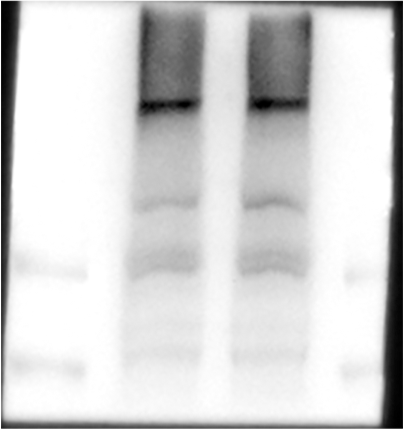

Supplement: Figure 4—source data 2. [file elife-97854-fig4-data2.zip › mTOR-4F.tif]

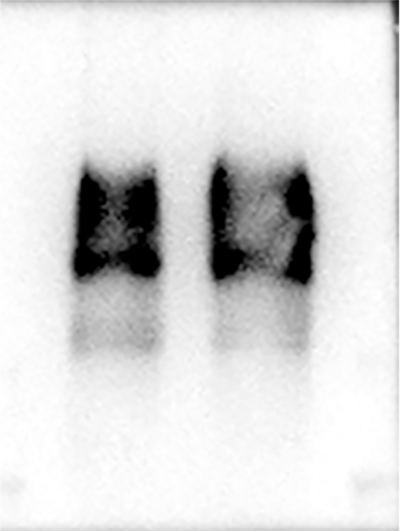

Supplement: Figure 4—source data 2. [file elife-97854-fig4-data2.zip › mTOR-4J.tif]

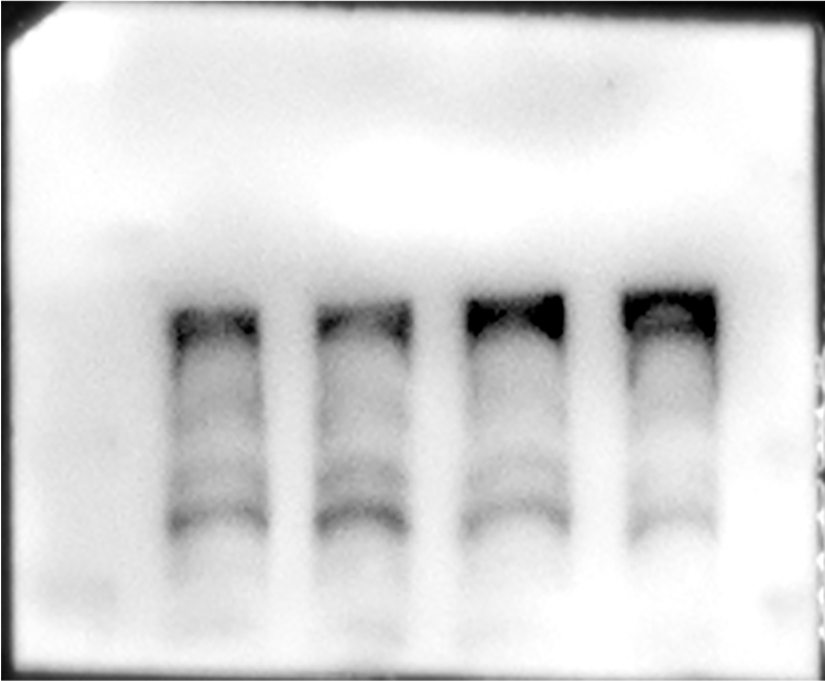

Supplement: Figure 4—source data 2. [file elife-97854-fig4-data2.zip › mTOR-4N.tif]

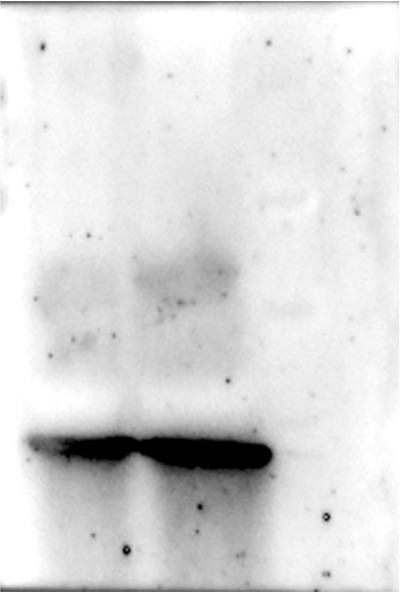

Supplement: Figure 4—source data 2. [file elife-97854-fig4-data2.zip › pCaMKIV-4F.tif]

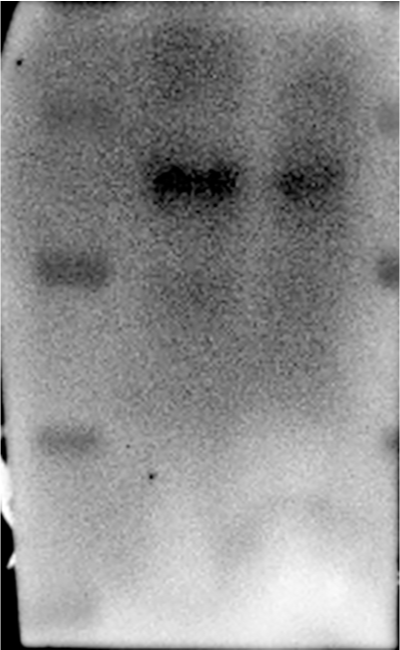

Supplement: Figure 4—source data 2. [file elife-97854-fig4-data2.zip › pCaMKIV-4J.tif]

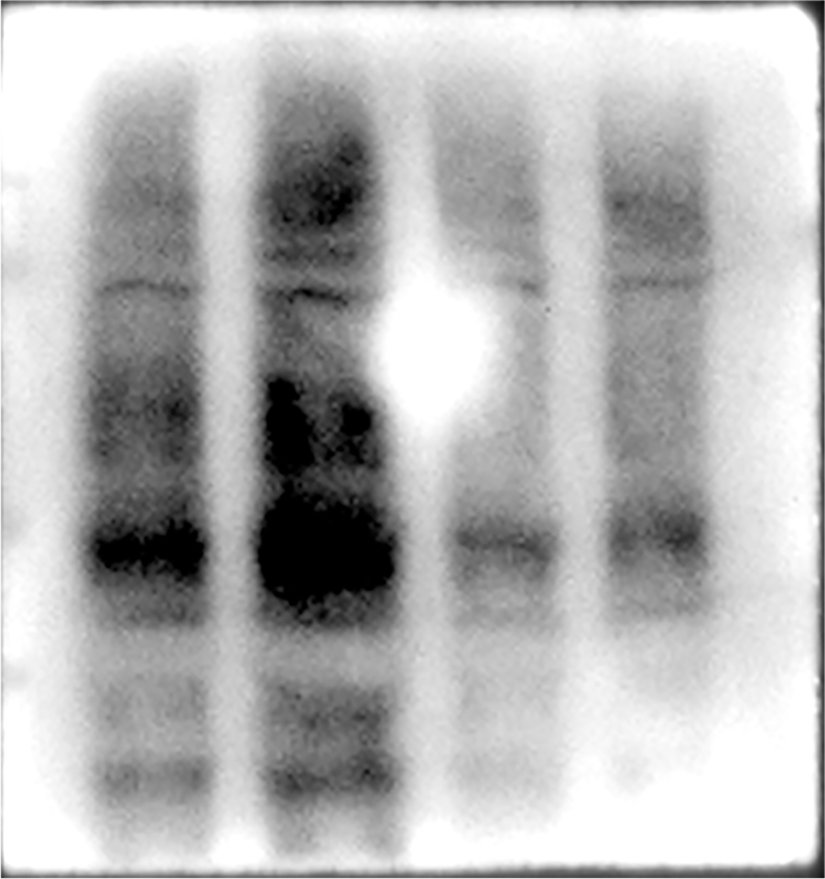

Supplement: Figure 4—source data 2. [file elife-97854-fig4-data2.zip › pCaMKIV-4N.tif]

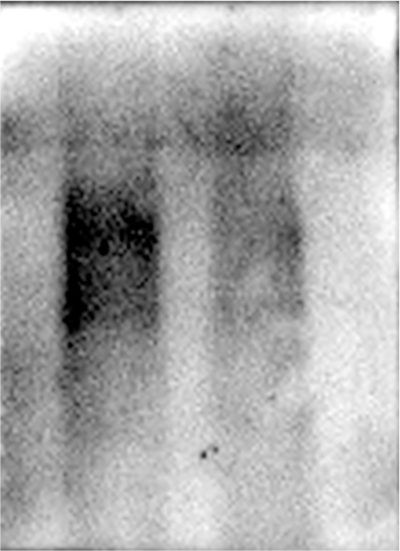

Supplement: Figure 4—source data 2. [file elife-97854-fig4-data2.zip › Piezo1-4J.tif]

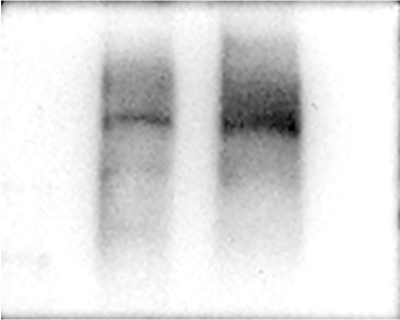

Supplement: Figure 4—source data 2. [file elife-97854-fig4-data2.zip › pmTOR-4F.tif]

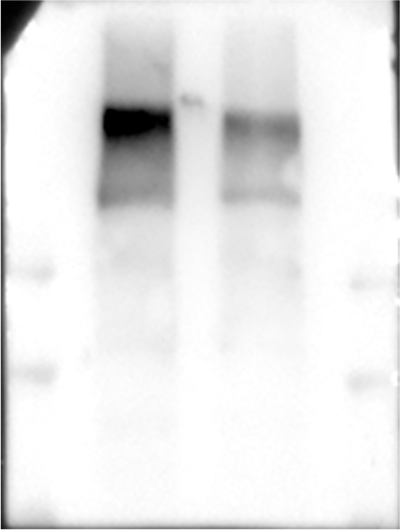

Supplement: Figure 4—source data 2. [file elife-97854-fig4-data2.zip › pmTOR-4J.tif]

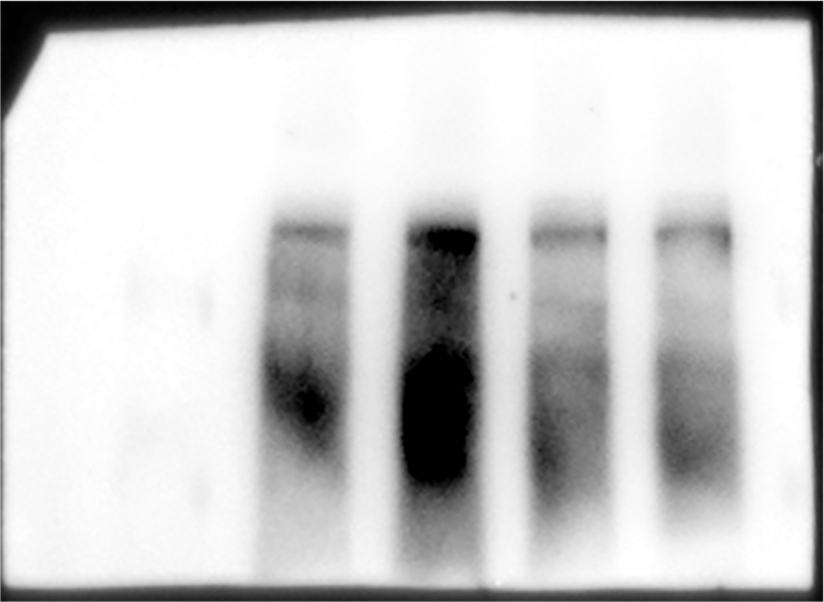

Supplement: Figure 4—source data 2. [file elife-97854-fig4-data2.zip › pmTOR-4N.tif]

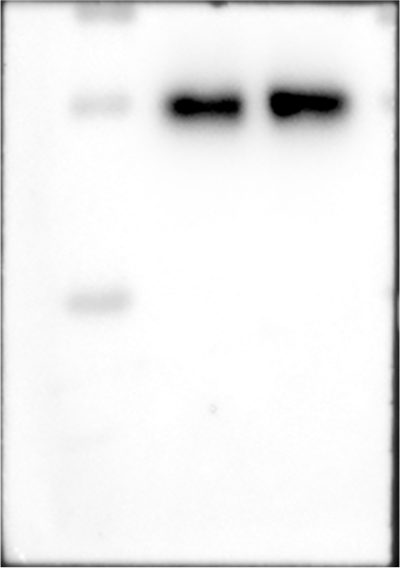

Supplement: Figure 4—source data 2. [file elife-97854-fig4-data2.zip › Proglucagen-4F.tif]

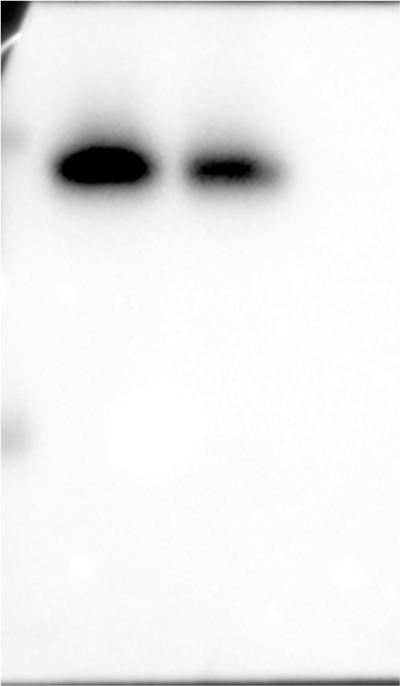

Supplement: Figure 4—source data 2. [file elife-97854-fig4-data2.zip › Proglucagen-4J.tif]

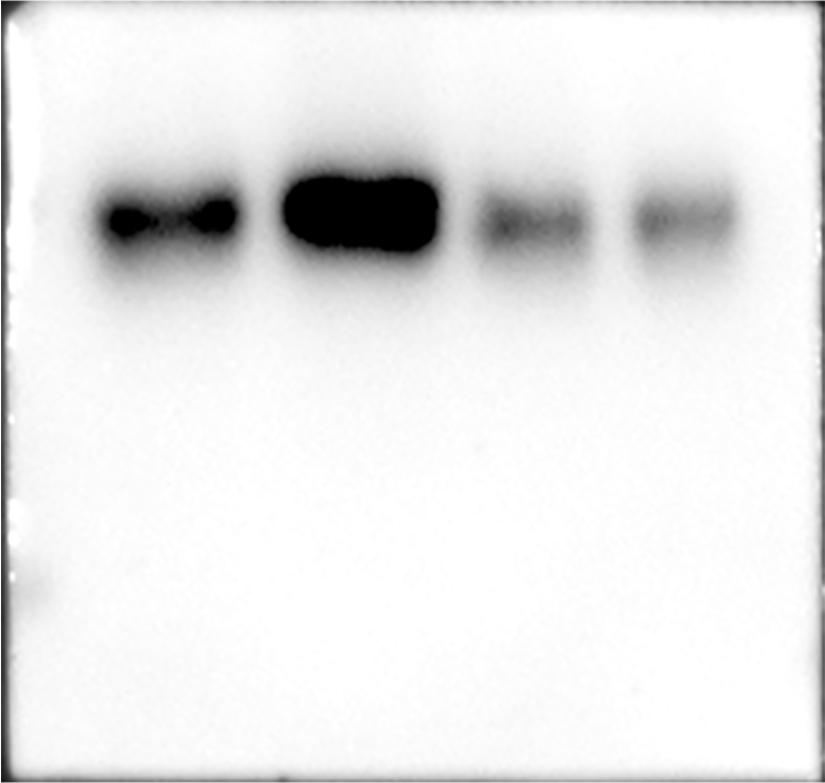

Supplement: Figure 4—source data 2. [file elife-97854-fig4-data2.zip › Proglucagen-4N.tif]

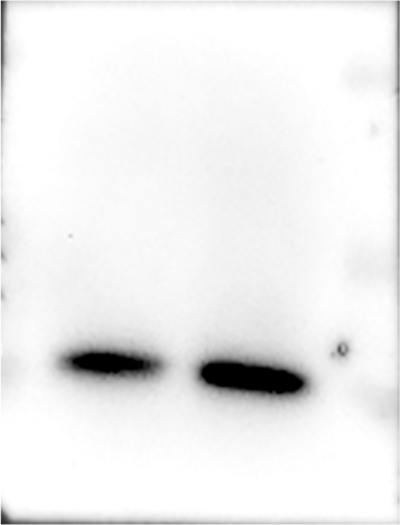

Supplement: Figure 4—source data 2. [file elife-97854-fig4-data2.zip › pS6-4F.tif]

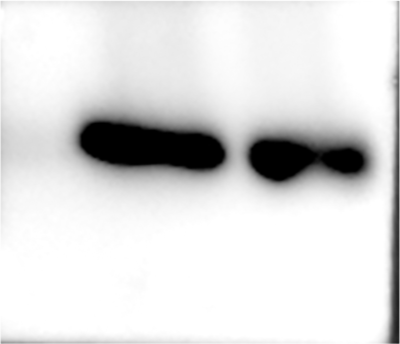

Supplement: Figure 4—source data 2. [file elife-97854-fig4-data2.zip › pS6-4J.tif]

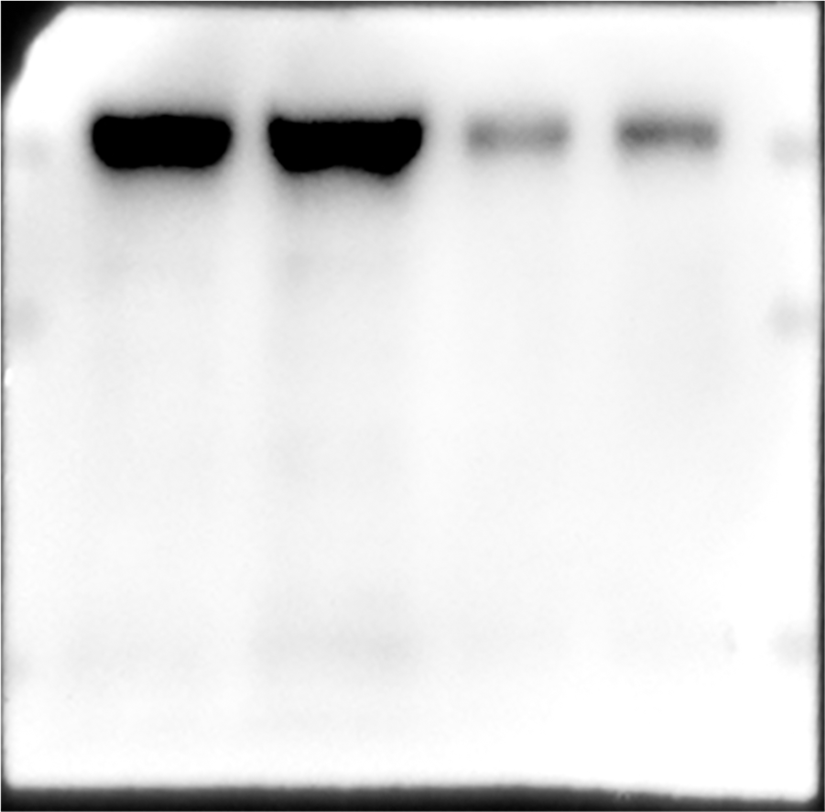

Supplement: Figure 4—source data 2. [file elife-97854-fig4-data2.zip › pS6-4N.tif]

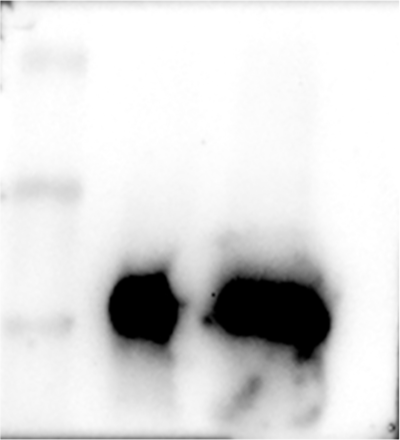

Supplement: Figure 4—source data 2. [file elife-97854-fig4-data2.zip › pS6K-4F.tif]

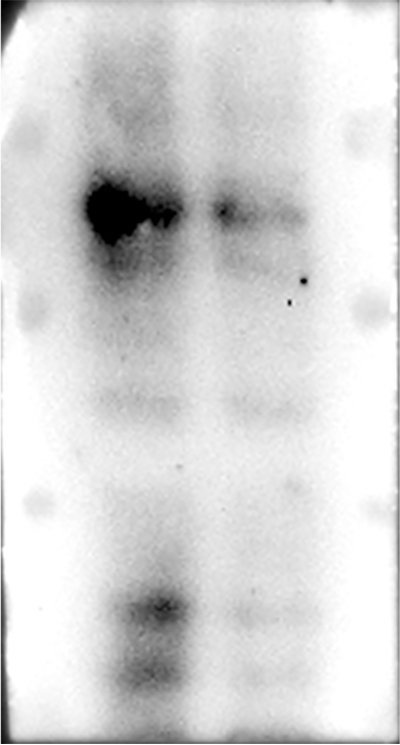

Supplement: Figure 4—source data 2. [file elife-97854-fig4-data2.zip › pS6K-4J.tif]

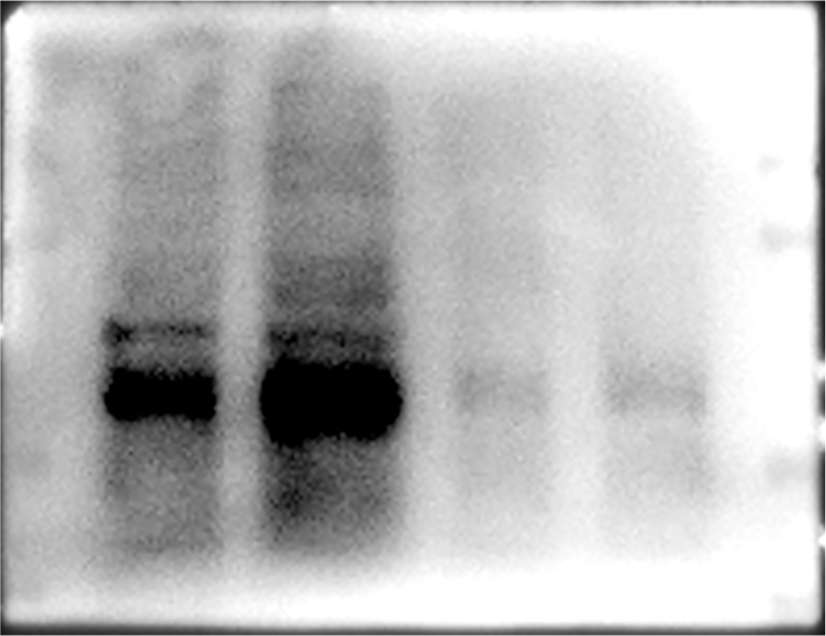

Supplement: Figure 4—source data 2. [file elife-97854-fig4-data2.zip › pS6K-4N.tif]

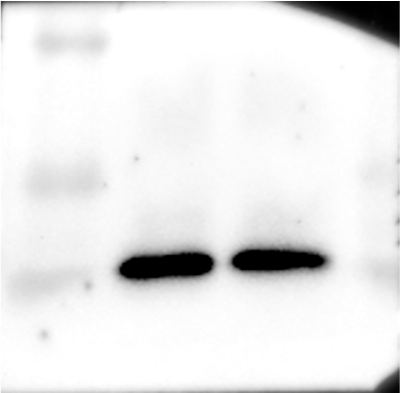

Supplement: Figure 4—source data 2. [file elife-97854-fig4-data2.zip › S6-4F.tif]

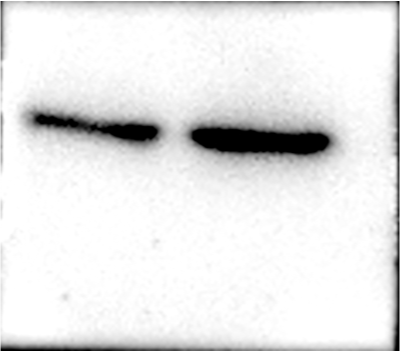

Supplement: Figure 4—source data 2. [file elife-97854-fig4-data2.zip › S6-4J.tif]

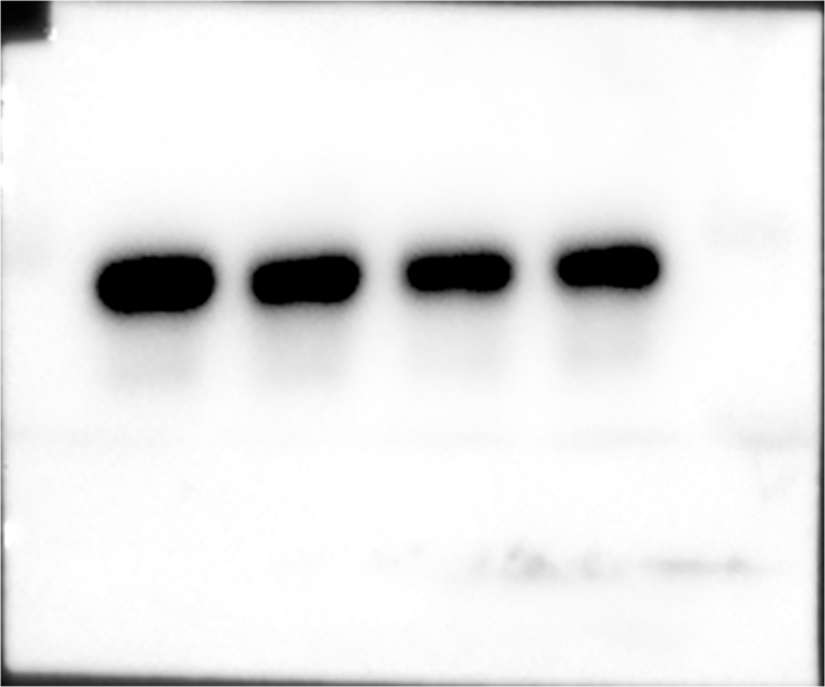

Supplement: Figure 4—source data 2. [file elife-97854-fig4-data2.zip › S6-4N.tif]

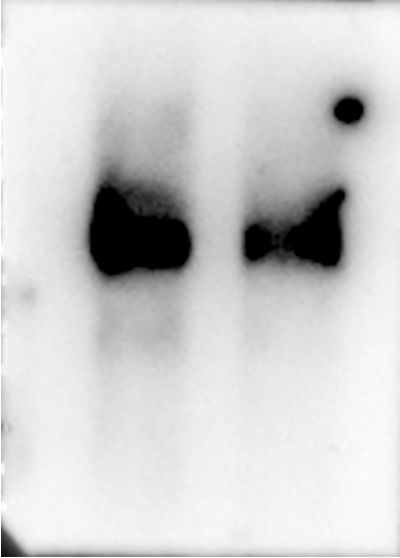

Supplement: Figure 4—source data 2. [file elife-97854-fig4-data2.zip › S6K-4F.tif]

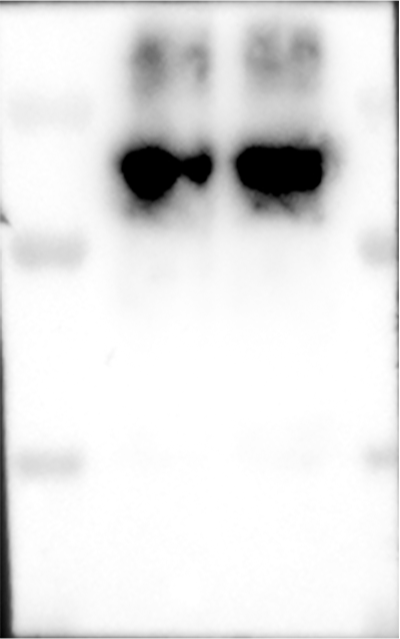

Supplement: Figure 4—source data 2. [file elife-97854-fig4-data2.zip › S6K-4J.tif]

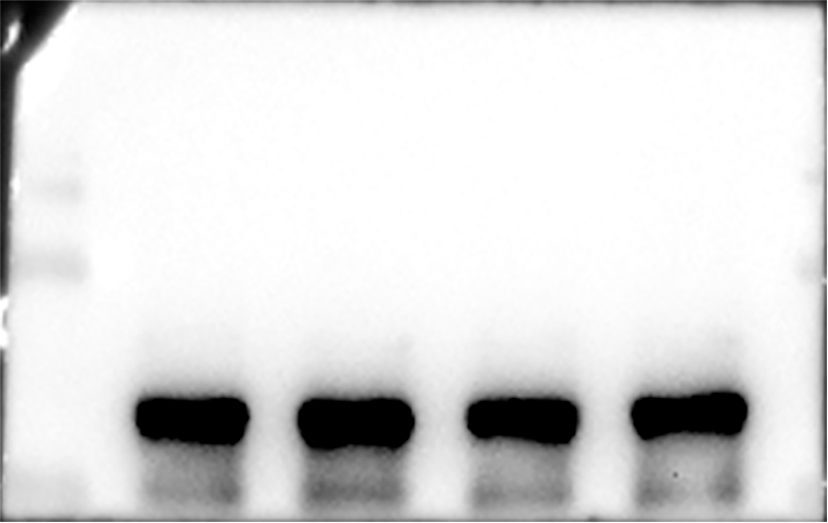

Supplement: Figure 4—source data 2. [file elife-97854-fig4-data2.zip › S6K-4N.tif]

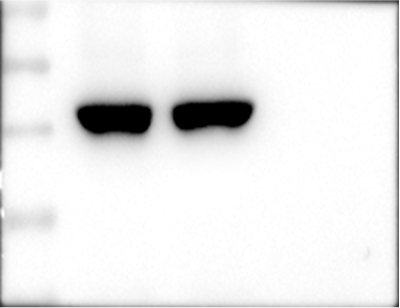

Supplement: Figure 4—source data 2. [file elife-97854-fig4-data2.zip › ╬▓-actin-4F.tif]

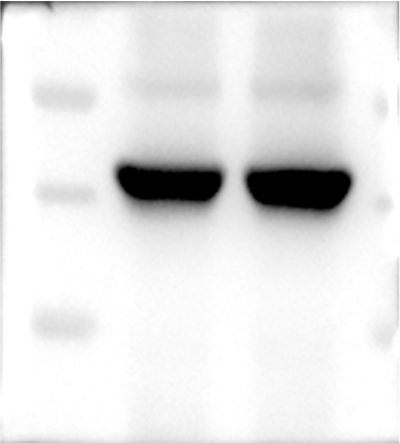

Supplement: Figure 4—source data 2. [file elife-97854-fig4-data2.zip › ╬▓-actin-4J.tif]

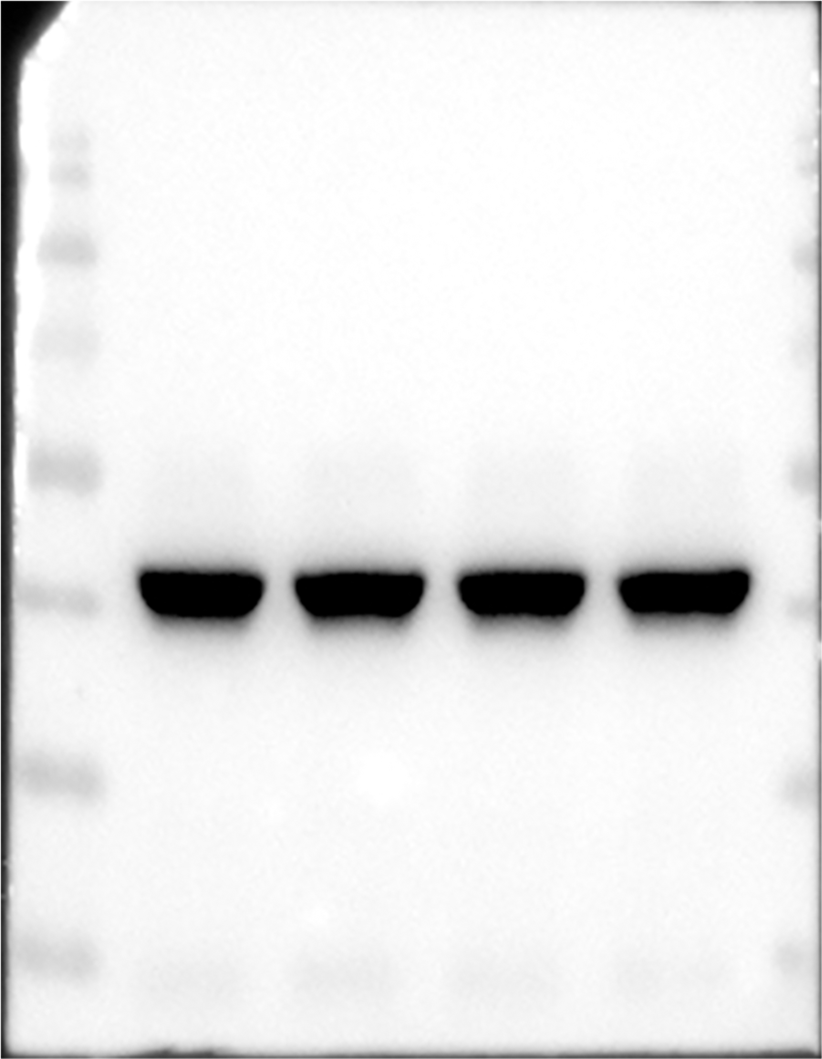

Supplement: Figure 4—source data 2. [file elife-97854-fig4-data2.zip › ╬▓-actin-4N.tif]

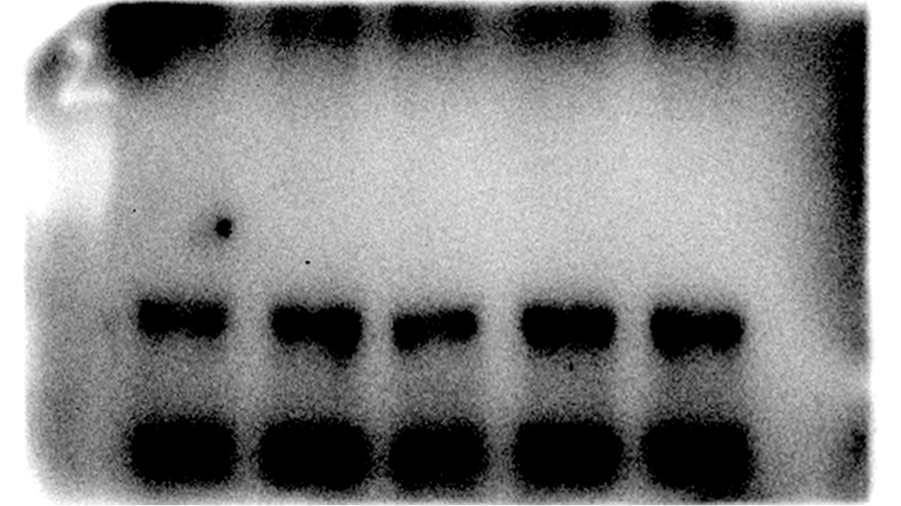

Supplement: Figure 5—source data 2. [file elife-97854-fig5-data2.zip › CaMKIV-5D.tif]

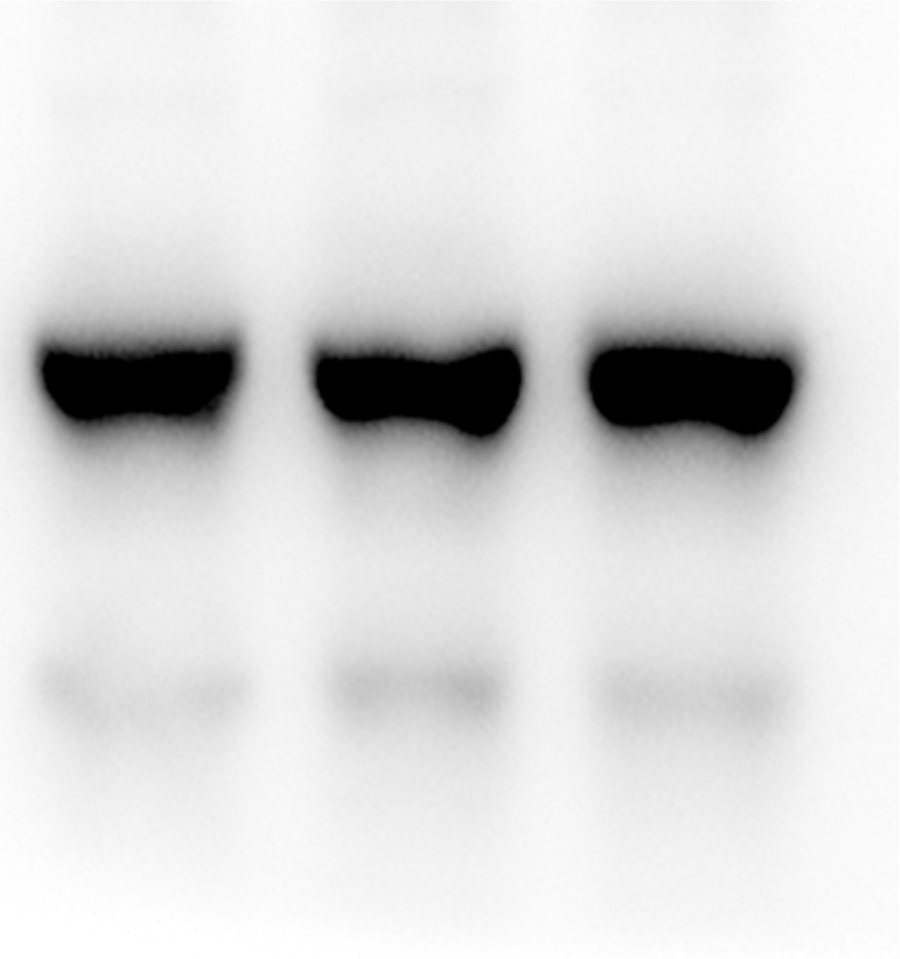

Supplement: Figure 5—source data 2. [file elife-97854-fig5-data2.zip › CaMKIV-5G.tif]

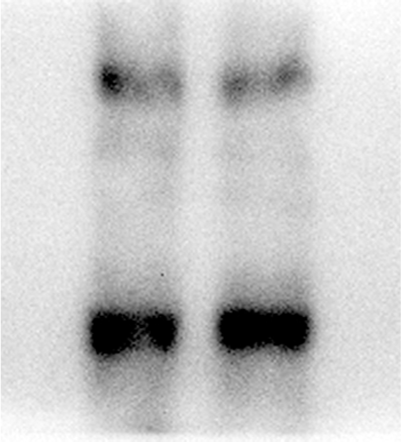

Supplement: Figure 5—source data 2. [file elife-97854-fig5-data2.zip › CaMKIV-5N.tif]

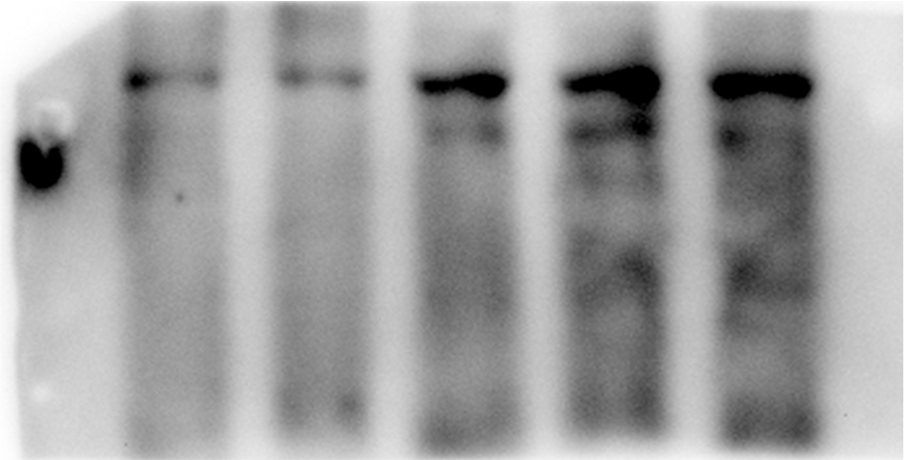

Supplement: Figure 5—source data 2. [file elife-97854-fig5-data2.zip › CaMKK╬▓-5D.tif]

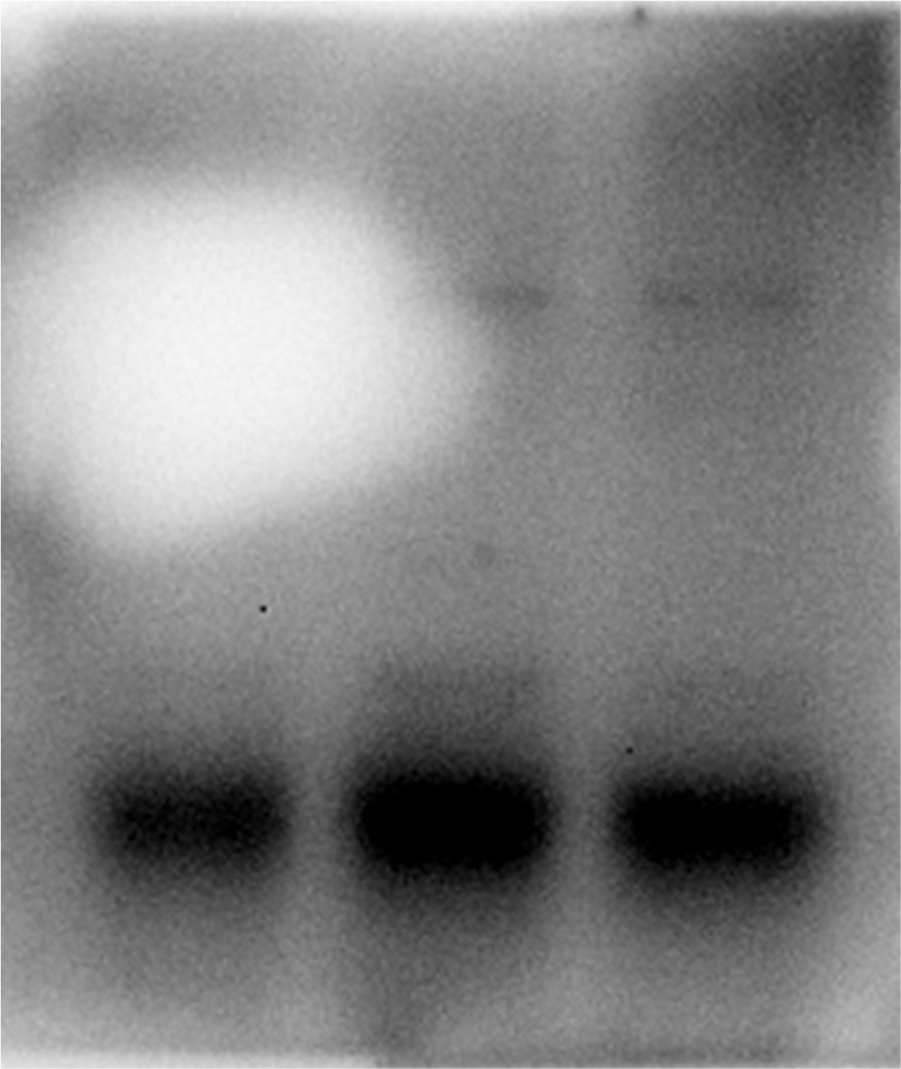

Supplement: Figure 5—source data 2. [file elife-97854-fig5-data2.zip › CaMKK╬▓-5G.tif]

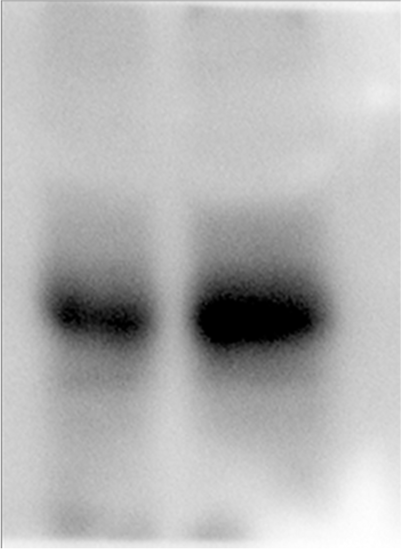

Supplement: Figure 5—source data 2. [file elife-97854-fig5-data2.zip › CaMKK╬▓-5N.tif]

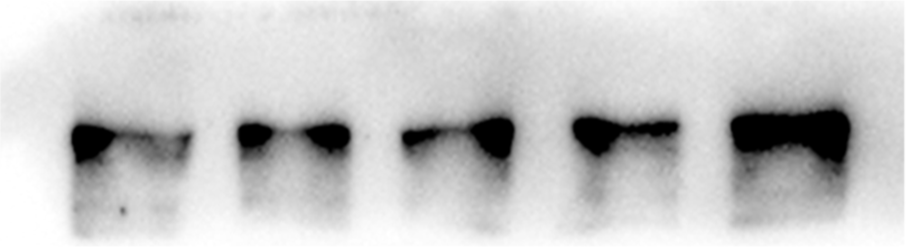

Supplement: Figure 5—source data 2. [file elife-97854-fig5-data2.zip › mTOR-5D.tif]
