# Supplementary material for: Mechano-regulation of GLP-1 production by Piezo1 in intestinal L cells
Source: eLife. 2024 Nov 7;13:RP97854. doi: 10.7554/eLife.97854 (PMC11542922; doi:10.7554/eLife.97854)
Supplement: Figure 1—figure supplement 1—source data 1. [file elife-97854-fig1-figsupp1-data1.zip › Figure1-figure supplement 1-source data 1.pdf]

Figure 1—figure supplement 1E

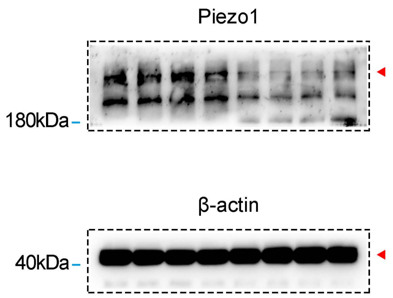

Figure 1, Figure supplement 1, Source Data 1. Original membranes corresponding to Figure Supplement 2. C57BL/6J mice were subjected to different feeding treatments: lanes 1, 2, and 3 represent normal diet feeding, while lanes 4, 5, and 6 correspond to high-fat feeding treatments.
