# Supplementary material for: Mechano-regulation of GLP-1 production by Piezo1 in intestinal L cells
Source: eLife. 2024 Nov 7;13:RP97854. doi: 10.7554/eLife.97854 (PMC11542922; doi:10.7554/eLife.97854)
Supplement: Figure 1—figure supplement 3—source data 1. [file elife-97854-fig1-figsupp3-data1.zip › Figure 1-figure supplement 3-source data 1.pdf]

Figure 1—figure supplement 3D

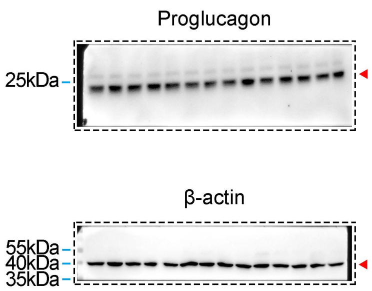

Figure 1, Figure supplement 3, Source Data 1. Original membranes corresponding to Figure supplement 3. Under normal diet feeding, *Piezo1*$^{loxp/loxp}$ mice were used in lane 1, 2, 3, *Piezo1* IntL-CKO mice in lane 4, 5, 6, *Vil1*^{Flp} mice in lane 7, 8, 9, *Gcg*^{cre} mice in lane 10, 11, 12, and *Vil1*^{FLP}*::Gcg*^{frtCre} mice in lane 13, 14.
