# Supplementary material for: Mechano-regulation of GLP-1 production by Piezo1 in intestinal L cells
Source: eLife. 2024 Nov 7;13:RP97854. doi: 10.7554/eLife.97854 (PMC11542922; doi:10.7554/eLife.97854)
Supplement: Figure 2—source data 1. [file elife-97854-fig2-data1.zip › Figure2-source data 1.pdf]

Figure 2F

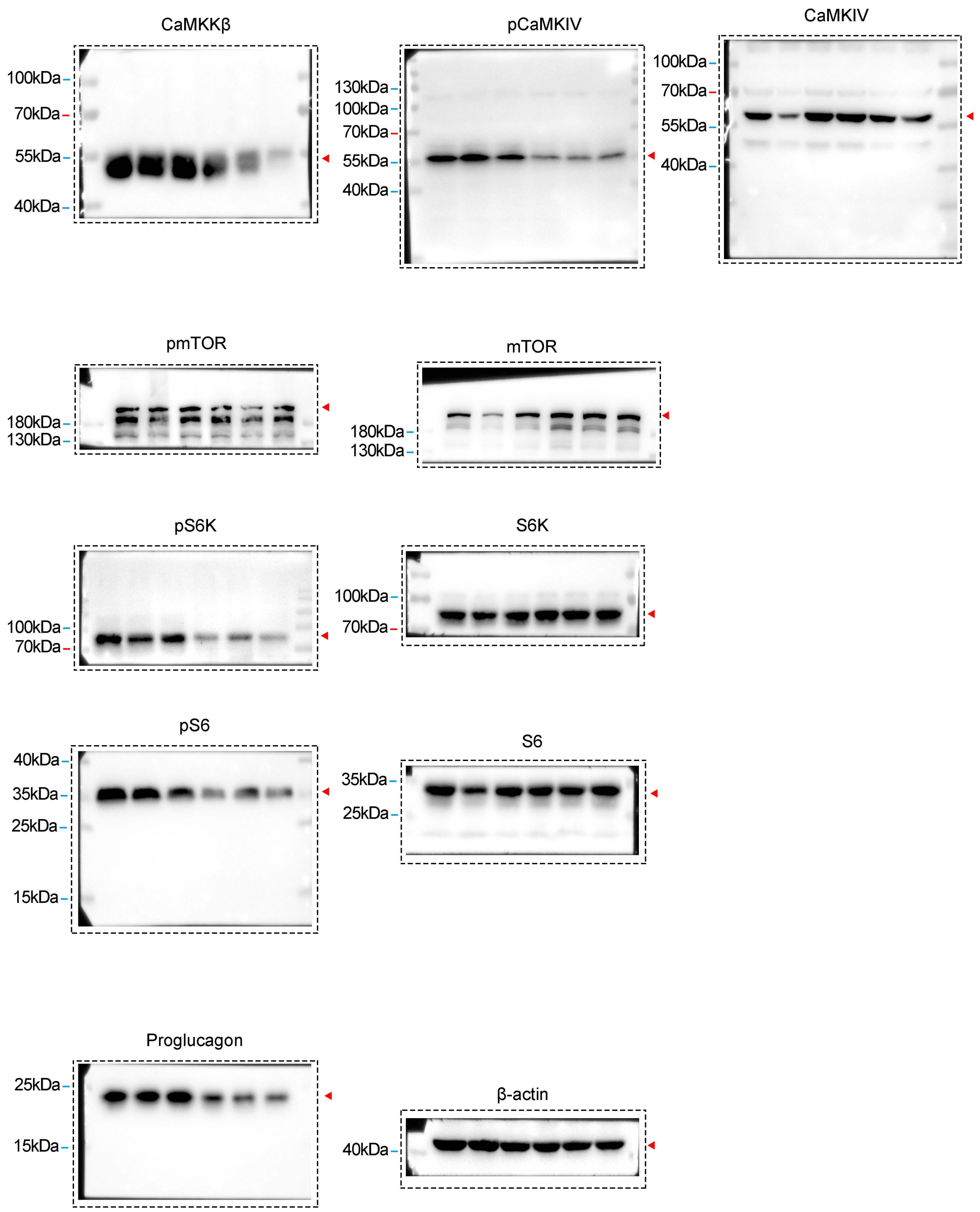

Figure 2, Source Data 1. Original membranes corresponding to Figure 2, panel F. Among them,1,2,3 lanes are *Piezo1*$^{loxp/loxp}$, and 4,5,6 lanes are *Piezo1* IntL-CKO.
