# Supplementary material for: Mechano-regulation of GLP-1 production by Piezo1 in intestinal L cells
Source: eLife. 2024 Nov 7;13:RP97854. doi: 10.7554/eLife.97854 (PMC11542922; doi:10.7554/eLife.97854)
Supplement: Figure 2—figure supplement 1—source data 1. [file elife-97854-fig2-figsupp1-data1.zip › Figure 2-figure supplement 1-source data 1.pdf]

Figure 2-figure supplement 9C

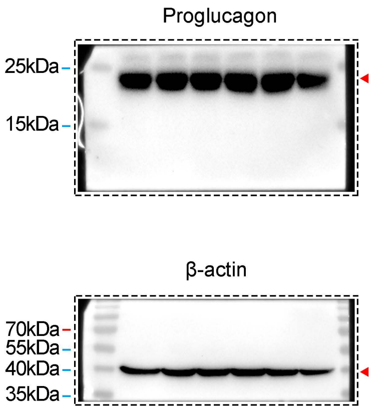

Figure 2, Figure supplement 9, Source Data 1. Original membranes corresponding to Figure supplement 3. On high-fat diet feeding, 1, 2, 3 lanes were *Piezo1*$^{loxp/loxp}$ mice, and 4, 5, 6 lanes were *Piezo1* IntL-CKO mice.
