# Supplementary material for: Mechano-regulation of GLP-1 production by Piezo1 in intestinal L cells
Source: eLife. 2024 Nov 7;13:RP97854. doi: 10.7554/eLife.97854 (PMC11542922; doi:10.7554/eLife.97854)
Supplement: Figure 3—source data 1. [file elife-97854-fig3-data1.zip › Figure3-source data 1.pdf]

Figure 3E

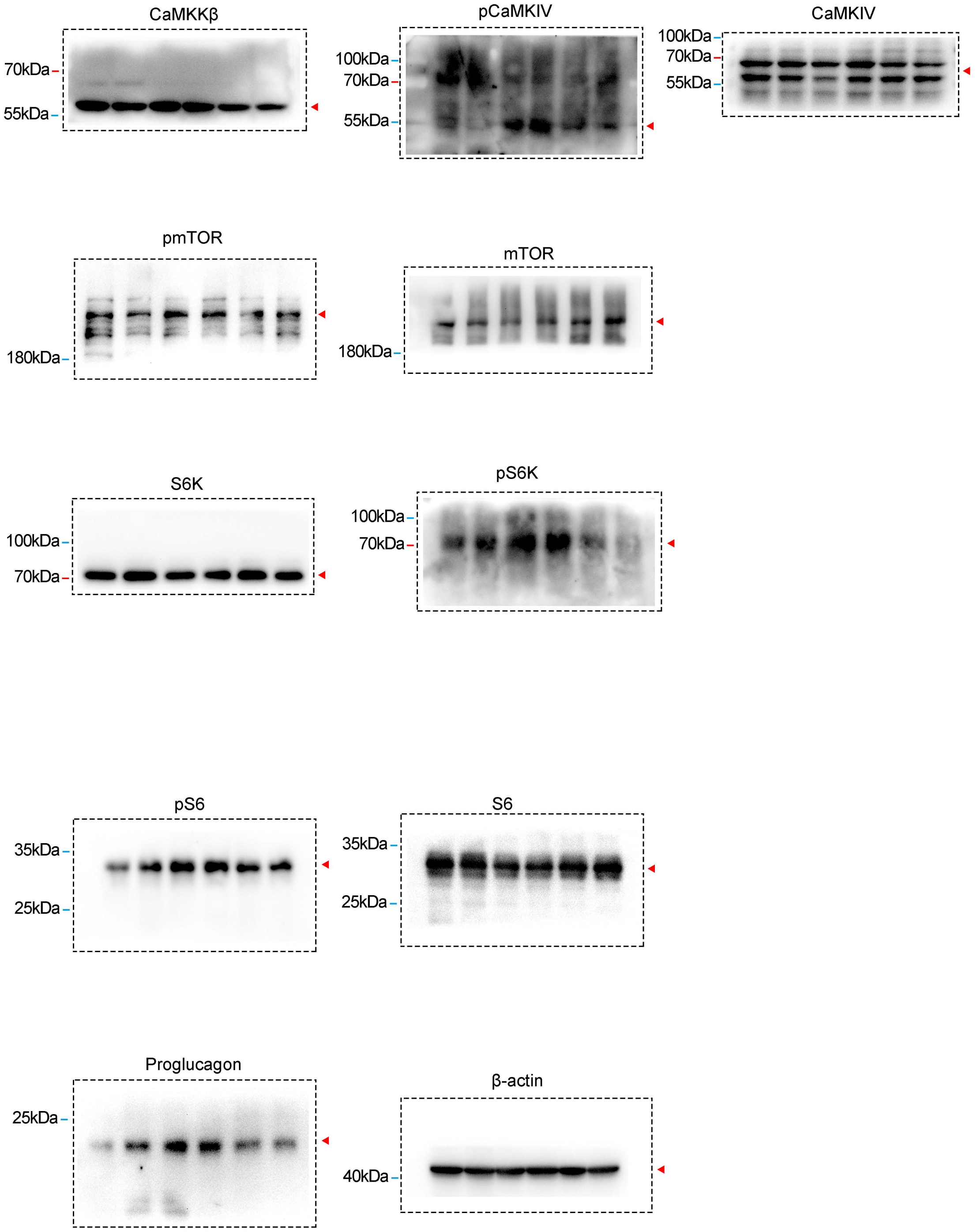

Figure 3, Source Data 1. Original membranes corresponding to Figure 3, panel E. Lanes 1 and 2 represent wild type (WT) samples, lanes 3 and 4 are WT samples that received intraperitoneal injections of Yoda1, while lanes 5 and 6 correspond to WT samples that received intraperitoneal injections of both GsMTx4 and Yoda1.

Figure 3J

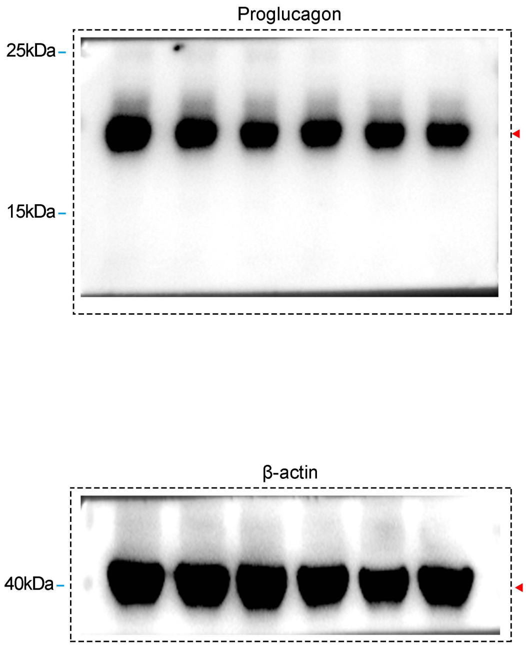

Figure 3, Source Data 1. The original membranes corresponding to Figure 3, panel J. Lanes 1, 2, and 3 represent intraperitoneal injections of vehicle solution in *Piezo1* IntL-CKO, while lanes 4, 5, and 6 correspond to intraperitoneal injections of Yoda1 in *Piezo1* IntL-CKO.

Figure 3R

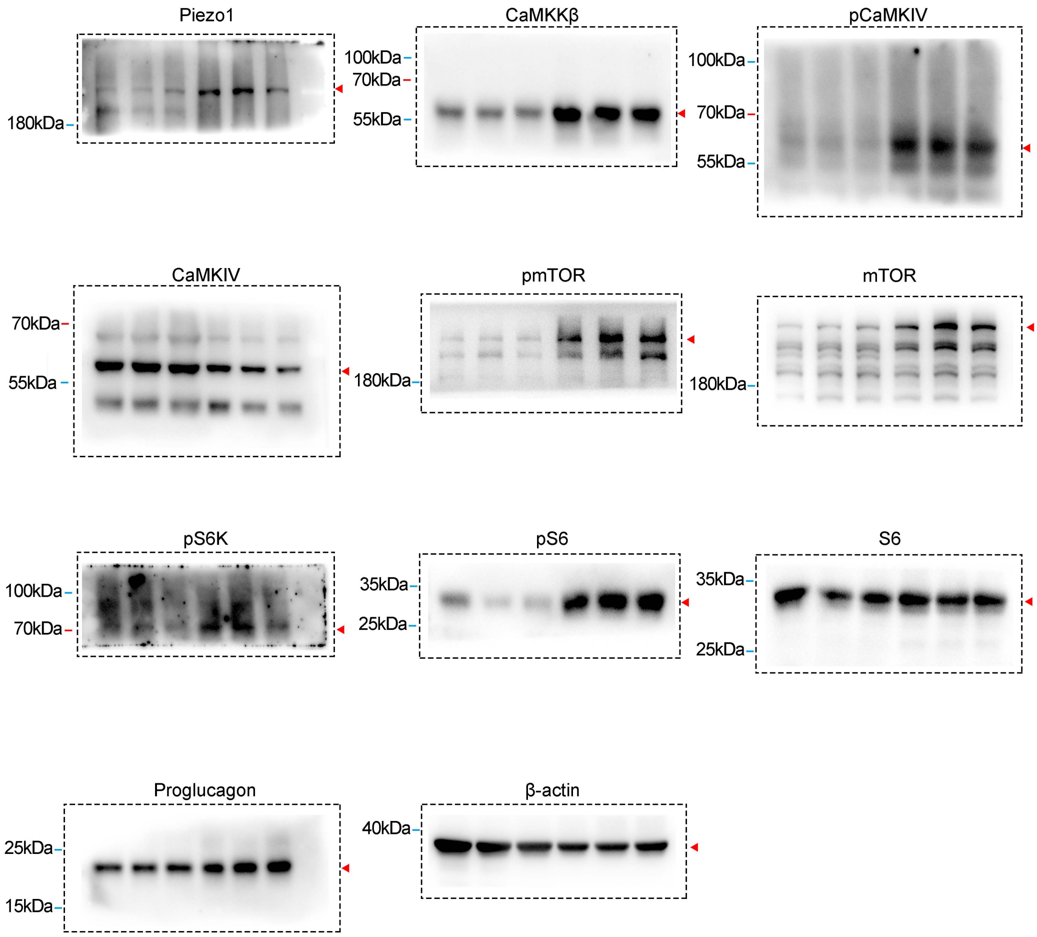

Figure 3, Source Data 1. Original membranes corresponding to Figure 3, panel R. Lanes 1, 2, and 3 represent high-fat-fed wild-type (WT) mice, while lanes 4, 5, and 6 were obtained following the surgical implantation of silicone beads into the ileum of high-fat-fed WT mice
