# Supplementary material for: Mechano-regulation of GLP-1 production by Piezo1 in intestinal L cells
Source: eLife. 2024 Nov 7;13:RP97854. doi: 10.7554/eLife.97854 (PMC11542922; doi:10.7554/eLife.97854)
Supplement: Figure 4—source data 1. [file elife-97854-fig4-data1.zip › Figure4-source data 1.pdf]

Figure 4F

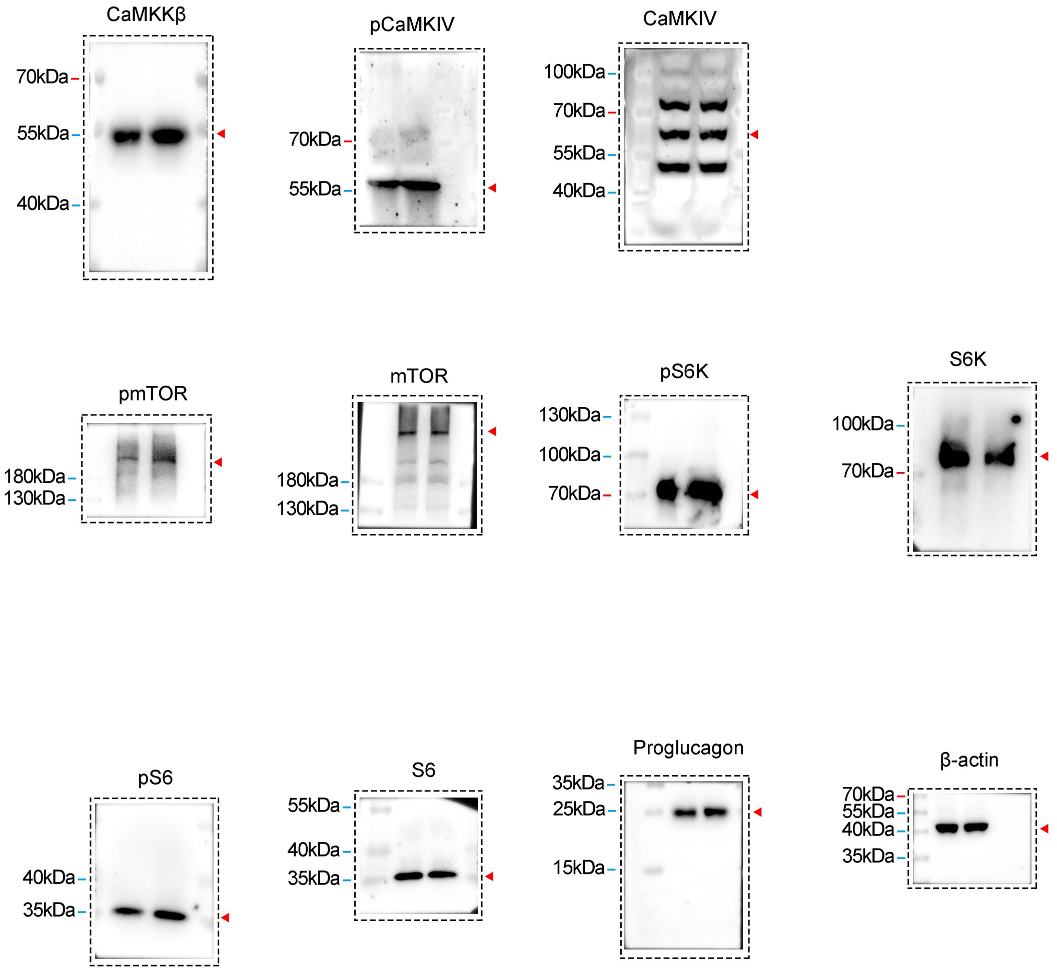

Figure 4, Source Data 1. The original membranes corresponding to Figure 4, panel F, are presented here. In the first lane, primary L cells were treated with DMSO, while the second lane features primary L cells treated with Yoda1.

Figure 4J

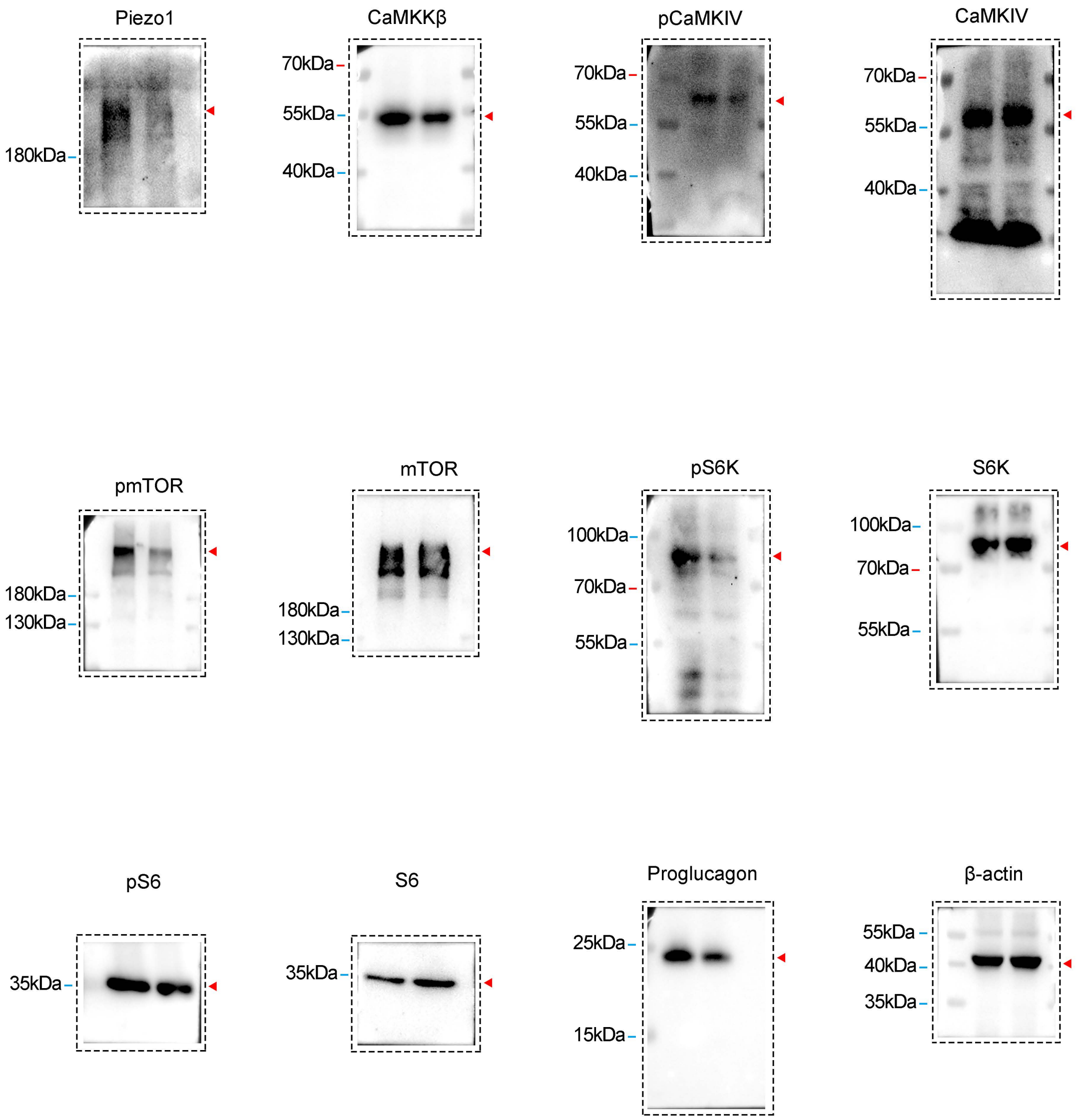

Figure 4, Source Data 1. Original membranes corresponding to Figure 4, panel J. The first lane represents control treatment, while the second lane features primary L cells treated with sh-*Piezo1*.

Figure 4N

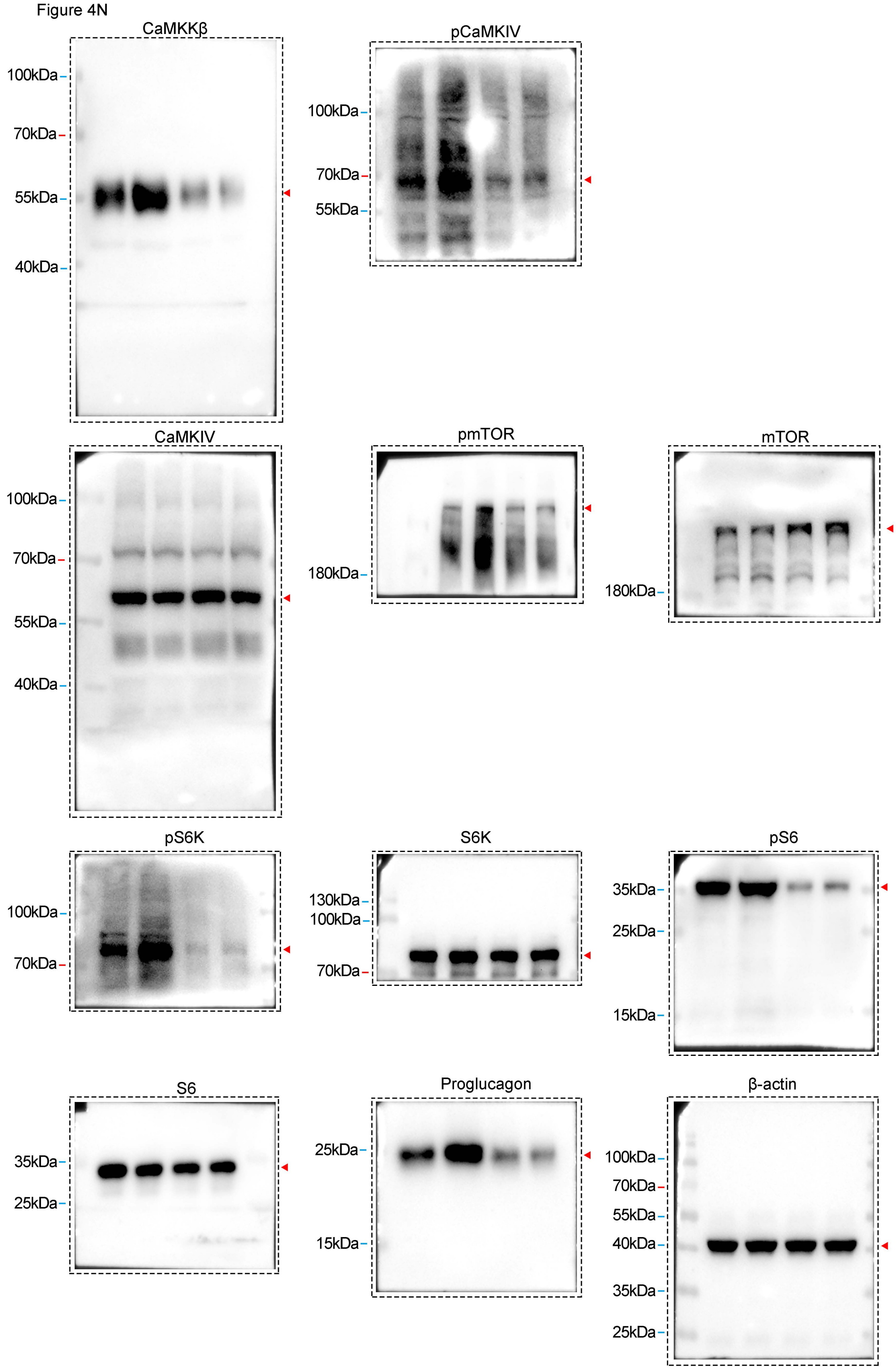

Figure 4, Source Data 1. Original membranes corresponding to Figure 4, panel N. The first lane represents the unstretched ileum of *Piezo1*$^{loxp/loxp}$ mice, the second lane shows the stretch-treated ileum of *Piezo1*$^{loxp/loxp}$ mice, the third lane depicts the unstretched ileum of *Piezo1* IntL-CKO mice, and the fourth lane illustrates the stretch-treated ileum of *Piezo1* IntL-CKO mice.
