# Supplementary material for: Mechano-regulation of GLP-1 production by Piezo1 in intestinal L cells
Source: eLife. 2024 Nov 7;13:RP97854. doi: 10.7554/eLife.97854 (PMC11542922; doi:10.7554/eLife.97854)
Supplement: Figure 5—source data 1. [file elife-97854-fig5-data1.zip › Figure5-source data 1.pdf]

Figure 5D

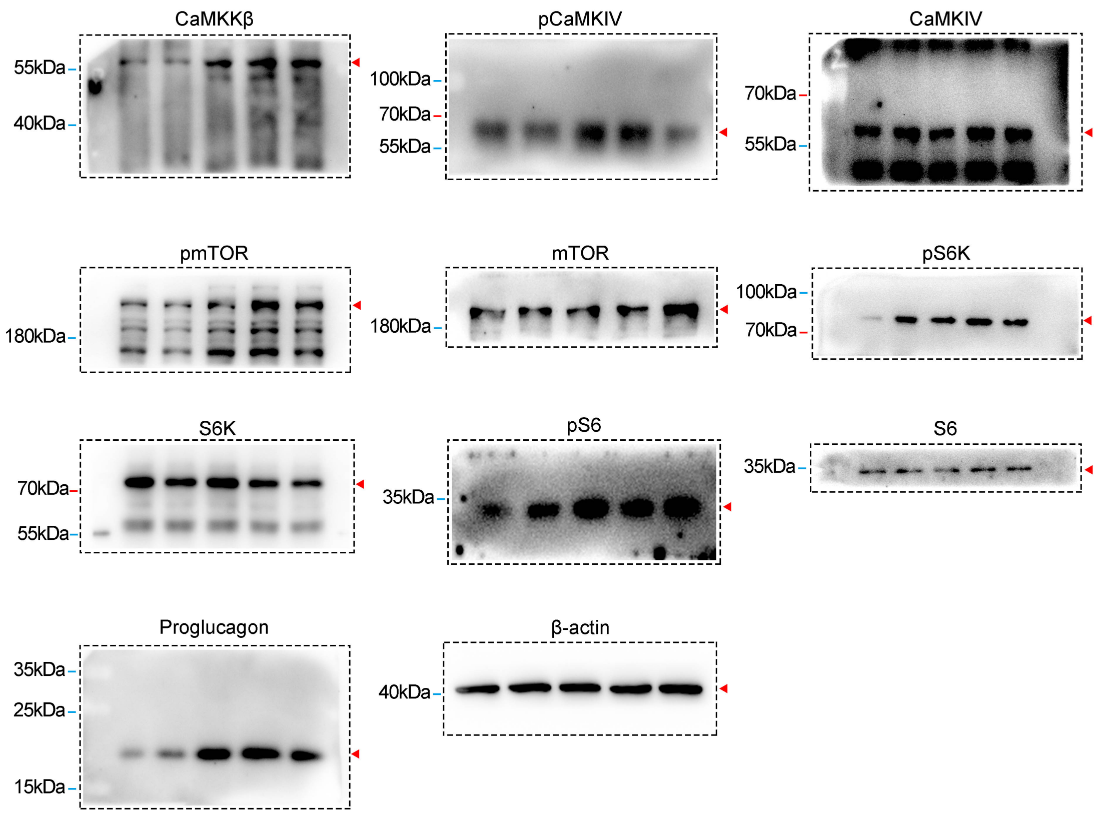

Figure 5, Source Data 1. Original membranes corresponding to Figure 5, panel D. From left to right, STC-1 cells treated with 0, 1.25, 2.5, 5, 10 μmol/L Yoda1, respectively.

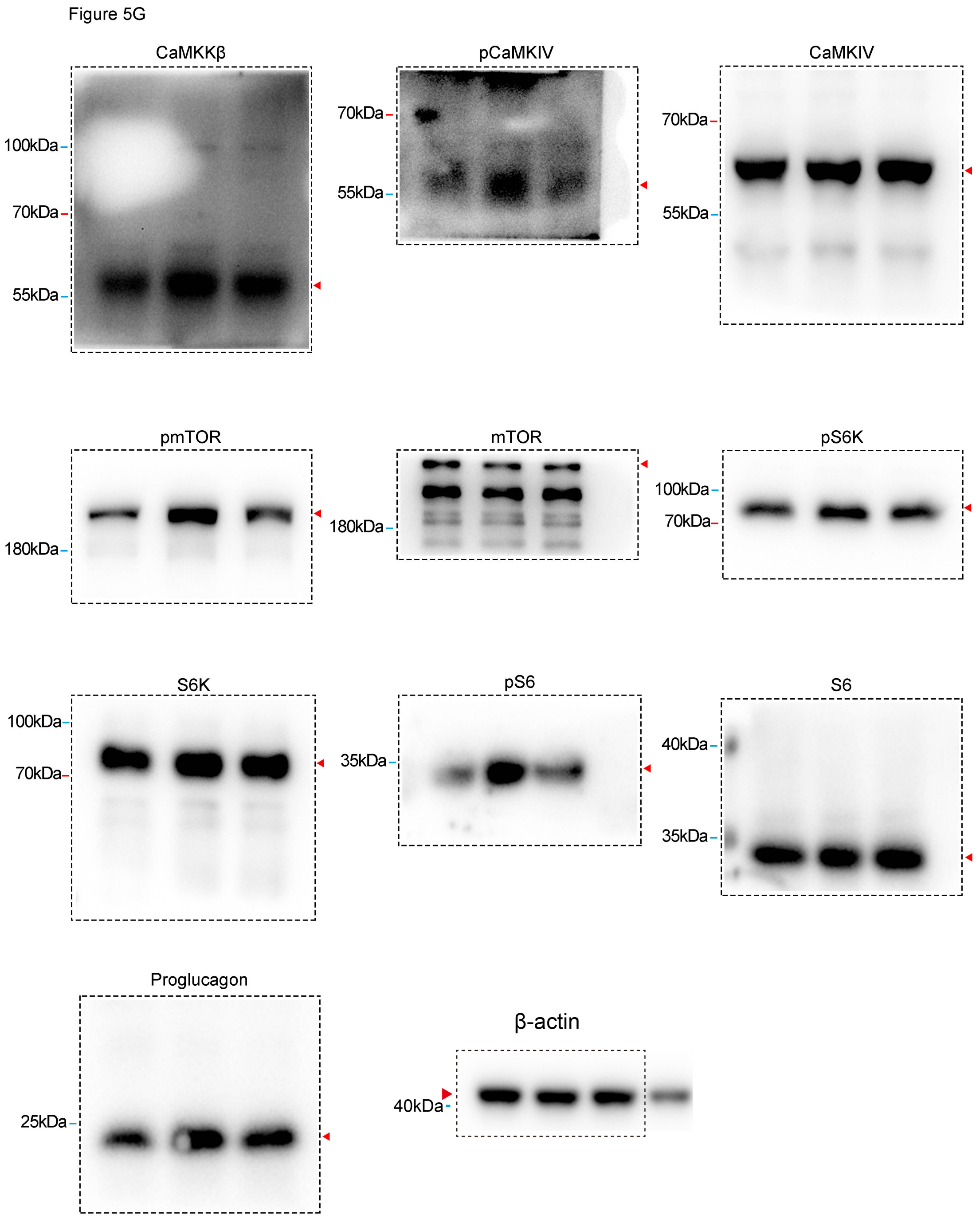

Figure 5, Source Data 1. Original membranes corresponding to Figure 5, panel G, showing the treatment of STC-1 cells. The first lane was treated with DMSO, the second lane with Yoda1, and the third lane with a combination of Yoda1 and GsMtx4.

Figure 5N

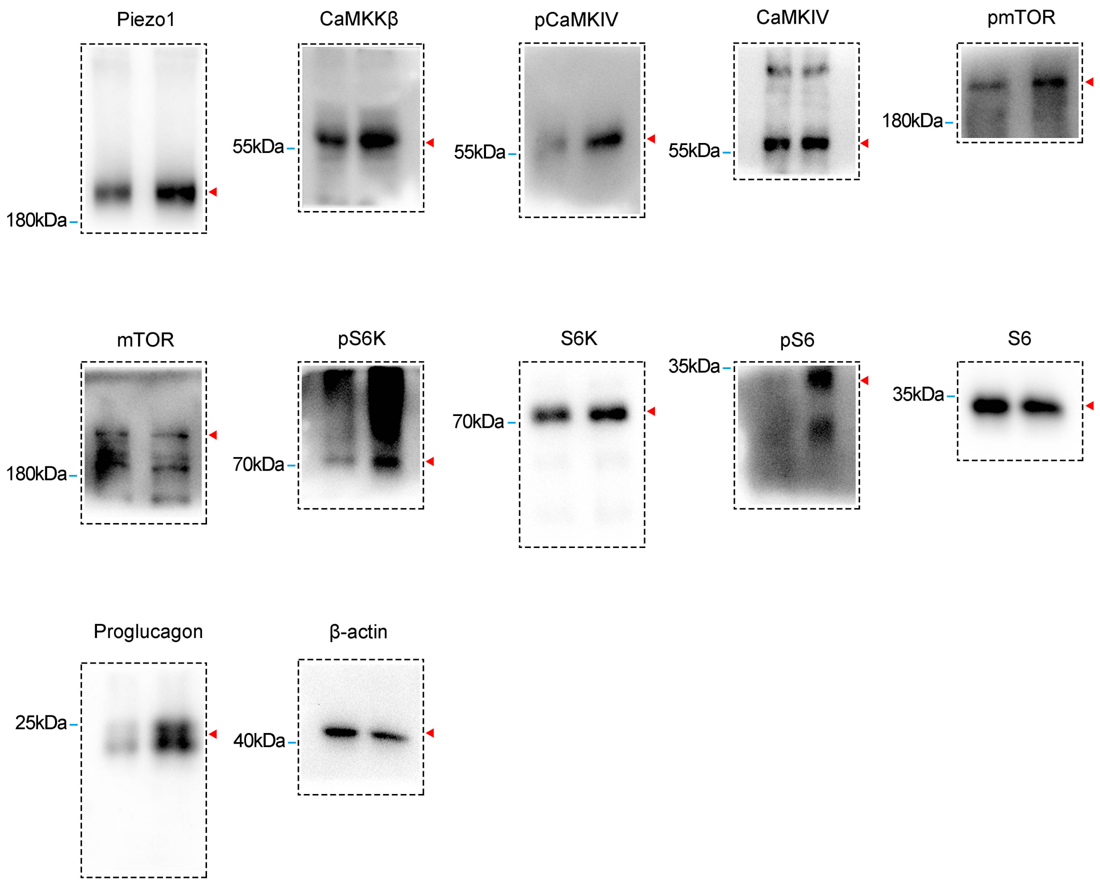

Figure 5, Source Data 1.Original membranes corresponding to Figure 5, panel N. Treatment of STC-1 cells: the first lane represents the unstretched treatment, while the second lane illustrates the stretched treatment.
