# Supplementary figures and images for: Mechano-regulation of GLP-1 production by Piezo1 in intestinal L cells (part 2 of 2)
Source: eLife. 2024 Nov 7;13:RP97854. doi: 10.7554/eLife.97854 (PMC11542922; doi:10.7554/eLife.97854)

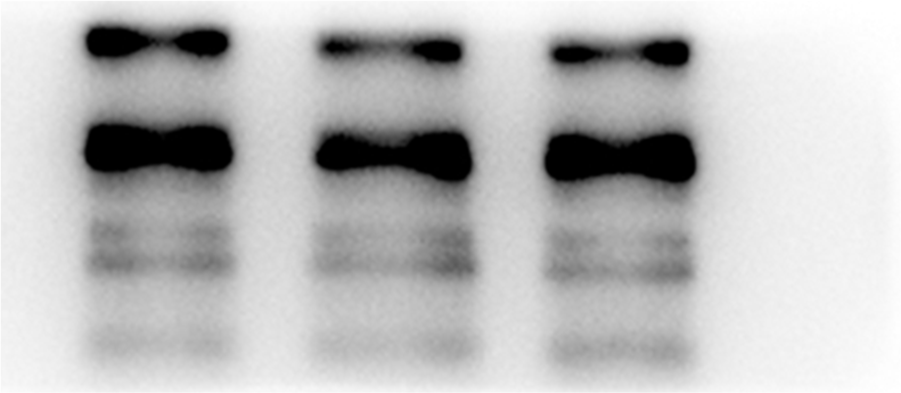

Supplement: Figure 5—source data 2. [file elife-97854-fig5-data2.zip › mTOR-5G.tif]

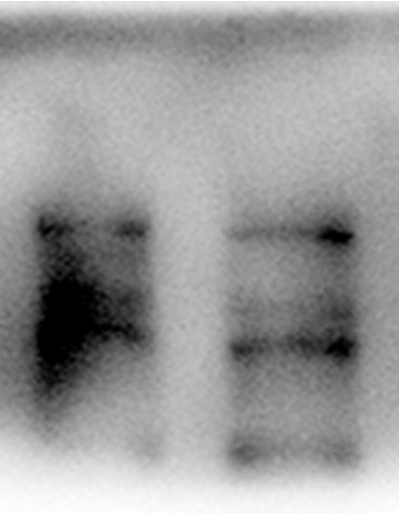

Supplement: Figure 5—source data 2. [file elife-97854-fig5-data2.zip › mTOR-5N.tif]

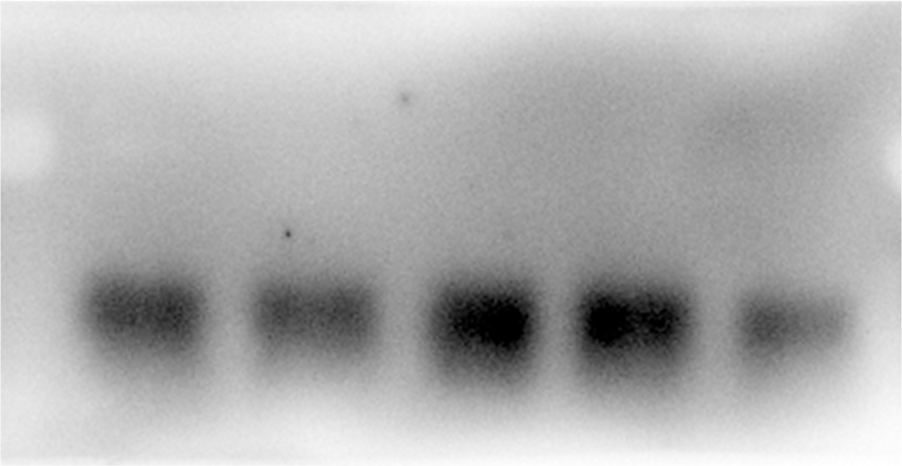

Supplement: Figure 5—source data 2. [file elife-97854-fig5-data2.zip › pCaMKIV-5D.tif]

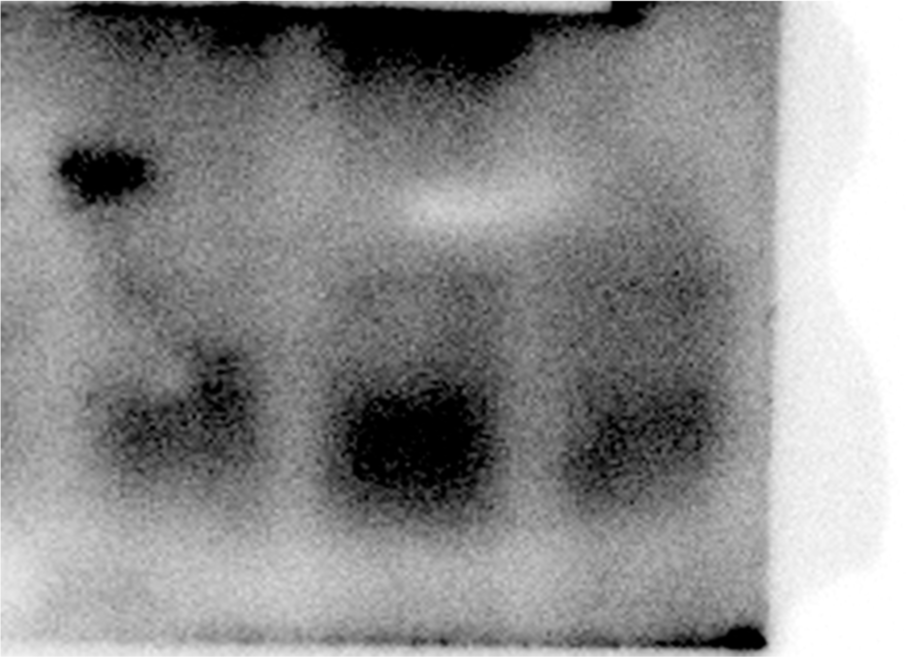

Supplement: Figure 5—source data 2. [file elife-97854-fig5-data2.zip › pCaMKIV-5G.tif]

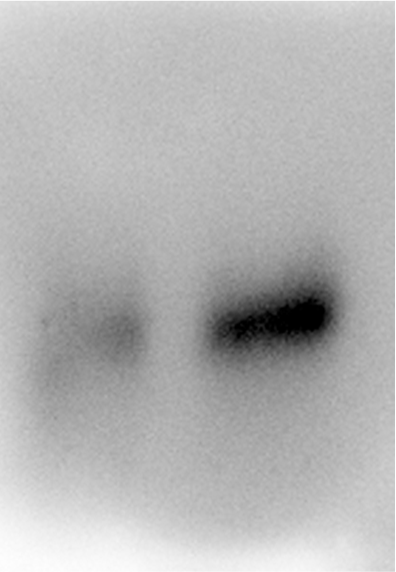

Supplement: Figure 5—source data 2. [file elife-97854-fig5-data2.zip › pCaMKIV-5N.tif]

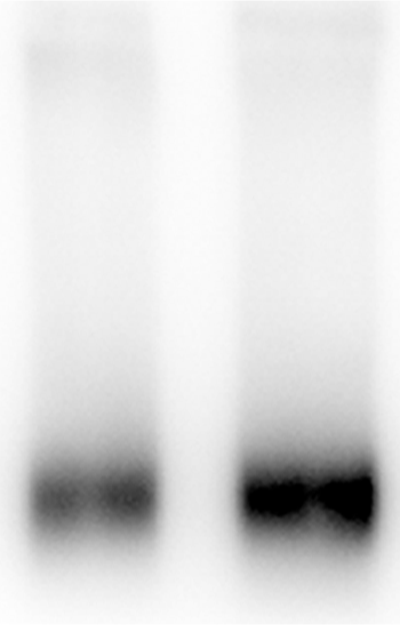

Supplement: Figure 5—source data 2. [file elife-97854-fig5-data2.zip › Piezo1-5N.tif]

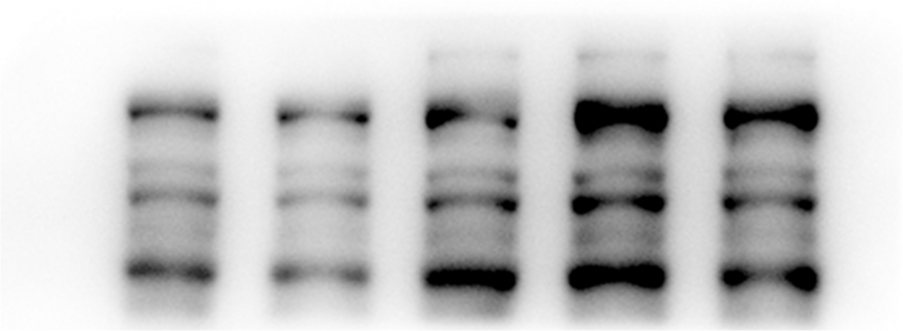

Supplement: Figure 5—source data 2. [file elife-97854-fig5-data2.zip › pmTOR-5D.tif]

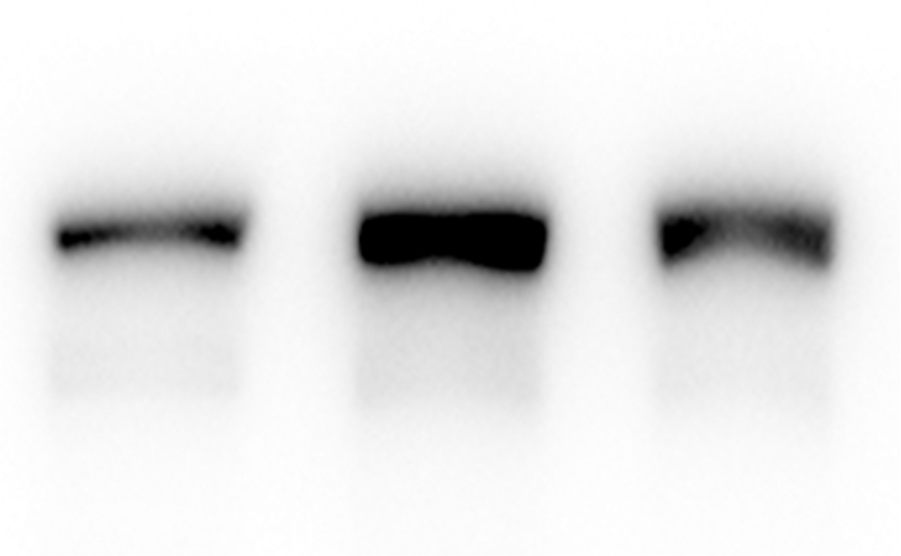

Supplement: Figure 5—source data 2. [file elife-97854-fig5-data2.zip › pmTOR-5G.tif]

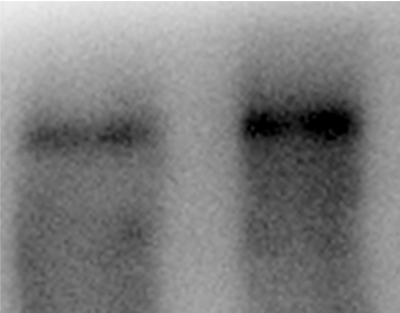

Supplement: Figure 5—source data 2. [file elife-97854-fig5-data2.zip › pmTOR-5N.tif]

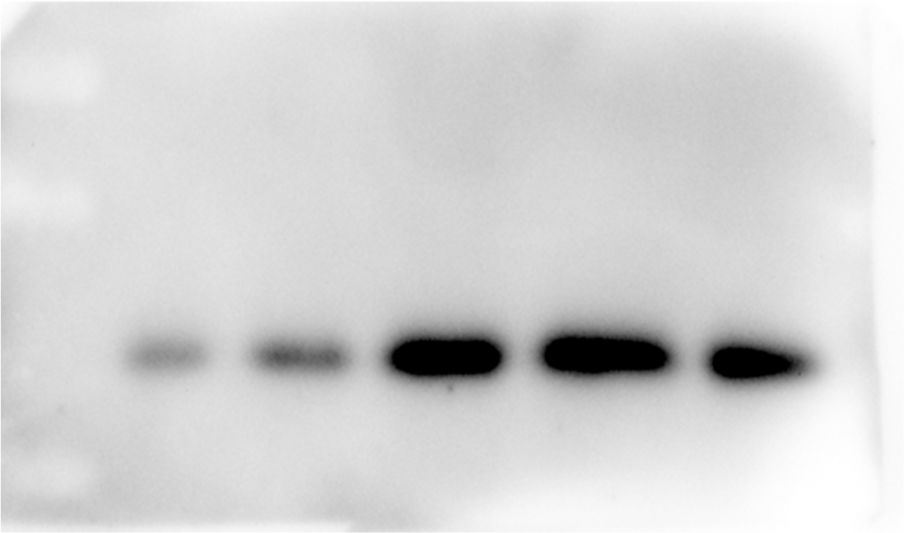

Supplement: Figure 5—source data 2. [file elife-97854-fig5-data2.zip › Proglucagen-5D.tif]

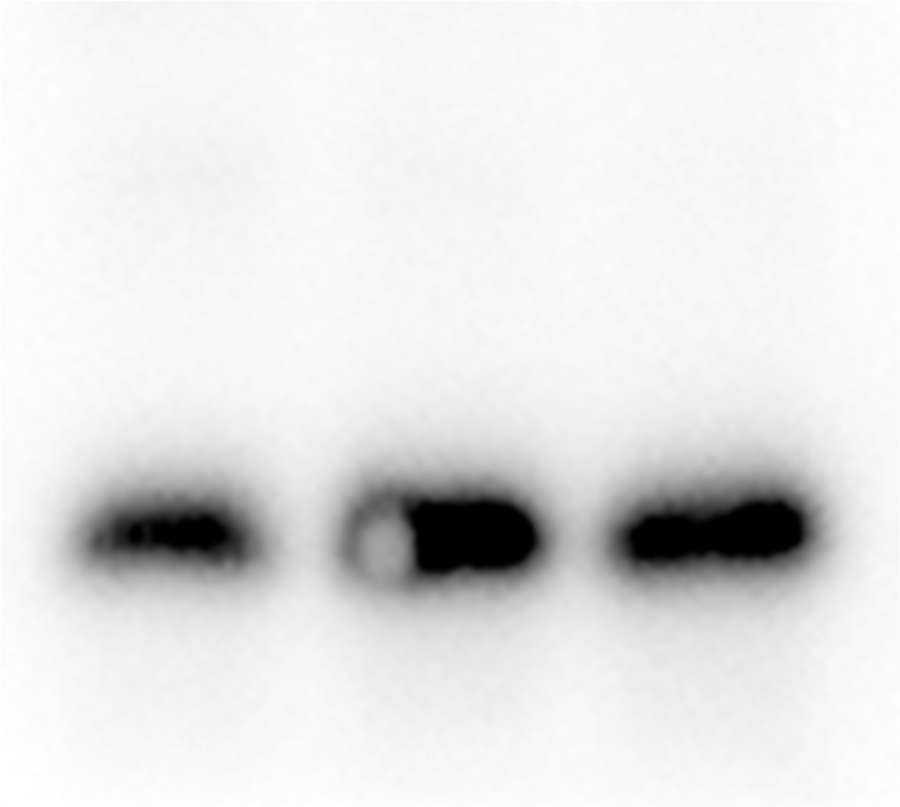

Supplement: Figure 5—source data 2. [file elife-97854-fig5-data2.zip › Proglucagen-5G.tif]

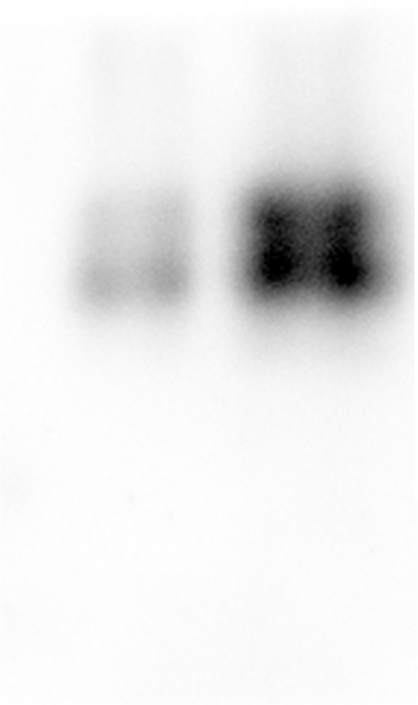

Supplement: Figure 5—source data 2. [file elife-97854-fig5-data2.zip › Proglucagen-5N.tif]

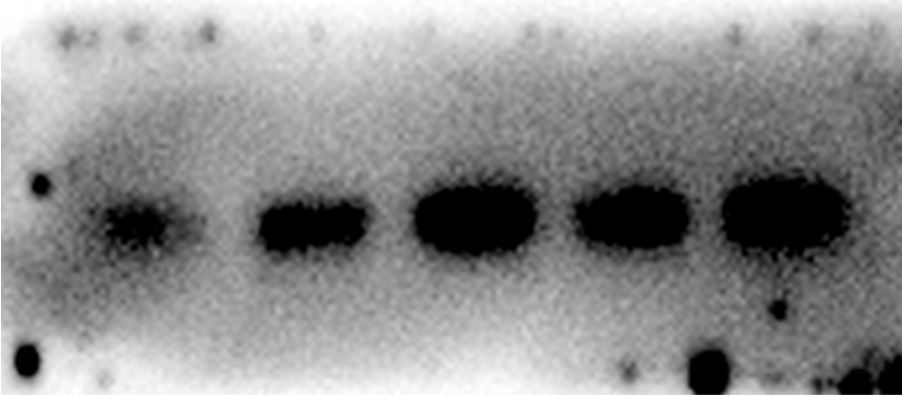

Supplement: Figure 5—source data 2. [file elife-97854-fig5-data2.zip › pS6-5D.tif]

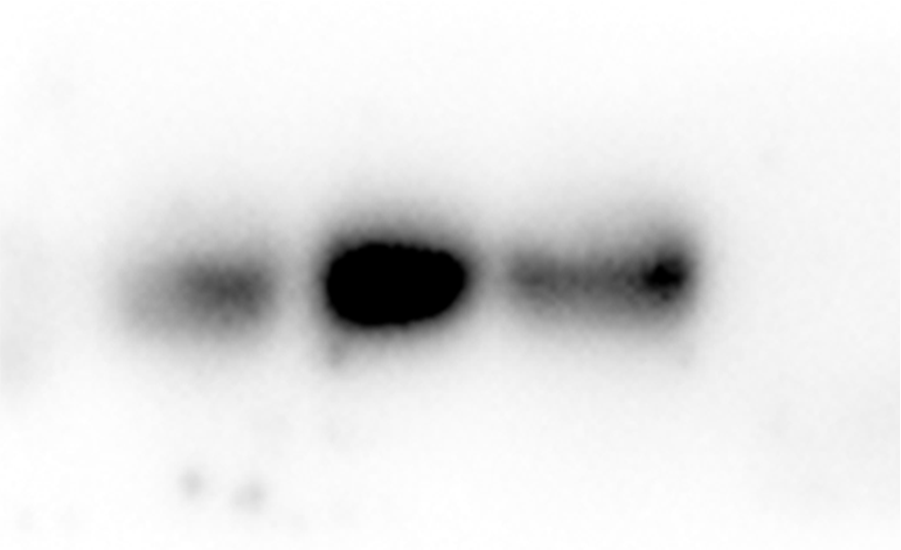

Supplement: Figure 5—source data 2. [file elife-97854-fig5-data2.zip › pS6-5G.tif]

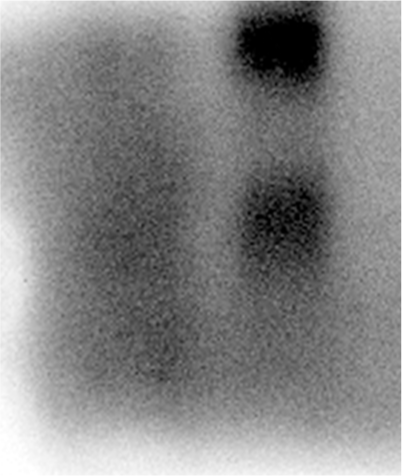

Supplement: Figure 5—source data 2. [file elife-97854-fig5-data2.zip › pS6-5N.tif]

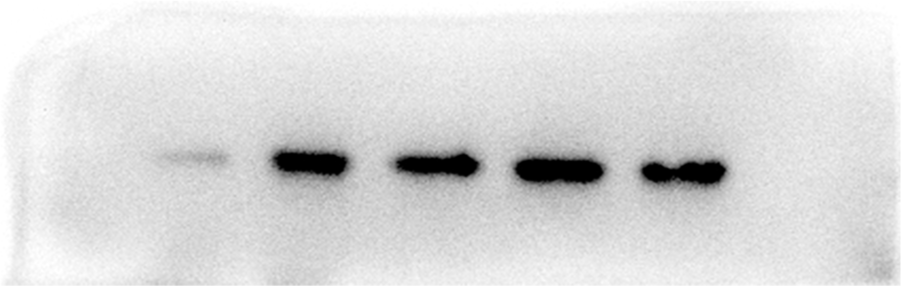

Supplement: Figure 5—source data 2. [file elife-97854-fig5-data2.zip › pS6K-5D.tif]

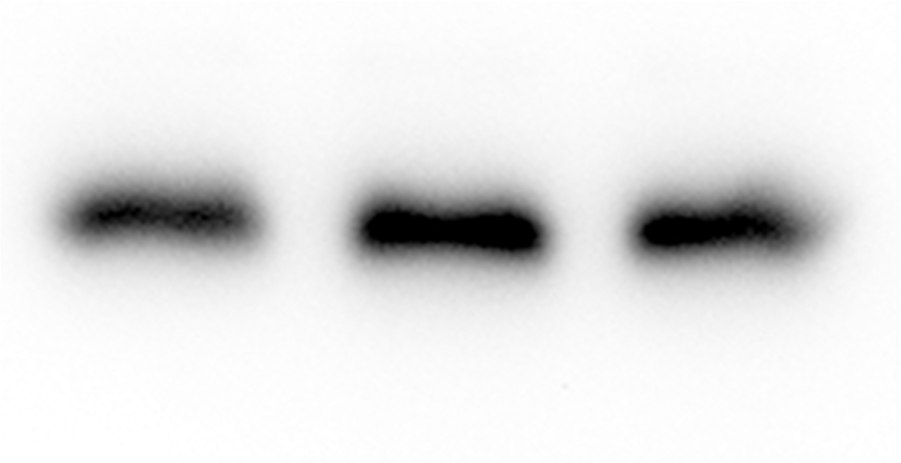

Supplement: Figure 5—source data 2. [file elife-97854-fig5-data2.zip › pS6K-5G.tif]

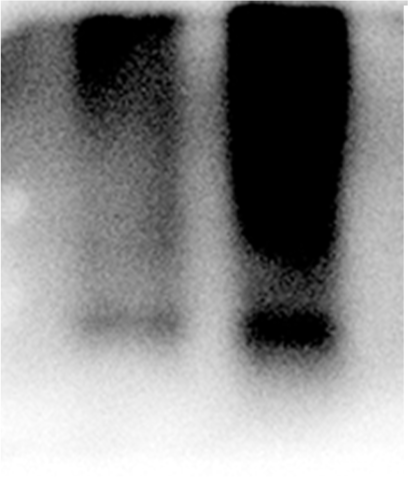

Supplement: Figure 5—source data 2. [file elife-97854-fig5-data2.zip › pS6K-5N.tif]

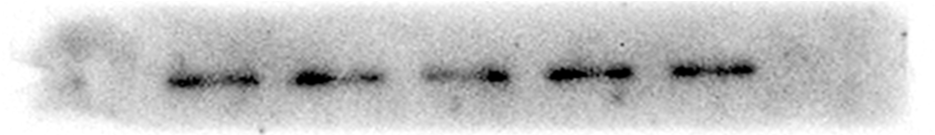

Supplement: Figure 5—source data 2. [file elife-97854-fig5-data2.zip › S6-5D.tif]

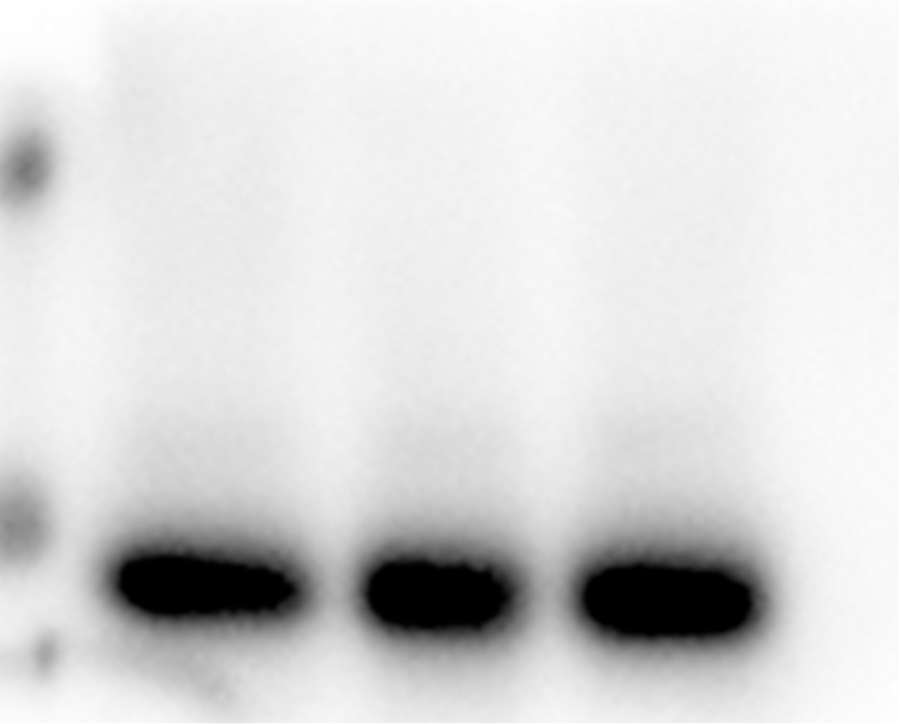

Supplement: Figure 5—source data 2. [file elife-97854-fig5-data2.zip › S6-5G.tif]

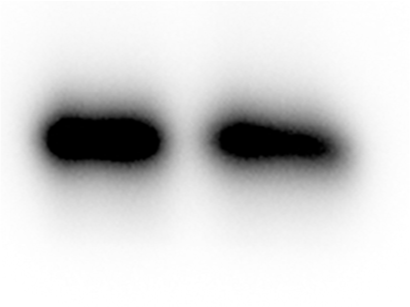

Supplement: Figure 5—source data 2. [file elife-97854-fig5-data2.zip › S6-5N.tif]

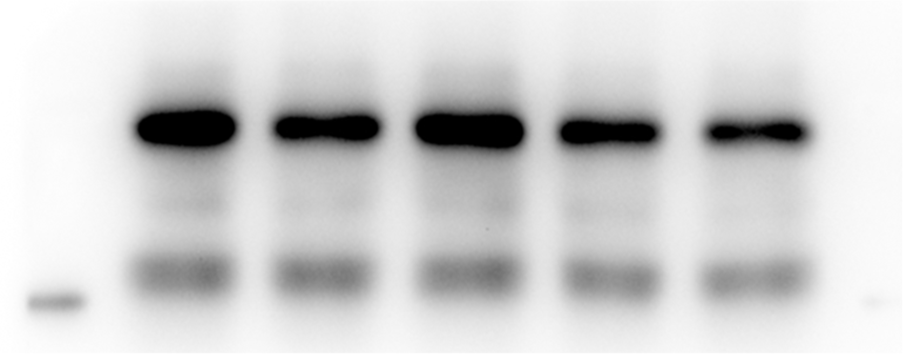

Supplement: Figure 5—source data 2. [file elife-97854-fig5-data2.zip › S6K-5D.tif]

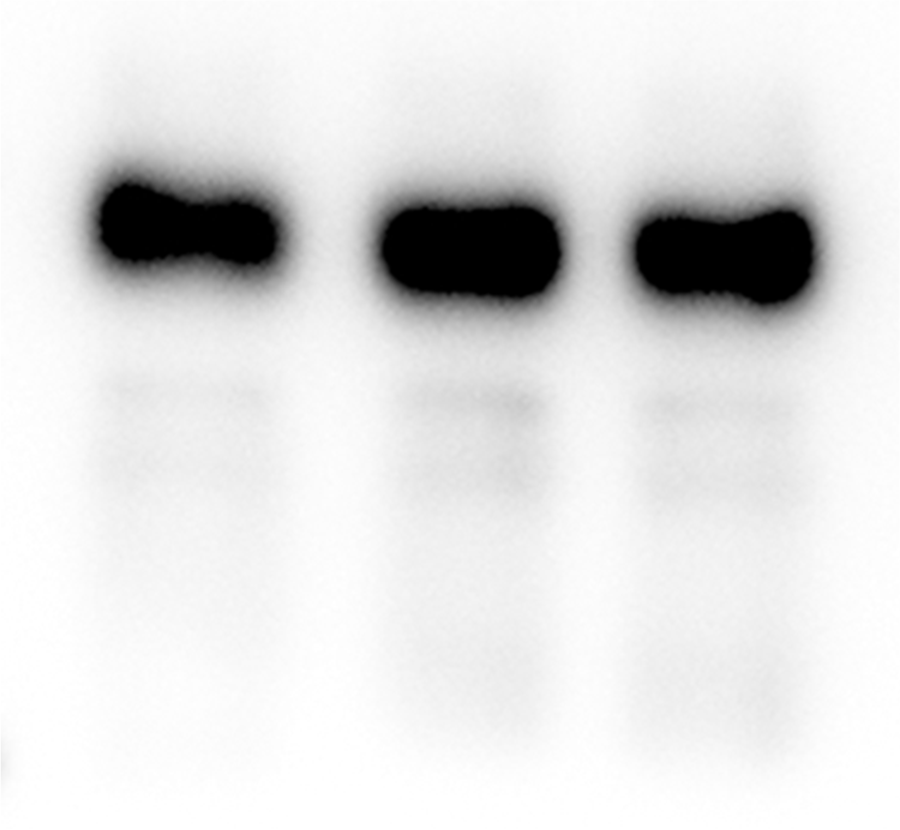

Supplement: Figure 5—source data 2. [file elife-97854-fig5-data2.zip › S6K-5G.tif]

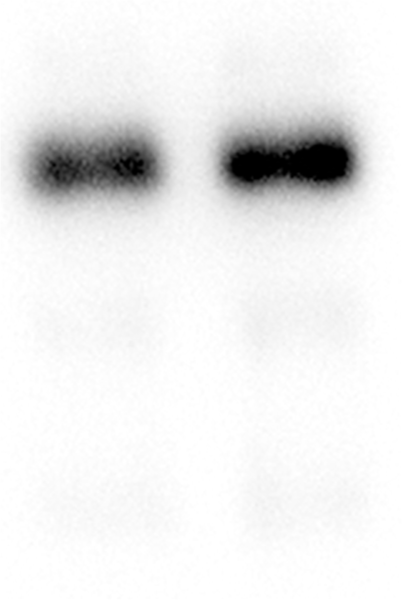

Supplement: Figure 5—source data 2. [file elife-97854-fig5-data2.zip › S6K-5N.tif]

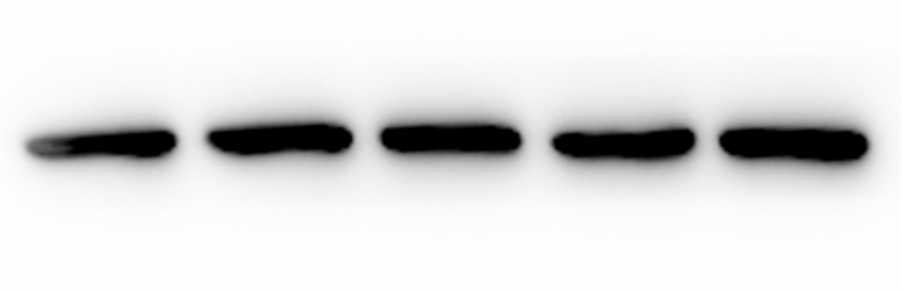

Supplement: Figure 5—source data 2. [file elife-97854-fig5-data2.zip › ╬▓-actin-5D.tif]

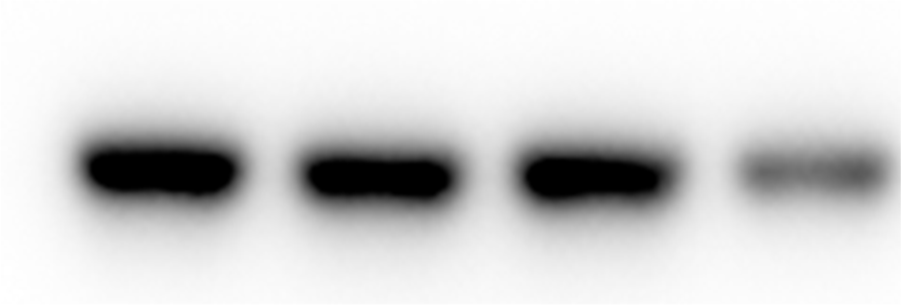

Supplement: Figure 5—source data 2. [file elife-97854-fig5-data2.zip › ╬▓-actin-5G.tif]

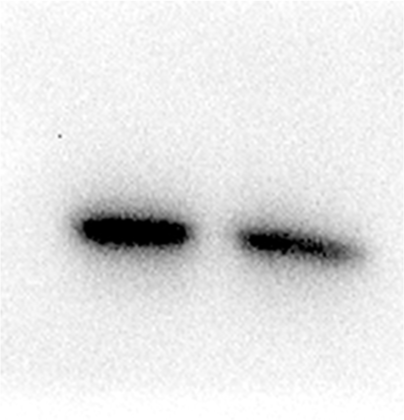

Supplement: Figure 5—source data 2. [file elife-97854-fig5-data2.zip › ╬▓-actin-5N.tif]

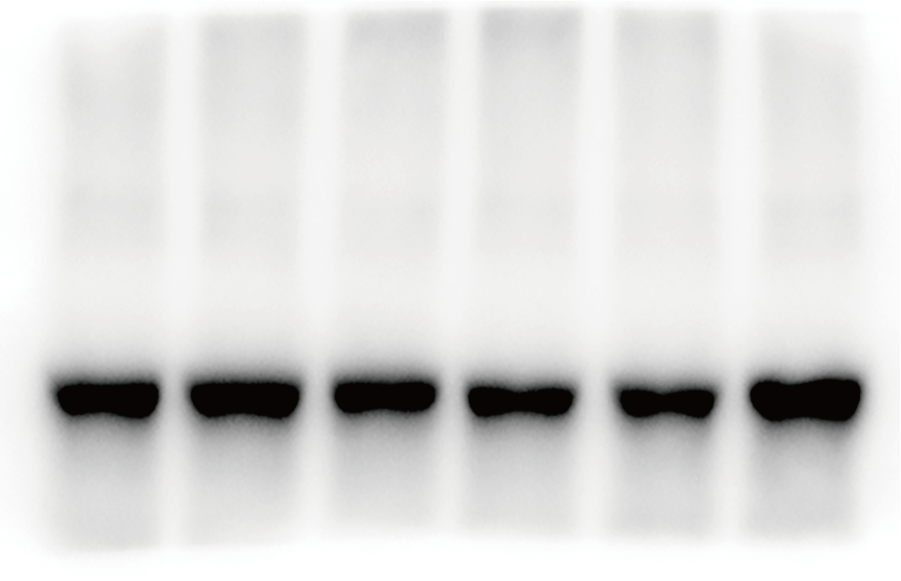

Supplement: Figure 6—source data 2. [file elife-97854-fig6-data2.zip › CaMKIV-6D.tif]

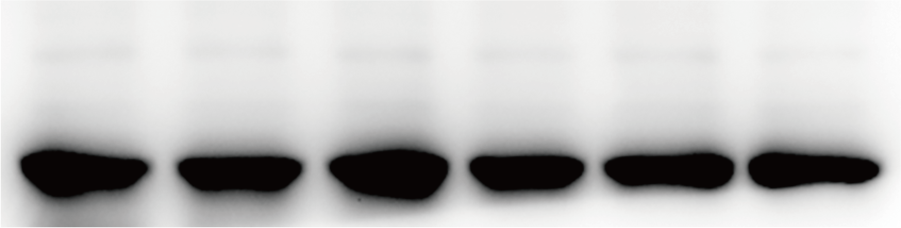

Supplement: Figure 6—source data 2. [file elife-97854-fig6-data2.zip › CaMKIV-6H.tif]

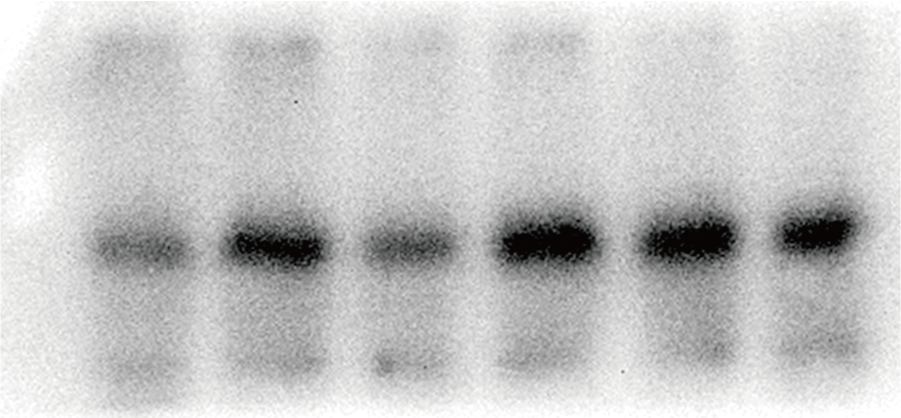

Supplement: Figure 6—source data 2. [file elife-97854-fig6-data2.zip › CaMKK╬▓-6D.tif]

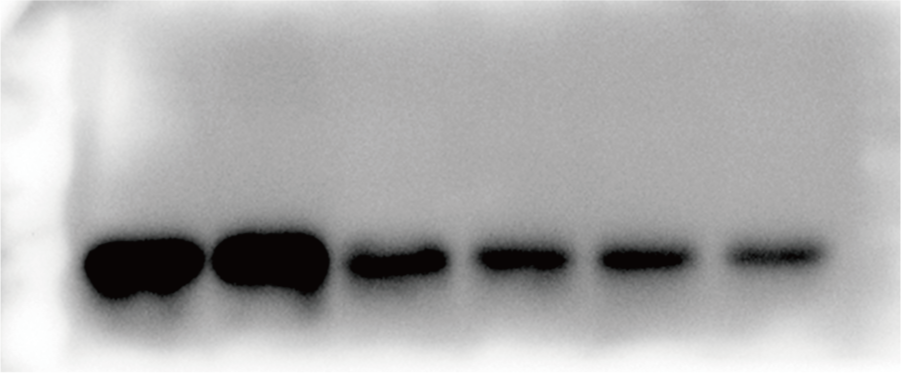

Supplement: Figure 6—source data 2. [file elife-97854-fig6-data2.zip › CaMKK╬▓-6H.tif]

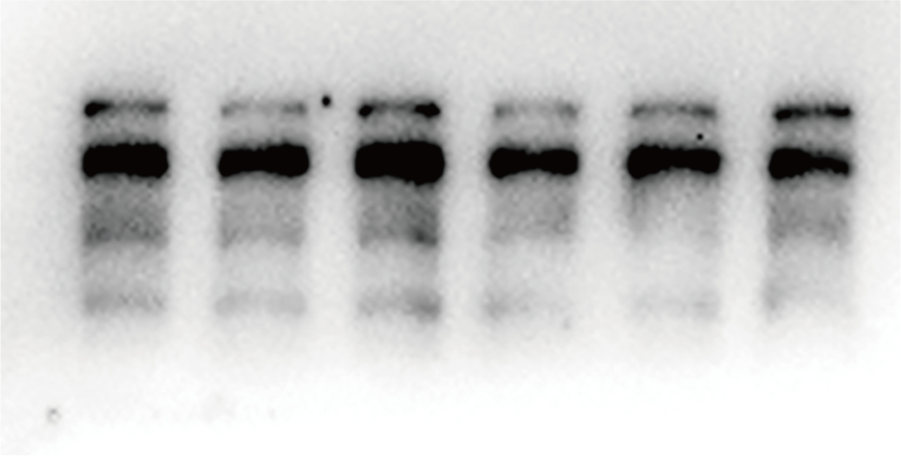

Supplement: Figure 6—source data 2. [file elife-97854-fig6-data2.zip › mTOR-6D.tif]

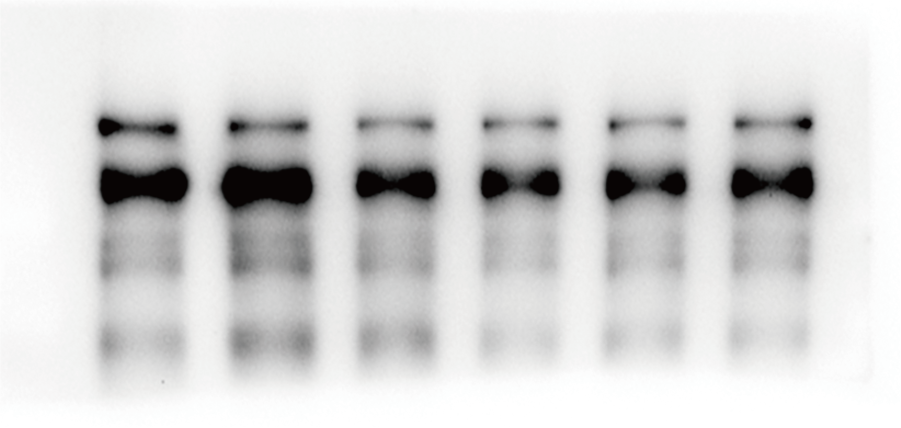

Supplement: Figure 6—source data 2. [file elife-97854-fig6-data2.zip › mTOR-6H.tif]

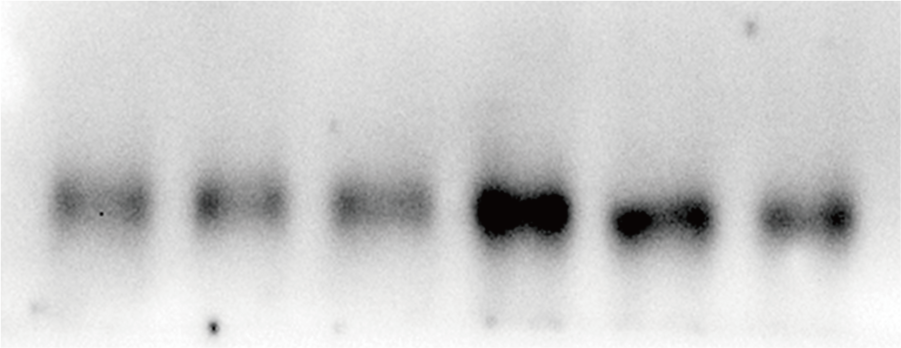

Supplement: Figure 6—source data 2. [file elife-97854-fig6-data2.zip › pCaMKIV-6D.tif]

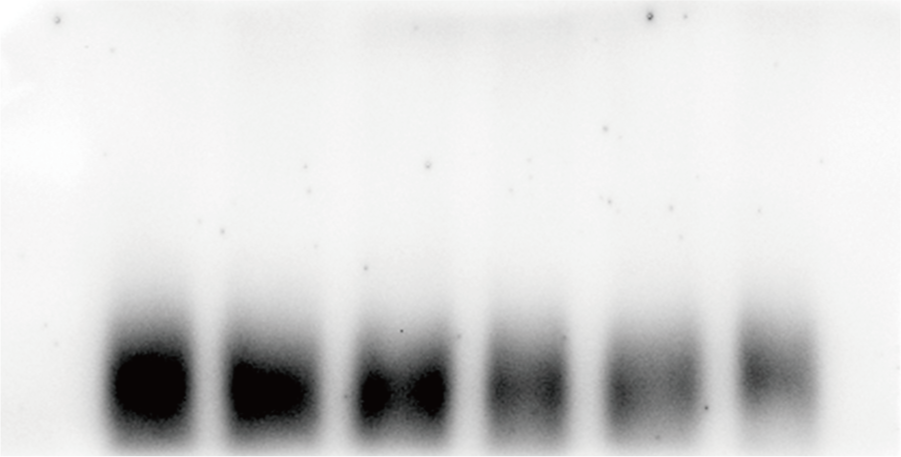

Supplement: Figure 6—source data 2. [file elife-97854-fig6-data2.zip › pCaMKIV-6H.tif]

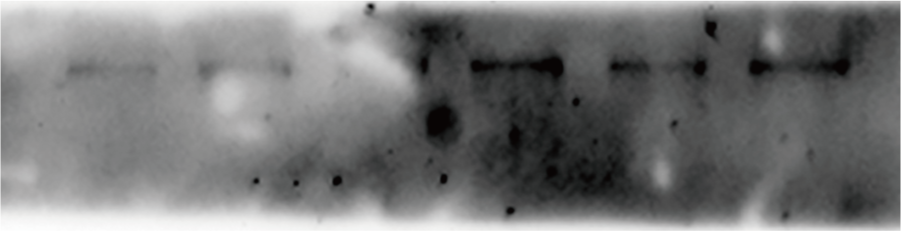

Supplement: Figure 6—source data 2. [file elife-97854-fig6-data2.zip › Piezo1-6D.tif]

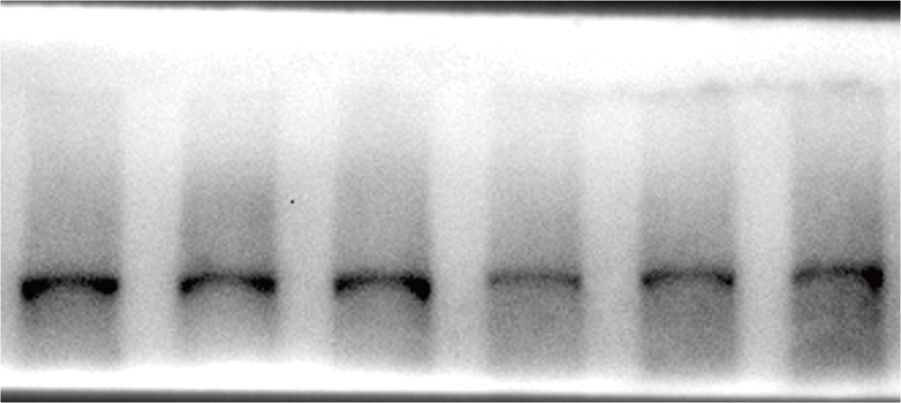

Supplement: Figure 6—source data 2. [file elife-97854-fig6-data2.zip › Piezo1-6H.tif]

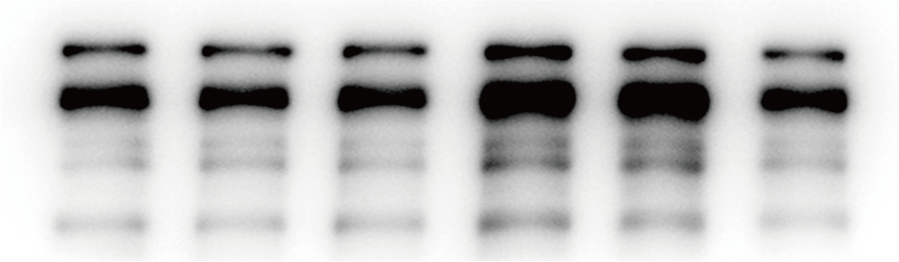

Supplement: Figure 6—source data 2. [file elife-97854-fig6-data2.zip › pmTOR-6D.tif]

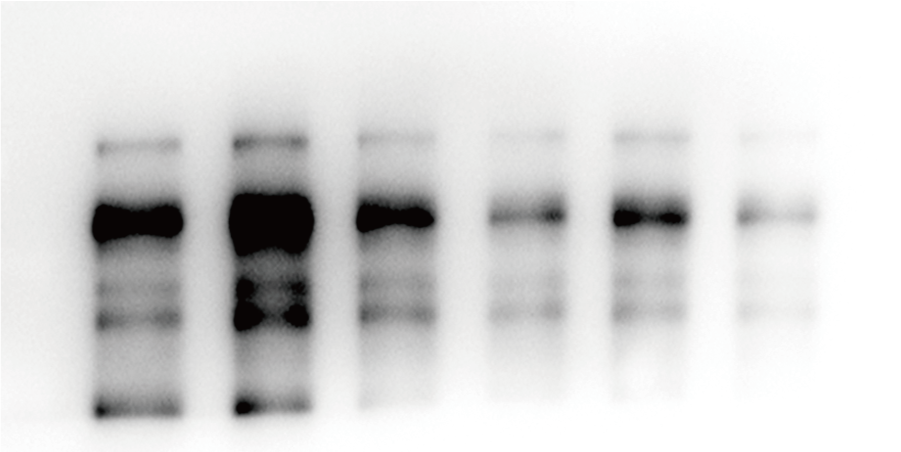

Supplement: Figure 6—source data 2. [file elife-97854-fig6-data2.zip › pmTOR-6H.tif]

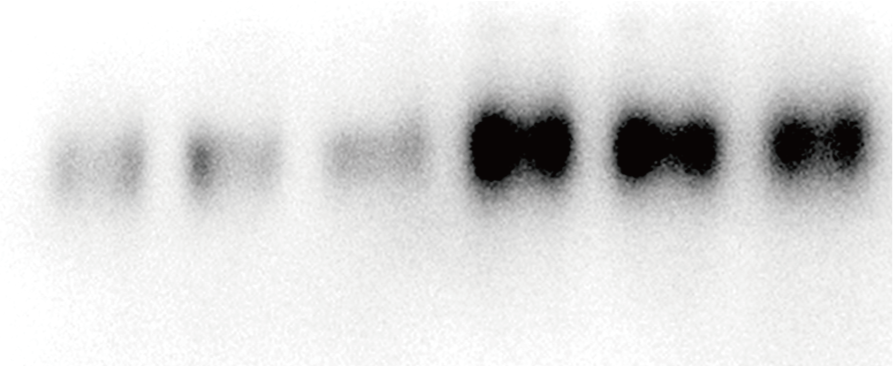

Supplement: Figure 6—source data 2. [file elife-97854-fig6-data2.zip › Proglucagon-6D.tif]

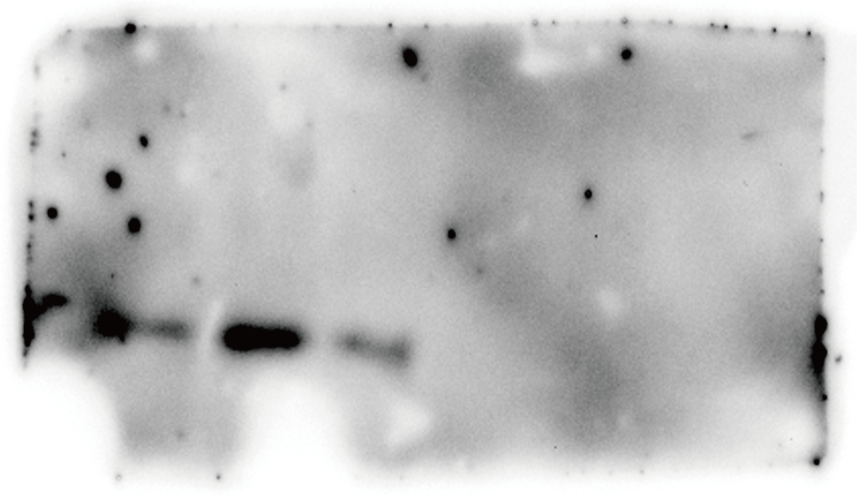

Supplement: Figure 6—source data 2. [file elife-97854-fig6-data2.zip › Proglucagon-6H.tif]

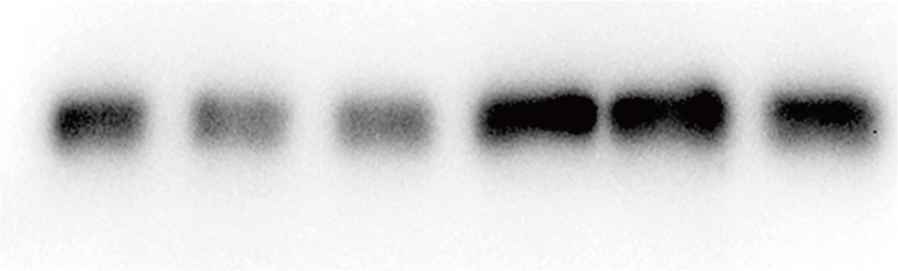

Supplement: Figure 6—source data 2. [file elife-97854-fig6-data2.zip › pS6-6D.tif]

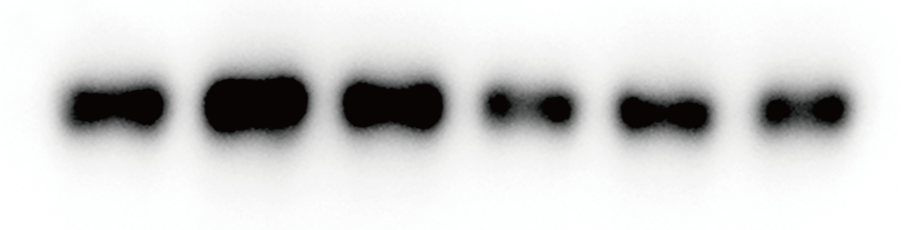

Supplement: Figure 6—source data 2. [file elife-97854-fig6-data2.zip › pS6-6H.tif]

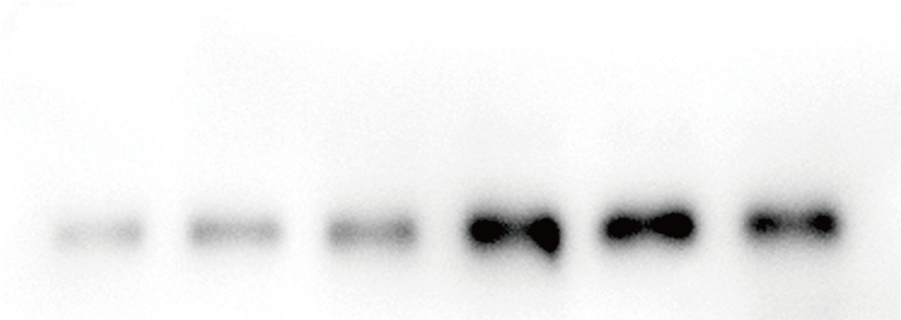

Supplement: Figure 6—source data 2. [file elife-97854-fig6-data2.zip › pS6K-6D.tif]

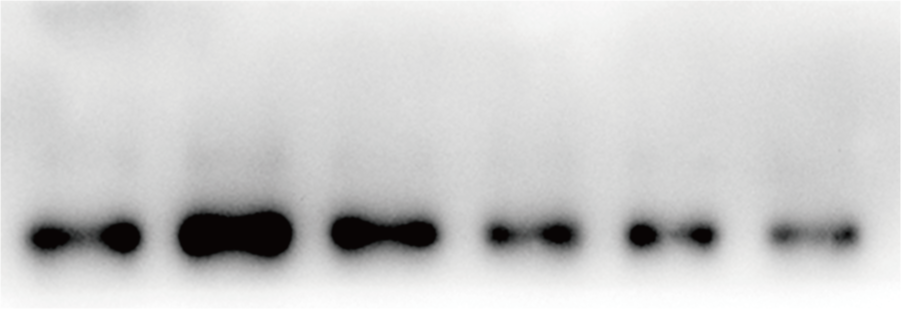

Supplement: Figure 6—source data 2. [file elife-97854-fig6-data2.zip › pS6K-6H.tif]

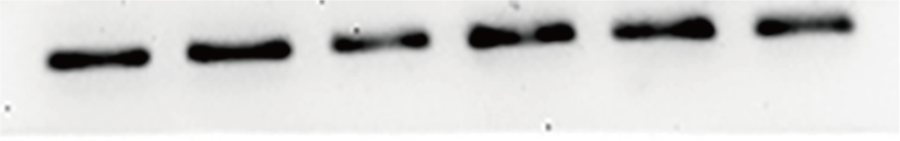

Supplement: Figure 6—source data 2. [file elife-97854-fig6-data2.zip › S6-6D.tif]

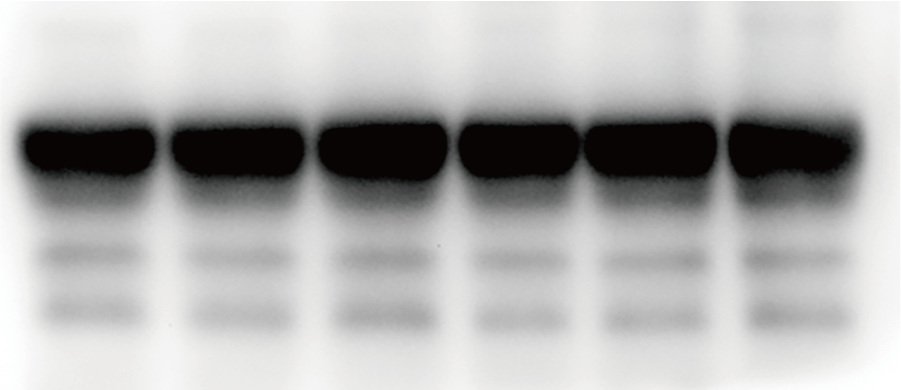

Supplement: Figure 6—source data 2. [file elife-97854-fig6-data2.zip › S6-6H.tif]

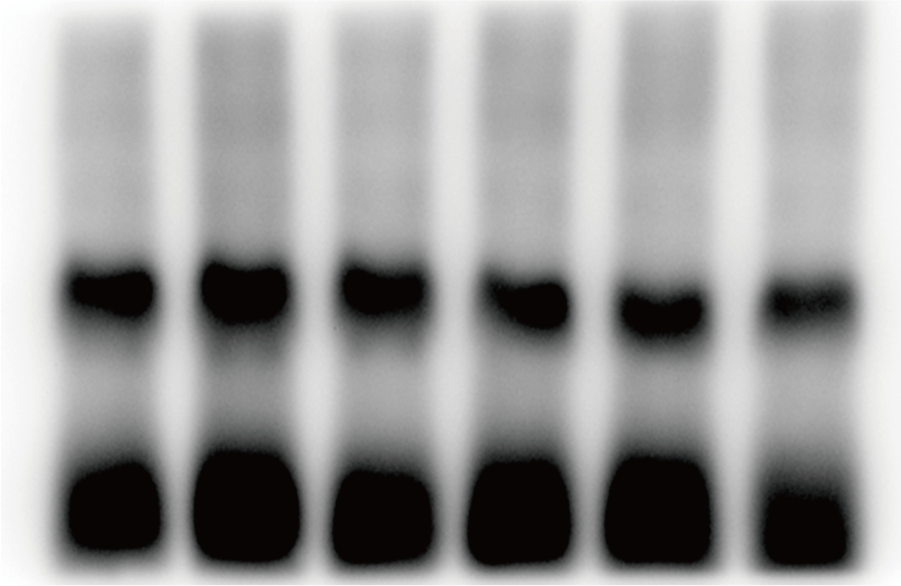

Supplement: Figure 6—source data 2. [file elife-97854-fig6-data2.zip › S6K-6D.tif]

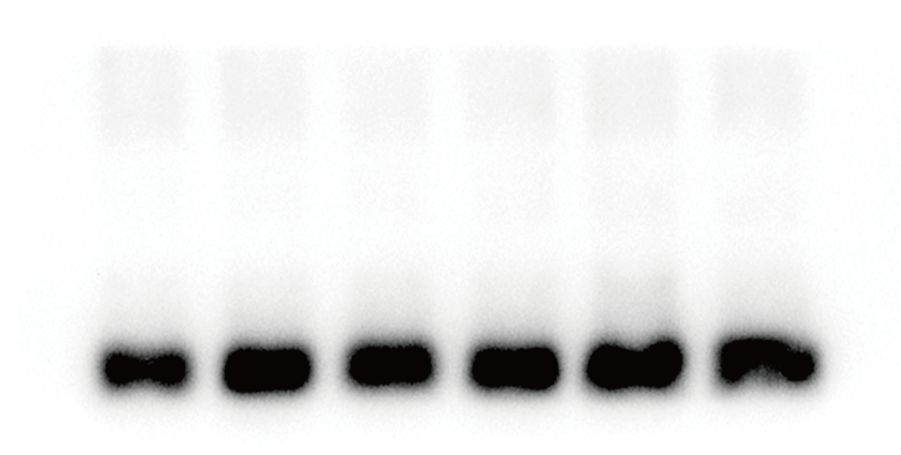

Supplement: Figure 6—source data 2. [file elife-97854-fig6-data2.zip › S6K-6H.tif]

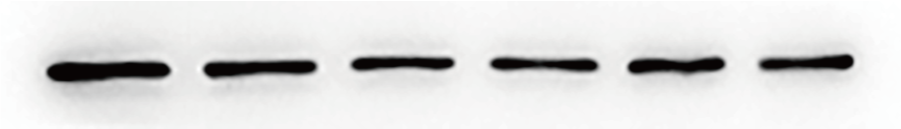

Supplement: Figure 6—source data 2. [file elife-97854-fig6-data2.zip › ╬▓-actin-6D.tif]

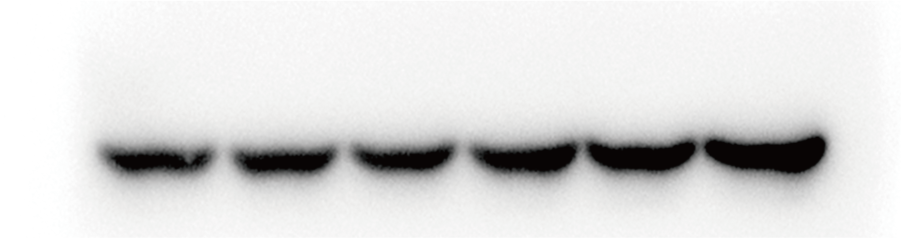

Supplement: Figure 6—source data 2. [file elife-97854-fig6-data2.zip › ╬▓-actin-6H.tif]

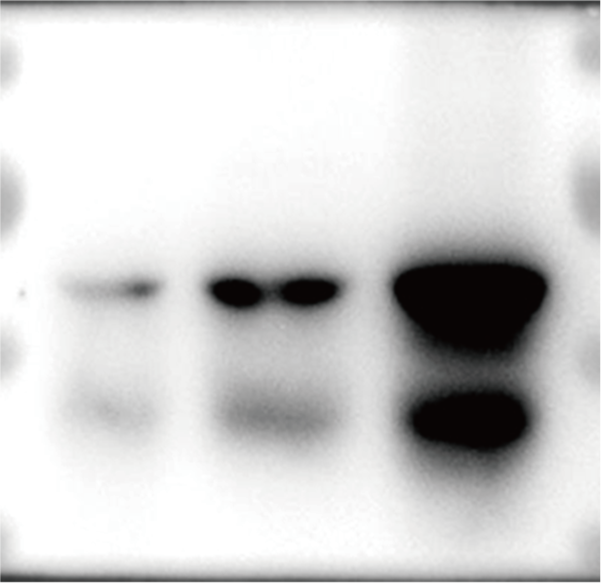

Supplement: Figure 7—source data 2. [file elife-97854-fig7-data2.zip › CaMKIV-7C.tif]

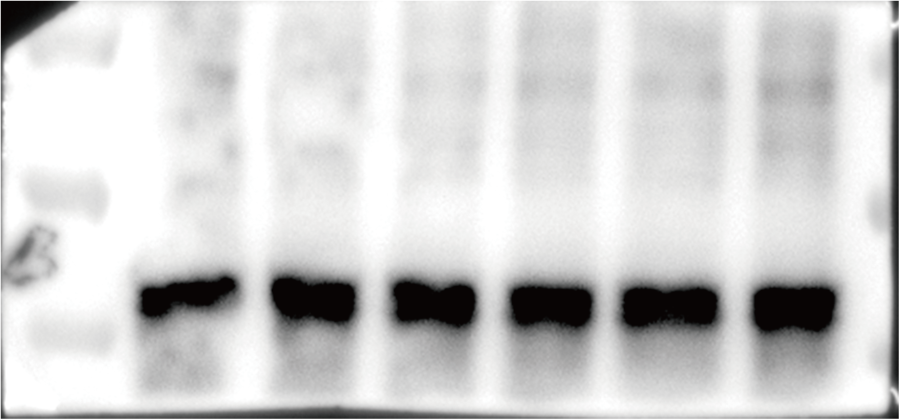

Supplement: Figure 7—source data 2. [file elife-97854-fig7-data2.zip › CaMKIV-7F.tif]

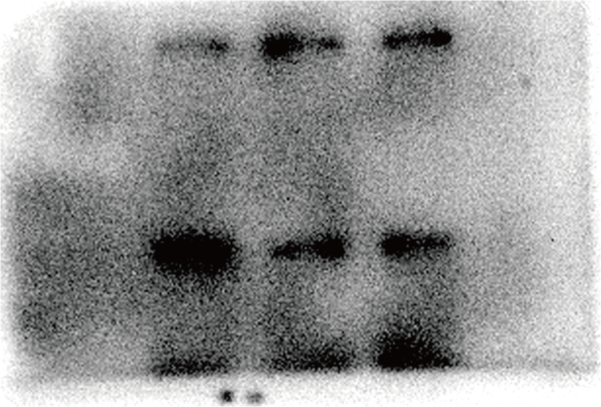

Supplement: Figure 7—source data 2. [file elife-97854-fig7-data2.zip › CaMKK╬▓-7C.tif]

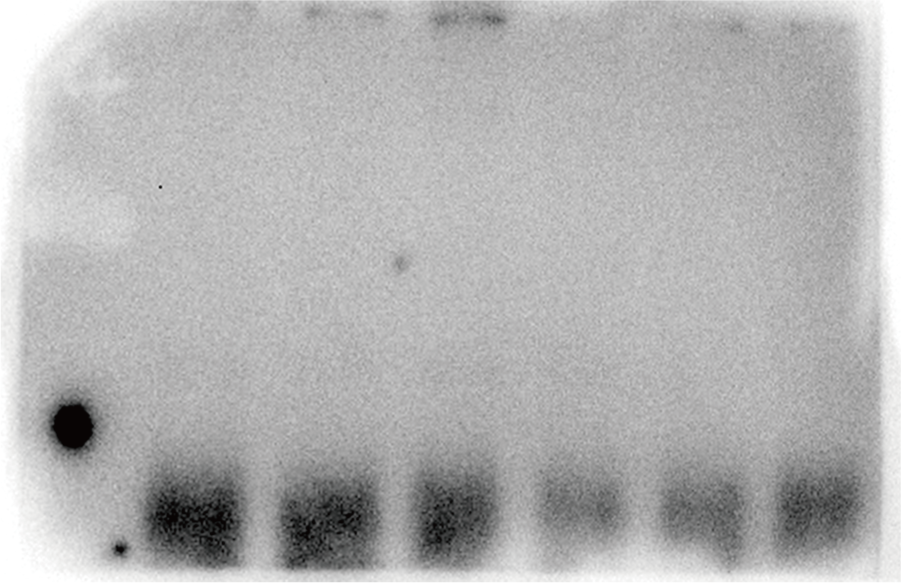

Supplement: Figure 7—source data 2. [file elife-97854-fig7-data2.zip › CaMKK╬▓-7F.tif]

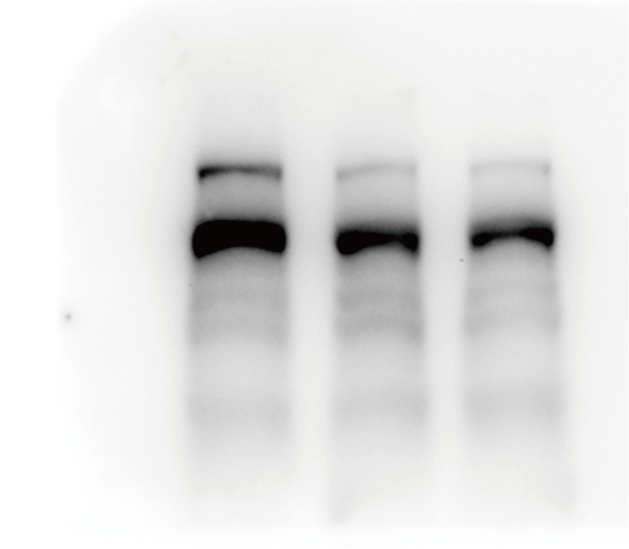

Supplement: Figure 7—source data 2. [file elife-97854-fig7-data2.zip › mTOR-7C.tif]

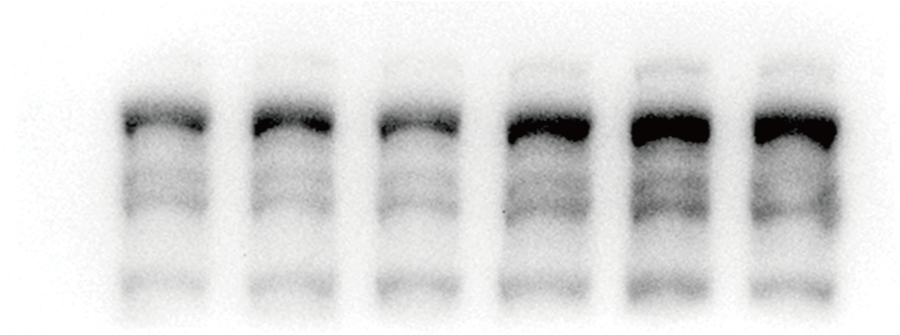

Supplement: Figure 7—source data 2. [file elife-97854-fig7-data2.zip › mTOR-7F.tif]

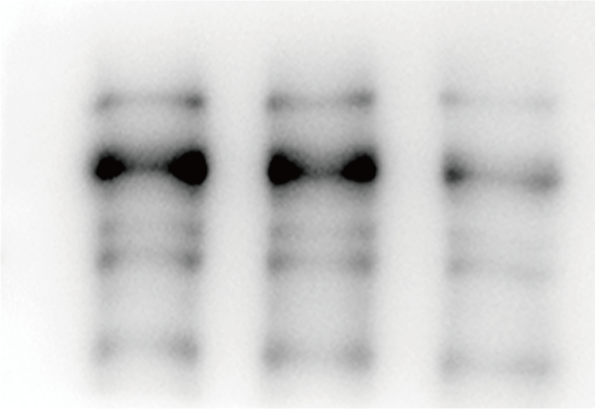

Supplement: Figure 7—source data 2. [file elife-97854-fig7-data2.zip › mTOR-7I.tif]

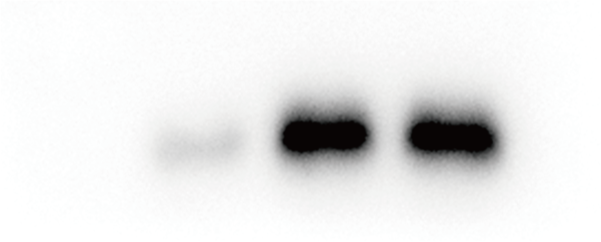

Supplement: Figure 7—source data 2. [file elife-97854-fig7-data2.zip › pCaMKIV-7C.tif]

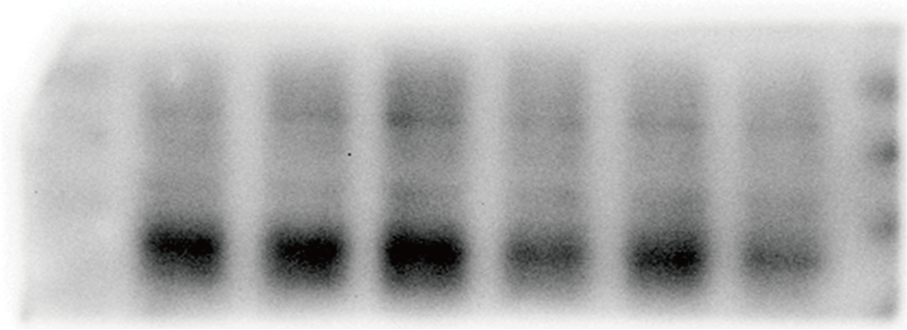

Supplement: Figure 7—source data 2. [file elife-97854-fig7-data2.zip › pCaMKIV-7F.tif]

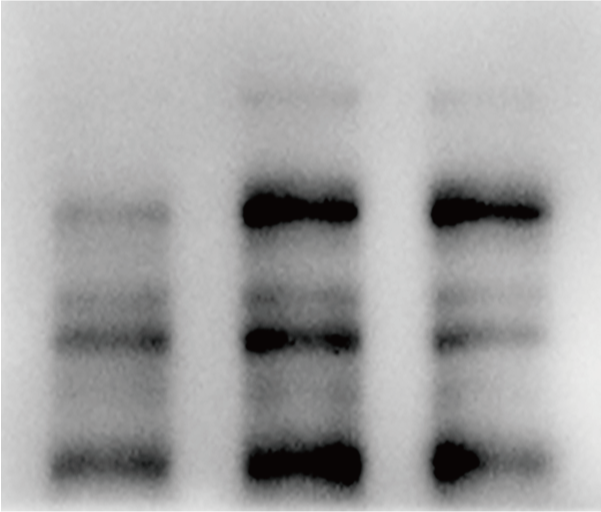

Supplement: Figure 7—source data 2. [file elife-97854-fig7-data2.zip › pmTOR-7C.tif]

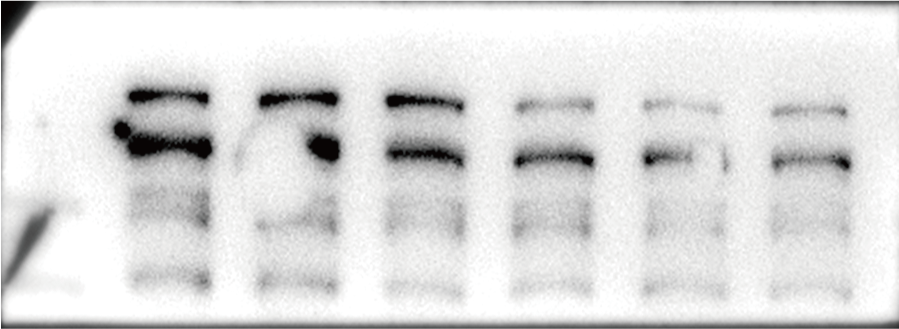

Supplement: Figure 7—source data 2. [file elife-97854-fig7-data2.zip › pmTOR-7F.tif]

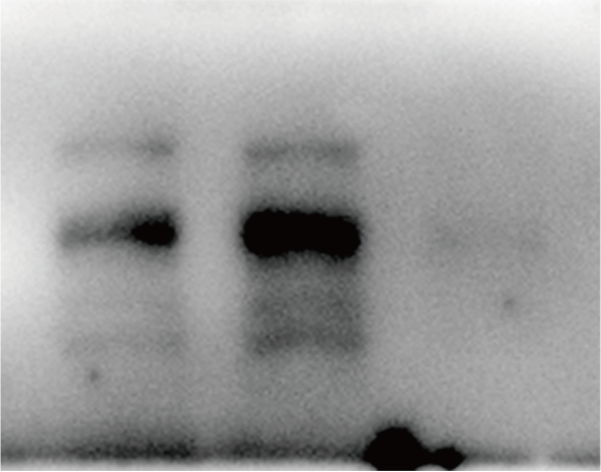

Supplement: Figure 7—source data 2. [file elife-97854-fig7-data2.zip › pmTOR-7I.tif]

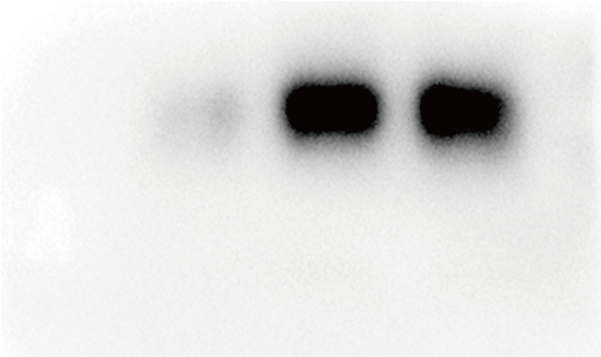

Supplement: Figure 7—source data 2. [file elife-97854-fig7-data2.zip › Proglucagon-7C.tif]

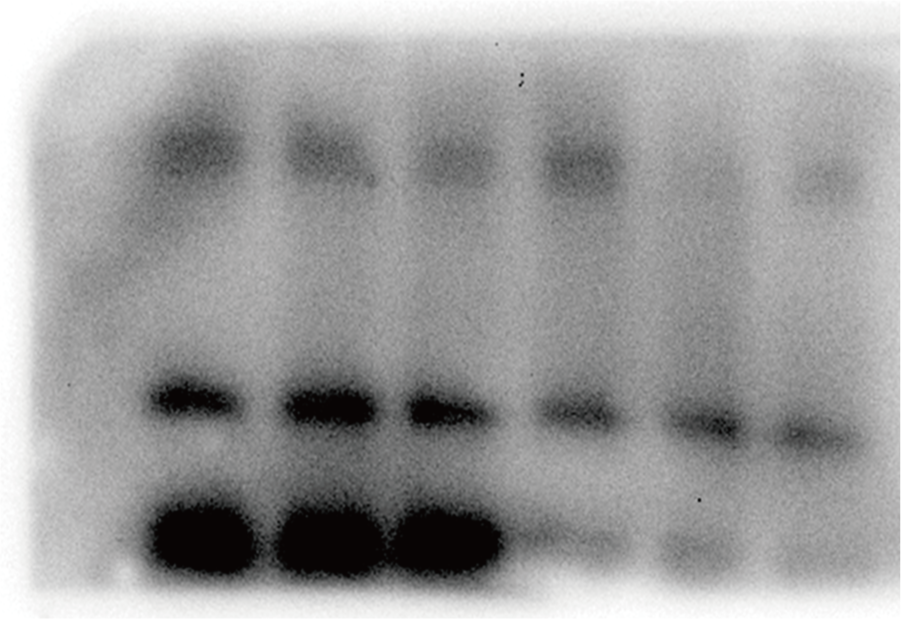

Supplement: Figure 7—source data 2. [file elife-97854-fig7-data2.zip › Proglucagon-7F.tif]

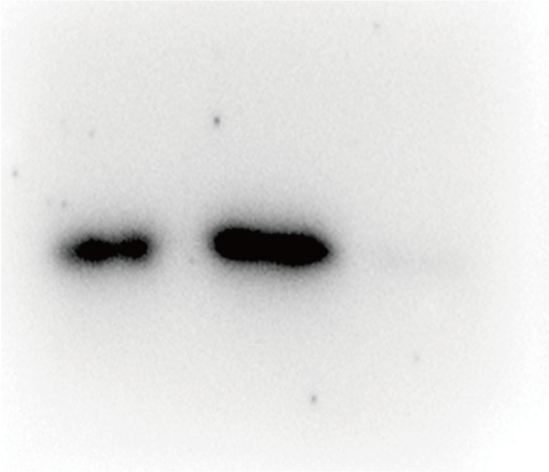

Supplement: Figure 7—source data 2. [file elife-97854-fig7-data2.zip › Proglucagon-7I.tif]

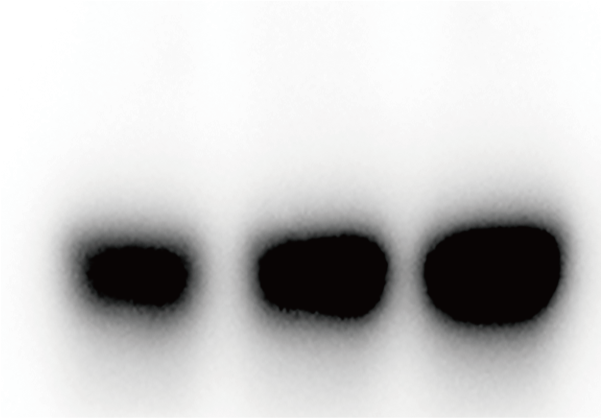

Supplement: Figure 7—source data 2. [file elife-97854-fig7-data2.zip › pS6-7C.tif]

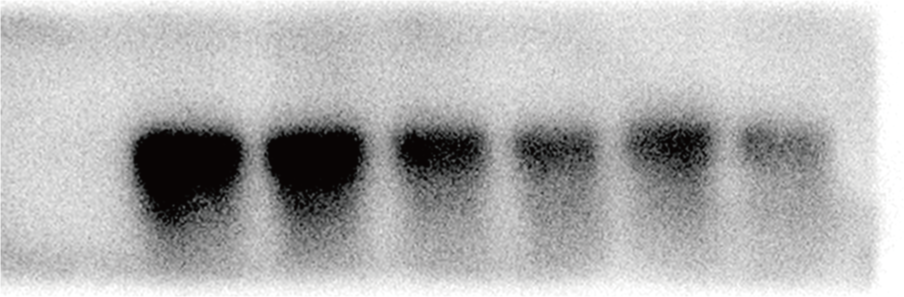

Supplement: Figure 7—source data 2. [file elife-97854-fig7-data2.zip › pS6-7F.tif]

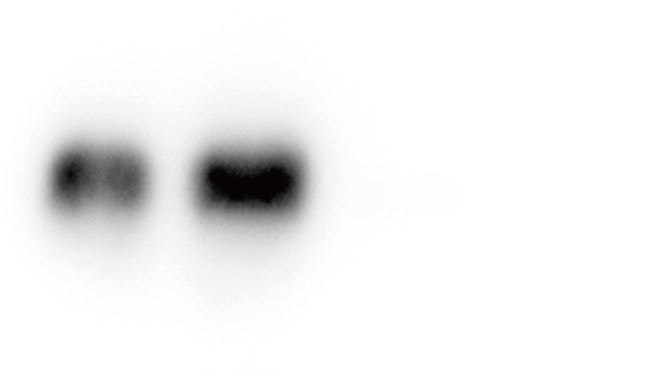

Supplement: Figure 7—source data 2. [file elife-97854-fig7-data2.zip › pS6-7I.tif]

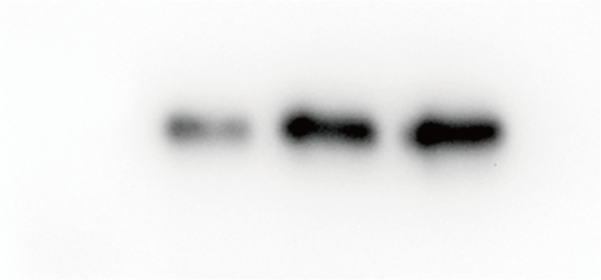

Supplement: Figure 7—source data 2. [file elife-97854-fig7-data2.zip › pS6K-7C.tif]

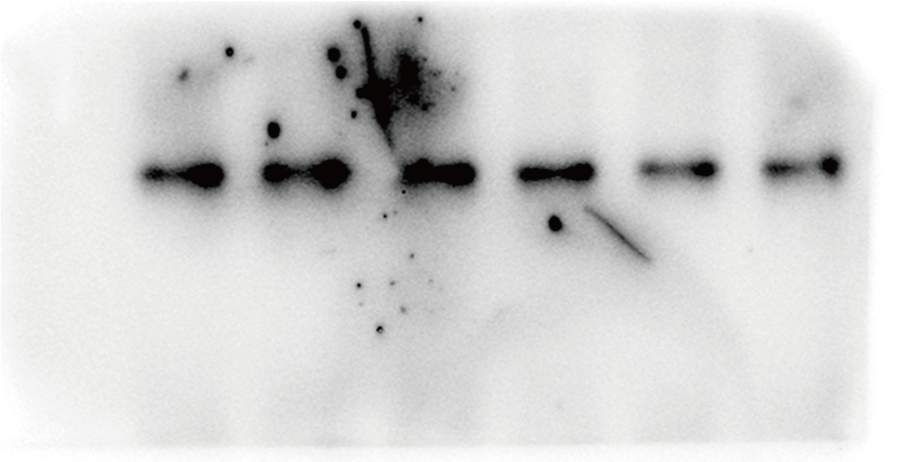

Supplement: Figure 7—source data 2. [file elife-97854-fig7-data2.zip › pS6K-7F.tif]

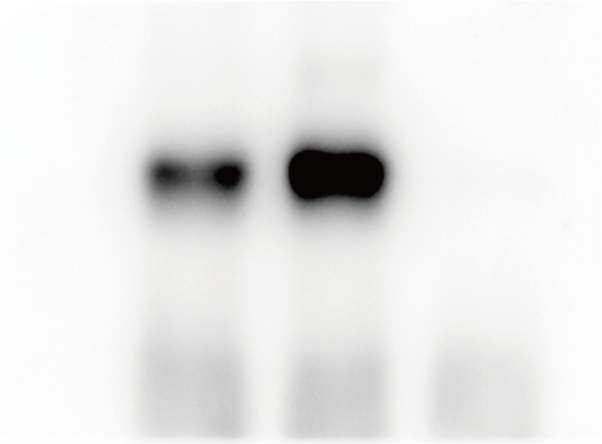

Supplement: Figure 7—source data 2. [file elife-97854-fig7-data2.zip › pS6K-7I.tif]

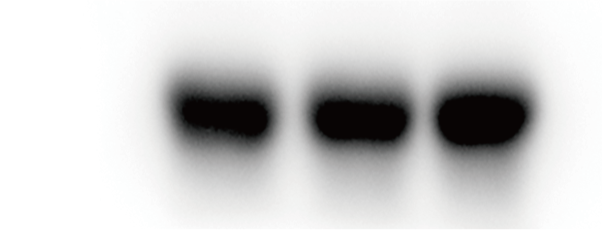

Supplement: Figure 7—source data 2. [file elife-97854-fig7-data2.zip › S6-7C.tif]

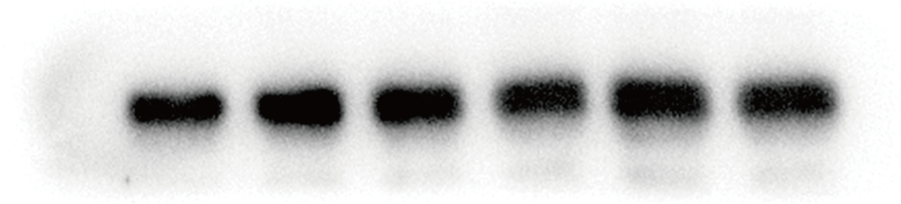

Supplement: Figure 7—source data 2. [file elife-97854-fig7-data2.zip › S6-7F.tif]

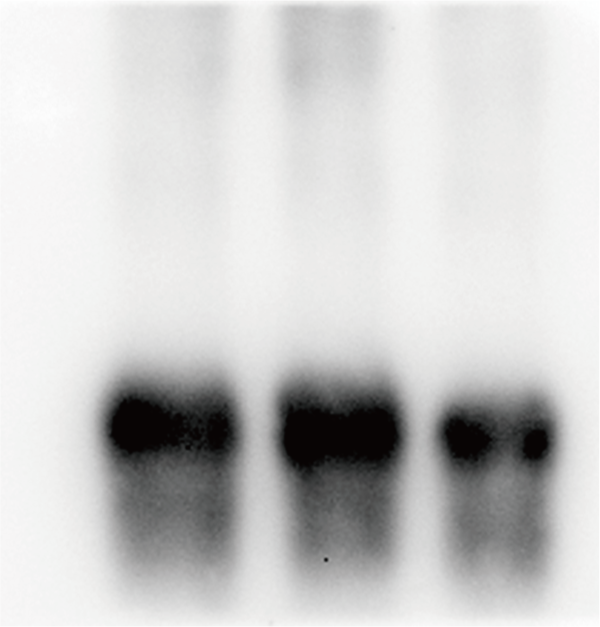

Supplement: Figure 7—source data 2. [file elife-97854-fig7-data2.zip › S6-7I.tif]

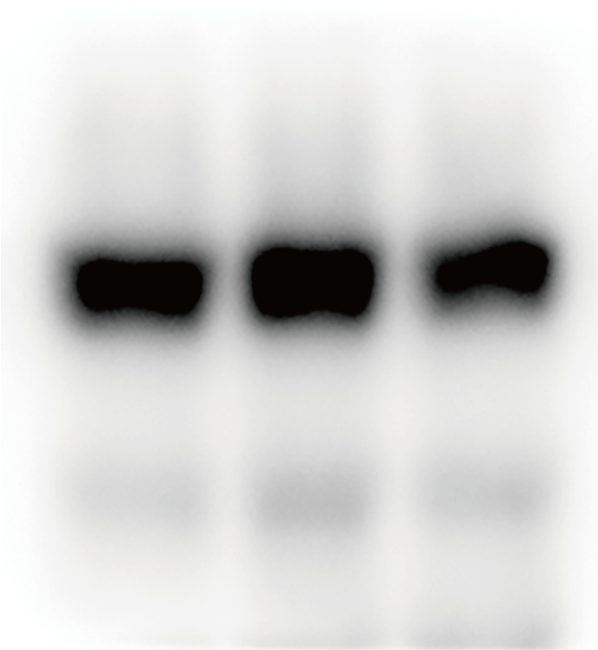

Supplement: Figure 7—source data 2. [file elife-97854-fig7-data2.zip › S6K-7C.tif]

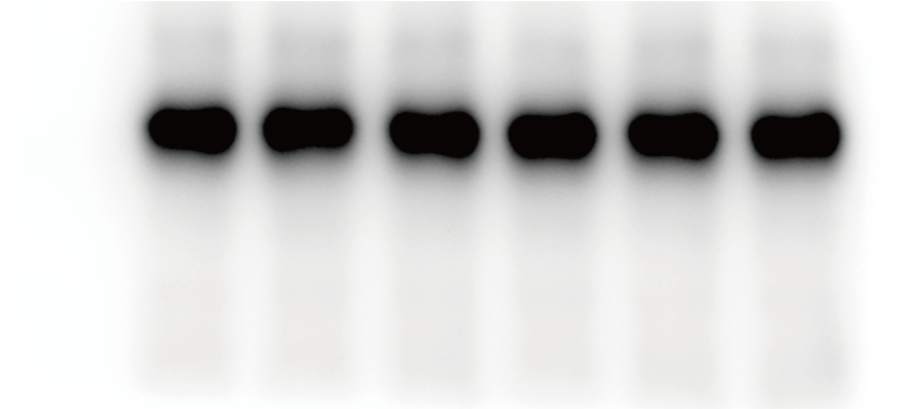

Supplement: Figure 7—source data 2. [file elife-97854-fig7-data2.zip › S6K-7F.tif]

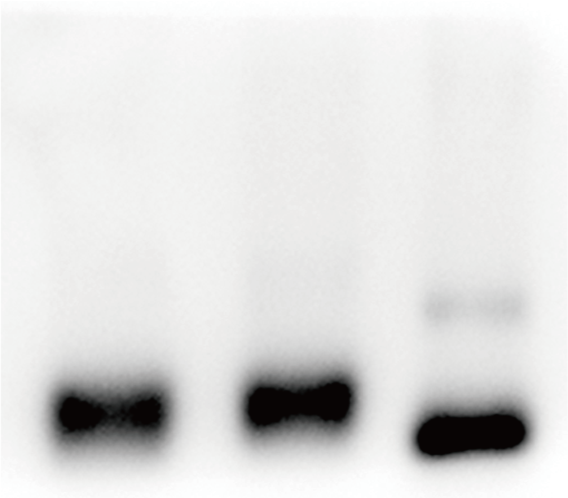

Supplement: Figure 7—source data 2. [file elife-97854-fig7-data2.zip › S6K-7I.tif]

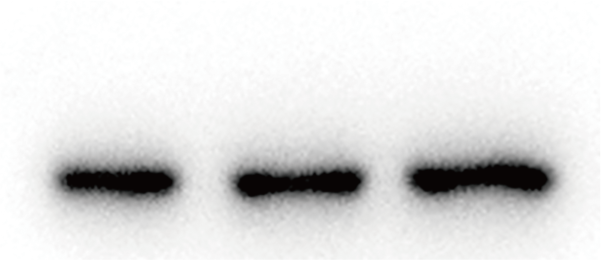

Supplement: Figure 7—source data 2. [file elife-97854-fig7-data2.zip › ╬▓-actin-7C.tif]

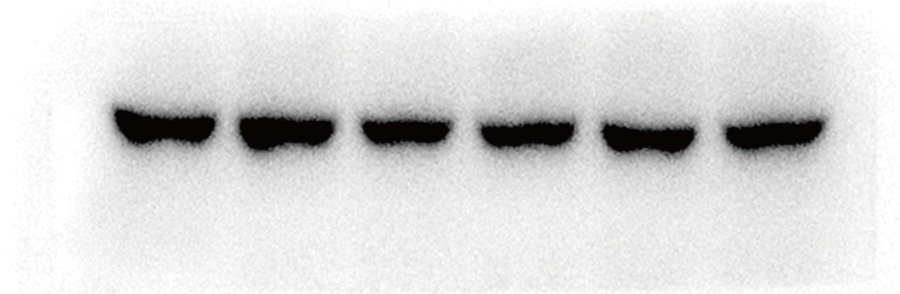

Supplement: Figure 7—source data 2. [file elife-97854-fig7-data2.zip › ╬▓-actin-7F.tif]

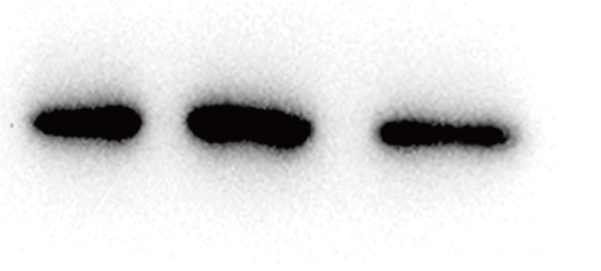

Supplement: Figure 7—source data 2. [file elife-97854-fig7-data2.zip › ╬▓-actin-7I.tif]
