# Supplementary material for: Mechano-regulation of GLP-1 production by Piezo1 in intestinal L cells
Source: eLife. 2024 Nov 7;13:RP97854. doi: 10.7554/eLife.97854 (PMC11542922; doi:10.7554/eLife.97854)
Supplement: Figure 6—source data 1. [file elife-97854-fig6-data1.zip › Figure6-source data 1.pdf]

Figure 6D

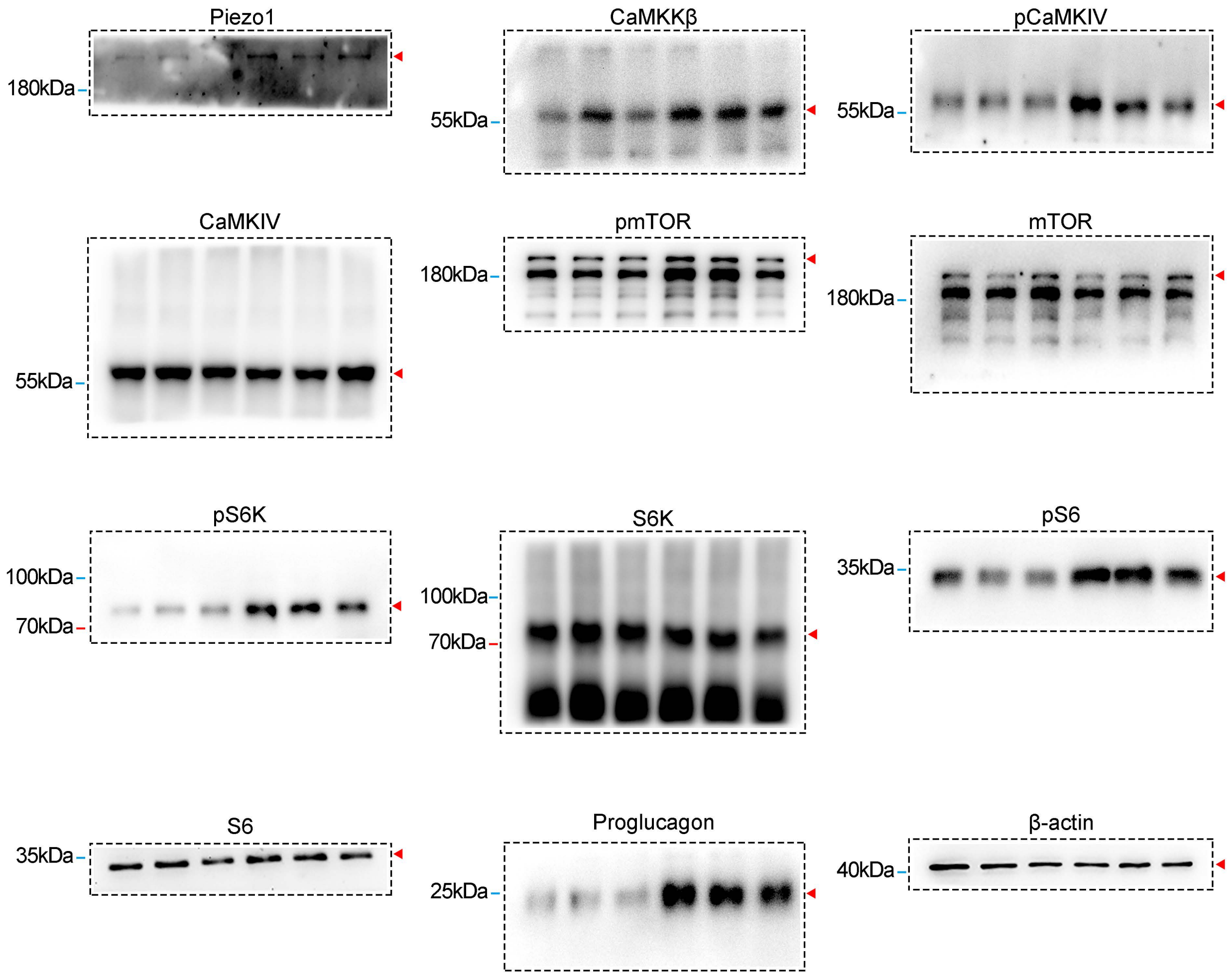

Figure 6, Source Data 1. Original membranes corresponding to Figure 6, panel D, show the treatment of STC-1 cells. The first three lanes represent cells treated with GFP, while lanes 4, 5, and 6 correspond to cells treated with *Piezo1*-GFP.

Figure 6H

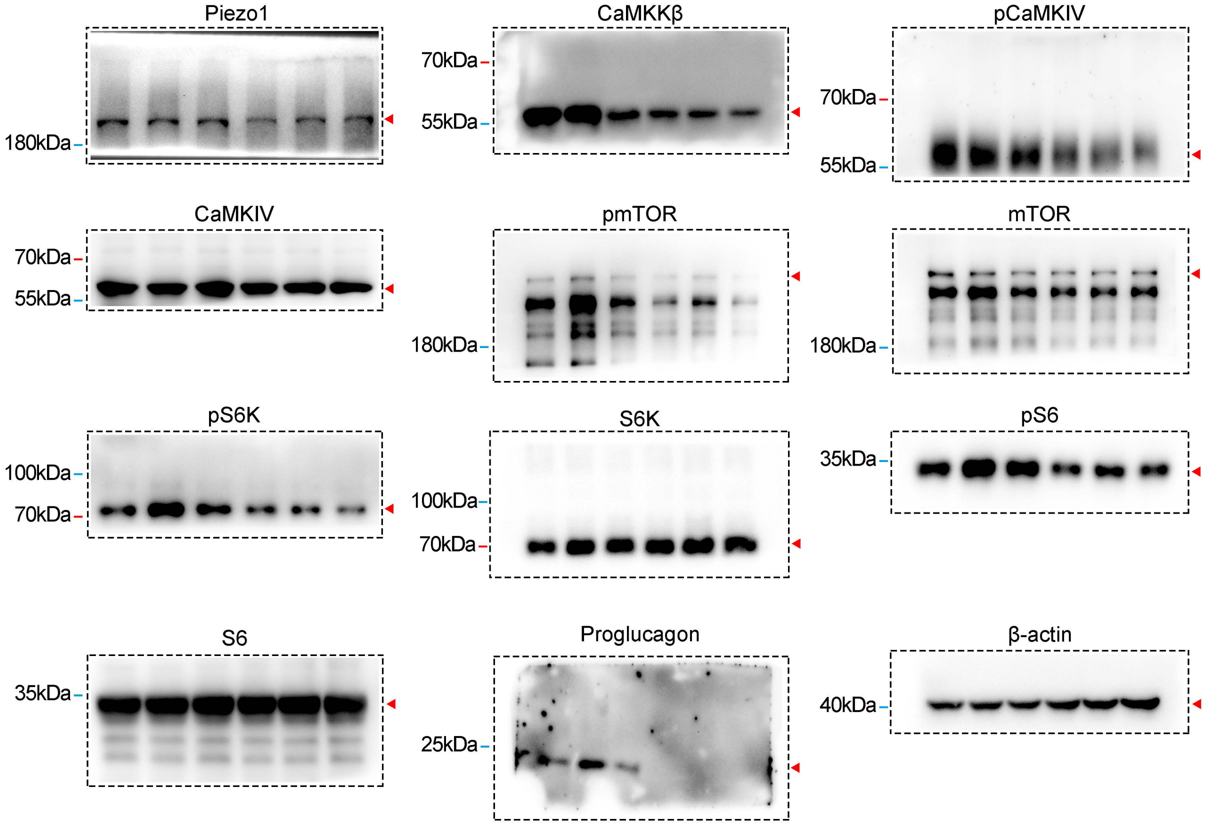

Figure 6, Source Data 1. Original membranes corresponding to Figure 6, panel H, depicting the treatment of STC-1 cells. The first three lanes represent control treatments, while lanes 4, 5, and 6 correspond to sh-*Piezo1* treatments.
