# Supplementary material for: Mechano-regulation of GLP-1 production by Piezo1 in intestinal L cells
Source: eLife. 2024 Nov 7;13:RP97854. doi: 10.7554/eLife.97854 (PMC11542922; doi:10.7554/eLife.97854)
Supplement: Figure 7—source data 1. [file elife-97854-fig7-data1.zip › Figure7-source data 1.pdf]

Figure 7C

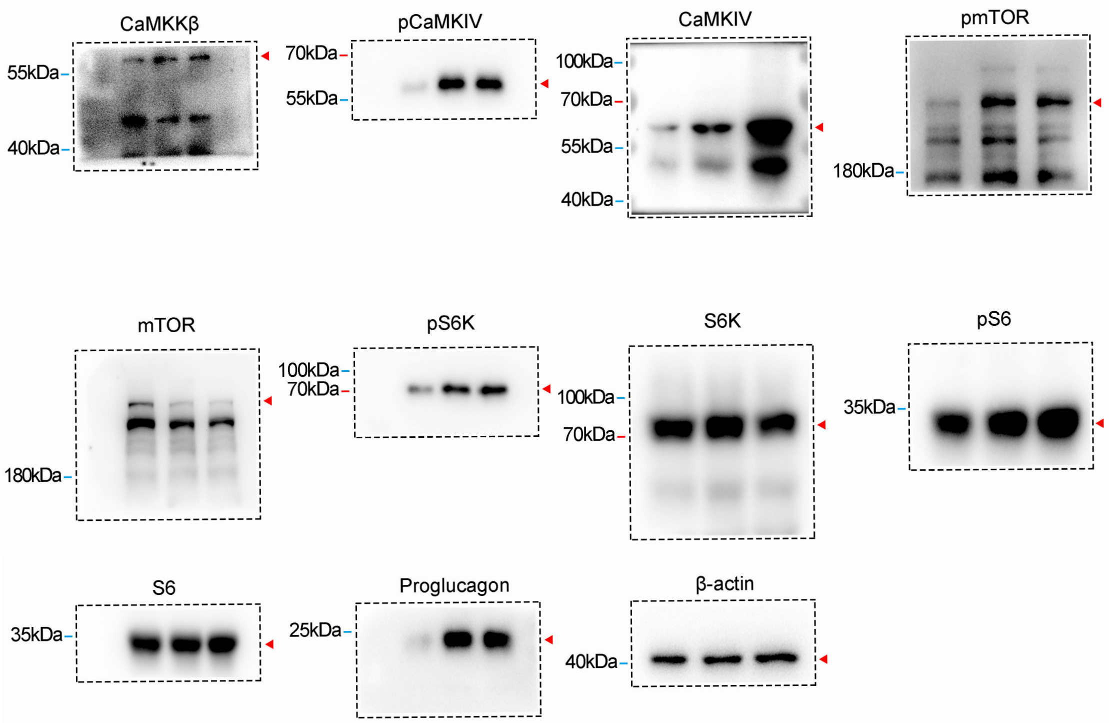

Figure 7, Source Data 1. Original membranes associated with Figure 7, panel C, showing the treatment of STC-1 cells. The first lane represents GFP treatment, the second lane corresponds to treatment with a *CaMKKβ* overexpression plasmid, and the third lane reflects treatment with a *CaMKIV* overexpression plasmid.

Figure 7F

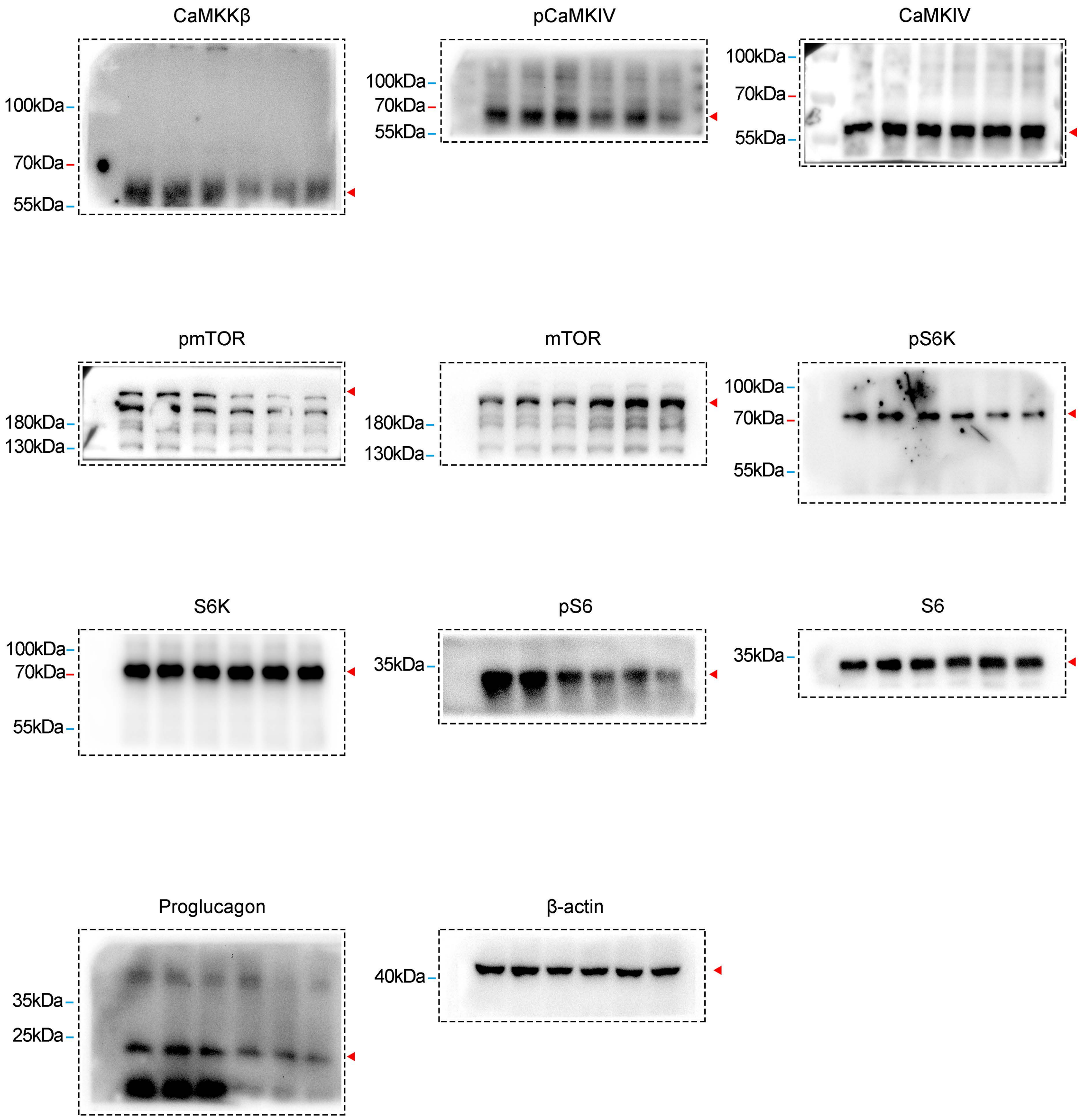

Figure 7, Source Data 1. Original membranes shown in Figure 7, panel F, depict the treatment of STC-1 cells. The three lanes on the left were treated with DMSO, while the three lanes on the right received treatment with STO-609.

Figure 7I

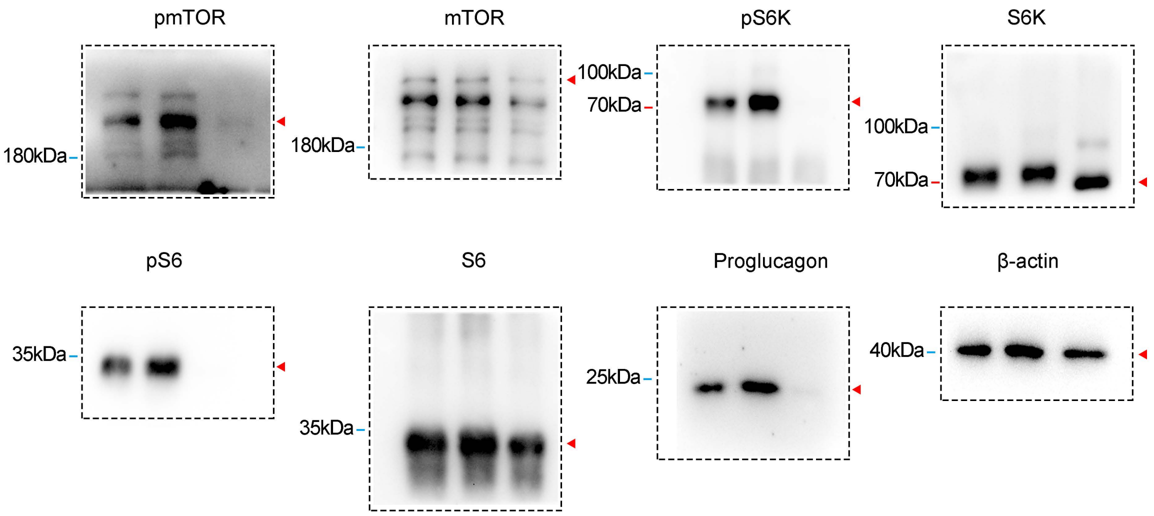

Figure 7, Source Data 1. Original membranes corresponding to Figure 7, panel I, illustrating the treatment of STC-1 cells. The first lane represents DMSO treatment, the second lane corresponds to Yoda1 treatment, and the third lane shows the effects of Yoda1 combined with Rapamycin treatment.
